# Enhanced Intranasal Delivery of Atorvastatin via Superparamagnetic Iron-Oxide-Loaded Nanocarriers: Cytotoxicity and Inflammation Evaluation and In Vivo, In Silico, and Network Pharmacology Study for Targeting Glioblastoma Management

**DOI:** 10.3390/ph18030421

**Published:** 2025-03-16

**Authors:** Kristina Zarif Attalla, Doaa H. Hassan, Mahmoud H. Teaima, Carol Yousry, Mohamed A. El-Nabarawi, Mohamed A. Said, Sammar Fathy Elhabal

**Affiliations:** 1 Department of Pharmaceutics, College of Pharmaceutical Sciences and Drug Manufacturing, Misr University for Science and Technology, Giza 12566, Egypt; doaa.hassan@must.edu.eg; 2 Department of Pharmaceutics and Industrial Pharmacy, Faculty of Pharmacy, Cairo University, Cairo 11562, Egypt; mahmoud.teaima@pharma.cu.edu.eg (M.H.T.); carol.yousry@pharma.cu.edu.eg (C.Y.); mohamed.elnabarawi@pharma.cu.edu.eg (M.A.E.-N.); 3 Department of Pharmaceutics and Industrial Pharmacy, School of Pharmacy, Newgiza University, km. 22 Cairo-Alex Road, Giza P.O. Box 12577, Egypt; 4 Pharmaceutical Chemistry Department, Faculty of Pharmacy, Egyptian Russian University, Badr City, Cairo 11829, Egypt; mohamed-adel@eru.edu.eg; 5 Department of Pharmaceutics and Industrial Pharmacy, Faculty of Pharmacy, Modern University for Technology and Information (MTI), Mokattam, Cairo 11571, Egypt

**Keywords:** intranasal hydrogel, nanocarriers, atorvastatin, superparamagnetic iron oxide, glioblastoma multiforme, enhanced permeation and retention effect and brain targeting

## Abstract

**Objective**: This study aims to develop an intranasal (IN) delivery system for glioblastoma multiforme (GBM) management using repurposed superparamagnetic iron-oxide (SPION) loaded with atorvastatin (ATO)-nanostructured lipid carrier (NLC). **Methods**: Emulsification and ultrasonication were used to formulate ATO-NLCs, and the best formula was loaded with SPION to make the final atorvastatin/superparamagnetic iron oxide-loaded nanostructured lipid carrier (ASN) formulation. Entrapment efficiency (EE%), particle size (PS), zeta potential (ZP), and drug release after 6 h (Q6h) were evaluated for NLCs. ASN was tested for cytotoxicity on T98G cancer cells, and the cell cycle was examined to determine cell death. Furthermore, the ability of the optimal formulation to suppress the levels of inflammatory biomarkers was investigated in Lipopolysaccharide (LPS)-induced inflammation. The brain-targeting behavior of IN-ASN was visualized in rabbits via confocal laser scanning microscopy (CLSM). **Results**: The optimum NLC exhibited a spherical shape, EE% of 84.0 ± 0.67%, PS of 282.50 ± 0.51 nm, ZP of −18.40 ± 0.15 mV, and Q6h of 89.23%. The cytotoxicity of ASN against cancer cells was 4.4-fold higher than ATO suspension, with a 1.3-fold increment in cell apoptosis. ASN showed significantly reduced pro-inflammatory biomarkers (IL-β, IL-6, TNF-α, TLR4, NF-қB), whereas CLSM revealed enhanced brain delivery with no observed histopathological nasal irritation. The in silico analysis demonstrated enhanced ATO-ADME (absorption, distribution, metabolism, and excretion) properties, while the network pharmacology study identified 10 target GBM genes, among which MAPK3 was the most prominent with a good binding score as elucidated by the simulated docking study. **Conclusions**: These findings may present ATO/SPION-NLCs as significant evidence for repurposing atorvastatin in the treatment of glioblastoma multiforme.

## 1. Introduction

Glioblastoma multiforme (GBM) represents one of the most prevalent and lethal human cancers, responsible for almost 60% of adult primary brain tumors. Chemoradiotherapy and surgical resection, which are restricted by GBM’s diffuse and infiltrative nature, provide a median survival rate of about 14 months [1]. Chemotherapy in GBM is particularly ineffective because of the fast proliferation of GBM cells and the restricted entry of systemically administered drugs into the brain tissue due to the blood–brain barrier (BBB) [2,3]. Thus, the chance of GBM recurrence, progression, or mortality cannot be considerably reduced, not even with completed therapy. This stimulated an interest in the biological behavior of GBM and the investigation of innovative treatment protocols, including the use of alternative active pharmaceutical ingredients (APIs) for chemotherapy in GBM.

The process of developing new pharmaceuticals to enhance cancer treatment is both time-consuming and costly. Instead, drug repurposing, or repositioning, has effectively shown great success in circumventing the protracted drug development process. Over several years, drug repurposing has been implemented in the treatment of cancer. Despite their initial indications, these drugs are now extensively used in the therapy of cancer [4].

Statins, a popular class that inhibit the activity of 3-hydroxy-3-methylglutaryl-coenzyme A (HMG-CoA) reductase, which alters HMG-CoA into mevalonate (MVA) for hyperlipidemia treatment [5]. Besides lowering cholesterol, it also decreases the key substances needed for cholesterol production, such as MVA, farnesyl pyrophosphate (FPP), and geranylgeranyl pyrophosphate (GGPP). These molecules are essential for critical biological functions such as cell signaling, synthesis of protein, and progression of the cell cycle [6]. The production of lipid moieties required for the replication in GBM cells is primarily based on the MVA pathway; thus, GBM cell replication and development is inherently susceptible to statins functioning as competitive inhibitors of HMG-CoA reductase enzyme, which controls MVA production [7]. In light of these facts, statins were widely investigated as a promising treatment for brain cancer.

The statins suggested anticancer effect is attributed to their capability to inhibit angiogenesis and activate apoptosis, which results in the reduction in tumor development and metastases. Previous research found that statins could decrease the risk of many types of cancer, including hepatocellular carcinoma [8], gastrointestinal cancer [9], lung cancer [10], breast cancer [11], and prostate cancer [12].

Atorvastatin (ATO) has poor bioavailability (≈12%) after oral intake, attributable to its significant first-pass effect and limited aqueous solubility (0.1 mg/mL) [13]. ATO is a synthetic lipophilic statin that can pass the BBB through passive diffusion [1]. Previous studies demonstrated that ATO may considerably impede the invasive ability of glioma cells and stimulate apoptosis in tumors of glioblastoma spheroids grown on fibrin gel [5]. Furthermore, a real-time PCR investigation of U87 glioma spheroids found that ATO promoted cell death by up-regulating caspase-3 and caspase-8 while down-regulating B-cell lymphoma 2 (Bcl-2) [5]. Also, Yongjun et al. [14] found that ATO decreased the microglial production of membrane type 1 metalloproteinase (MT1-MMP), which decreased glioma migration and invasion. Additionally, ATO may lower interleukins and pro-inflammatory protein expression [15].

Recently, many cancer-related studies have investigated the use of nanomedicine as a therapeutic and diagnostic approach. Among these, different nanosystems such as liposomes, nanoparticles, lipid nanostructure, and micelles were actively used for brain delivery of anticancer agents [16,17,18]. Nanoparticles possess a significant advantage in their ability to traverse the BBB.

Moreover, owing to their physicochemical characteristics, biological compatibility, capacity for self-assembly, and adaptability with surface modification for tumor targeting, nanoparticles have significant promise in the GBM treatment [19]. Additionally, they may enhance the therapeutic efficacy and alleviate the systemic side effects of chemotherapeutic drugs.

Nanostructured lipid carriers (NLCs) represent the subsequent evolution of solid-lipid nanoparticles (SLNs) [20]. NLCs had an amorphous structure and a reduction in matrix perfection than SLNs, which led to increased loading of drug capacity and diminished the leakage of drugs from the matrix [21]. Furthermore, NLC demonstrates exceptional biocompatibility and biodegradability, controls the rate of drug release, and can maintain the stability of encapsulated drugs [13].

NLCs are known for improving the solubility of drugs with limited aqueous solubility, such as atorvastatin. Assisted lipid matrix inclusion can enhance drug dissolution and absorption by overcoming their limited water solubility [13]. Lipidic nanocarrier systems, including nanostructured lipidic carriers (NLCs), are applicable in nasal drug delivery owing to their numerous intrinsic properties, such as improved drug diffusion and bioavailability, safeguarding drug molecules from enzymatic degradation, and regulating the release of drugs [22]. The good choice of excipients is a significant factor that equally affects the performance and stability of the formulated systems. Thus, the absence of selectivity and targeting capacity in unfunctionalized NLC requires further adjustments to enhance the targeting delivery of encapsulated drugs [23].

Superparamagnetic iron oxide nanoparticles (SPIONs) serve as an effective drug delivery method to address the limitations linked to NLC due to their unique magnetic behavior. Fe_3_O_4_ (magnetite) nanoparticles have garnered considerable attention recently owing to their distinctive physicochemical properties and a wide range of applications, including cell imaging, cancer thermotherapy, and controlled drug delivery [12]. SPIONs provide several benefits due to their ease of synthesis, biocompatibility, and potential for surface modification using a variety of chemical agents [24]. The drug can be directly affixed to the particle’s surface in a variety of nanocarriers that have SPION and the drug in a combination for magnetic drug targeting [25,26,27].

The nasal channel is utilized as a non-invasive pathway for central nervous system drug delivery, attributed to its extensive surface area, rapid flow of blood, permeable endothelial membrane, and absence of pre-systemic metabolism.

In addition, the nasal drug delivery will bypass the BBB through the lymphatic route or olfactory route and so be transported to the cerebrovascular system for brain targeting. All these parameters would contribute to increased brain bioavailability at lower drug doses and less systemic adverse effects [28,29]. In addition, the lipid-based composition and low toxicity of NLC could prevent any irritation or injury to the nasal mucosa. It also aids in addressing some nasal route challenges, including insufficient penetration and enzymatic degradation [20]. Several studies have employed SLNs, Self-Nanoemulsifying Drug Delivery Systems (SNEDDSs), and cyclodextrin-based nanosponges to increase ATO oral absorption, but none have examined NLCs’ intranasal bioavailability [29,30,31].

This study sought to formulate and assess a nanostructured lipid carrier (NLC) with the aid of superparamagnetic iron oxide (SPION) for nose-to-brain delivery of ATO. The formulated system would help in improving the permeation of the drug through the nasal cavity and increment ATO delivery to the brain which could ultimately improve the drug’s effectiveness. To our knowledge, this is the only study on preparing intranasal ATO/SPION-loaded NLC for targeting the brain and glioblastoma treatment. This study presents the first intranasal SPION-loaded Atorvastatin Nanostructured Carrier (ASN) system for glioblastoma management [32]. Unlike prior studies that focused on oral SNEDD [33], our research innovatively combines nanotechnology and magnetic targeting with an intranasal route to bypass the blood–brain barrier (BBB), enhancing brain bioavailability of arranging. The dual-functional approach of combining SPION for magnetic guidance and NLCs for improved atorvastatin solubility ensures higher anticancer efficacy and lower systemic toxicity.

This study incorporates network pharmacology and in silico studies to validate potential molecular targets of atorvastatin against glioblastoma.

To achieve our goal, a 3^2^-full factorial design was developed to optimize the formulations of NLCs. The impact of the different formulation variables was statistically analyzed relative to the values of their particle size (PS), entrapment efficacy (EE%), zeta potential (ZP), polydispersity index (PDI), and percent released after 6 h (Q6h) and optimized to generate the optimum formula. Finally, the optimal system with the highest desirability factor was physically loaded with SPIONs to improve their brain-targeting behavior. The cytotoxic effect of ATO/SPION-loaded NLC (ASN) on human glioblastoma cell line T98G was evaluated, and the cell cycle was investigated to determine the exact mechanism of cell death. Furthermore, the capability of the optimal formulation to suppress the inflammatory biomarkers was investigated in Lipopolysaccharide (LPS)-induced inflammation. The brain-targeting behavior of ASN after intranasal administration to rats was visualized via confocal laser scanning microscopy. Finally, an in silico study was performed to assess the chemical compatibility between ATO and NLC optimum formulation and a network pharmacology study was conducted to assess the antitumor activity and target prediction pathways of ATO.

## 2. Results and Discussion

### 2.1. Experimental Design

Design-Expert^®^ 13 software was utilized to study the effect of different variables of formulations on the characteristics of ATO-NLC. Nine formulations corresponding to a 3^2^ full factorial design (Table 1) were suggested to study the effect of the lipid type and Surface-Active Agent (SAA) type, each at three levels. The prepared formulation was evaluated in terms of EE% (Y_1_, %), PS (Y_2_, nm), ZP (Y_3_, mV), and Q6h (Y_4_, %).

Statistical analysis of the response data via ANOVA was fitted to the main effects model in EE%, whereas the two-factor interaction (2FI) model was selected for the statistical analysis of PS, ZP, and Q6h. The models were selected to offer the best fit for the response data as denoted by the high R^2^ value (approaching 1), high adequate precision (>4), and the good agreement between the adjusted and predicted R^2^ values with a difference in less than 0.2 as shown in (Table 2) [28].

#### 2.1.1. Impact of Formulation Variables on EE%

The entrapment efficiency of the drugs in lipid nanocarriers is mainly governed by the drug’s solubility in the lipid matrix. The lipophilic characteristics of ATO, shown by its partition coefficient of log P(octanol/water) = 5.39, promote its association with lipid carriers, hence enhancing drug loading [27,31]. Incorporating liquid lipids into solid lipids reduces their crystallinity and introduces defects in the crystal lattice of NLC formulations. This allows a larger area inside the nanoparticles for integrating drugs, resulting in improved drug entrapment and loading [13]. Furthermore, Compritol 888, as a solid lipid component in all formulations, likely enhances drug entrapment owing to its substantial quantities of mono-, di-, and triglycerides, which promote the solubilization of drugs [27].

**Figure 1:**
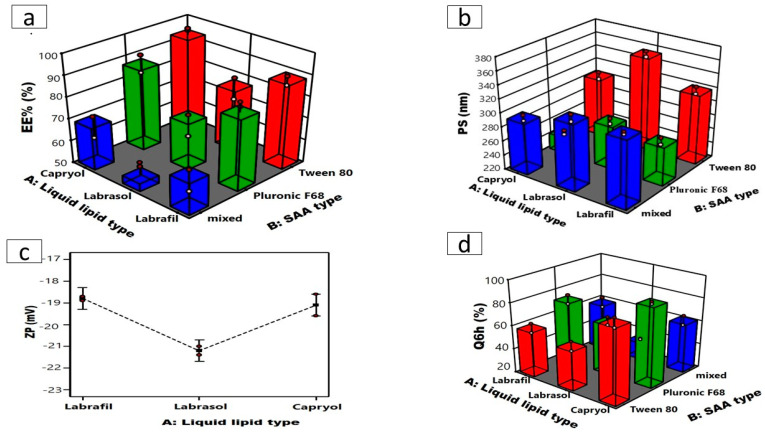
Three-dimensional plots for the effects of liquid lipid type (X_1_) and SAA type (X_2_) on (**a**) EE%, (**b**) PS, (**c**) line plot for the effect of liquid lipid type (X_1_) on ZP, and (**d**) Q6h of ATO-loaded NLCs.

Table 1 and (Figure 1a) illustrate that the EE% of the formed ATO-NLC varied from 58.05 ± 0.32% to 92.850 ± 0.52%. ANOVA statistical analysis indicated that the main-effect model is the most suitable for data analysis. ANOVA showed that the liquid lipid type (X_1_) substantially affected ATO encapsulation within the NLC matrix (*p* = 0.0008). It is well-documented that the physicochemical features of NLCs are impacted by the type of liquid lipid utilized, which can be related to many variables, such as the oil’s viscosity, the value of HLB, compatibility of liquid and solid lipids, and the drug’s solubility in the selected lipid [34]. NLCs prepared using Capryol^®^ 90 exhibited higher EE% than those formulated using Labrafil and Labrasol. ATO is a lipophilic drug with higher affinity and improved miscibility within nonpolar, lipophilic components [35]. Lipophilic Capryol 90, with a low HLB value of 5 compared to Labrafil and Labrasol (with respective HLB values of 9 and 12), may enhance ATO miscibility and subsequently load into the NLC matrix [36,37,38].

Conversely, the SAA type (X_2_) significantly influenced EE% (*p* < 0.0001). Tween 80 presented NLCs developed with greater EE% than those developed with Pluronic F68, as demonstrated in (Figure 1a) [39], which could also be related to the HLB values of the SAAs, which were 15 and 29 for tween and Pluronic F68, respectively [40,41]. SAAs exhibiting lower HLB values demonstrate increased hydrophobicity and a propensity to encapsulate lipophilic drugs, whereas hydrophilic SAAs with more polar head groups, such as Pluronic F68, form less rigid lipid nanocarriers. This could allow the drugs to escape into the external aqueous phase during preparation, thereby lowering the drug’s entrapment [42].

Conversely, NLCs produced utilizing a combination of Tween 80 and Pluronic F68 exhibited the lowest encapsulation efficiency (EE%) regardless of the liquid lipid utilized. These results agree with the findings of Elmowafy et al. [13], which may be explained by the fact that the binary combination of surfactants dissolves ATO better than each surfactant alone. Thus, ATO was extracted from the lipid phase into the SAA solution, resulting in a reduced EE% within the NLCs.

#### 2.1.2. Impact of Formulation Variables on PS and PDI

The PS is a fundamental aspect during the formulation of nanosystems intended for intranasal administration. PS directly affects the potential of nanosystems to access the brain by facilitating the direct passage of drugs from the nasal cavity to the brain via trigeminal and olfactory neurons while adhering to the olfactory epithelium; hence, the mucosal of the nasal cavity enhances its contact time [28]. Furthermore, NLCs with smaller particle sizes stay longer in the bloodstream and minimize complement recognition in the blood, enhancing the effect of the encapsulated drugs and prolonging their effect [43]. The mean PS of the formulated ATO- NLC extended from 282.5 ± 0.51 to 354.1 ± 2.72 nm with PDI values ranging from 0.48 ± 0.02 to 0.57 ± 0.03 as illustrated in Table 1. The 2FI model was selected as the best-fitting model, which demonstrated a significant impact of the liquid lipid type (X_1_; *p* < 0.0001) and SAA type (X_2_; *p* < 0.0001) on the PS as shown in (Figure 1b). NLCs formulated using Labrasol as the liquid lipid showed the highest PS in comparison to Capryol 90 and Labrafil. Increasing lipid molecular weight increases nanodispersion viscosity, possibly facilitating PS aggregation and amplification [21], who declared that the lipid’s molecular weight and viscosity affected the particle size. Labrasol with higher molecular weight (Mw = 400) may occupy a larger volume, resulting in bigger NLC particles than Labrafil and Capryol 90 (Mw = 300 and 202.94). Additionally, Labrasol is more viscous (80–110 cp) than Labrafil (70–90 cp) and Capryol 90 (20 cp) [21,36]. Higher viscosity would ultimately impede ultrasonication’s capacity to reduce the size of the particles of the formed NLCs, leading to larger particle sizes. On the other hand, NLC formulated using Tween 80 exhibited a larger particle size compared to those formulated using Pluronic F68 [44], a reduction in the hydrophilic component of SAA with a lower HLB value is associated with an increase in PS of the nanosystems. Thus, Tween 80 with the lowest HLB value (≈15) resulted in NLCs with the largest PS.

PDI values assess sample homogeneity and PS distribution around the mean. PDI values of ˂ 0.7 indicate a confined particle size distribution. In our study, ATO-NLCs were slightly polydisperse within limits with values less than 0.574 [41].

#### 2.1.3. Impact of Formulation Variables on ZP

The ZP values of the developed ATO-NLC are measured to provide insights into the stability of the formulation. Table 1 illustrates that the ZP of the formulations varied from −17.2 ± 0.29 to −22.2 ± 0.25 mV. The negativity of the particles is correlated with acidic groups of the liquid and solid lipids. Statistical analysis via ANOVA showed that only liquid lipid type (X_1_) (*p* = 0.0223) has a significant effect on ZP values, with Labrasol having the highest ZP values (Figure 1c). Previous studies indicate that a reduction in electrophoretic mobility may lead to an increase in the negativity of the particle’s surface charge [45]. Labrasol has the largest particle size, as previously mentioned, which may have led to a reduction in the electrophoretic mobility of labrasol-based NLC and enhanced ZP values. Statistical analysis also showed that the effect of SAA type (X_2_) on the ZP was insignificant (*p* = 0.5850) [37].

#### 2.1.4. Impact of Formulation Variables on Q6h

The release study test evaluated ATO-NLCs formulation and predicted lipid nanocarrier performance in vivo following intranasal injection.

As illustrated in Table 1, NLC formulations released 35.40 ± 1.77% to 89.23 ± 1.09% of the drug after 6 h (Q6h). The statistical analysis showed that both liquid lipid (X_1_, *p* < 0.0001) and SAA (X_2_, *p* ˂ 0.0001) types significantly affected Q6h (Figure 1d). Q6h was significantly greater for Capryol 90-based NLCs than labrafil and labrasol. Q6h was statistically higher for Capryol 90-based NLCs compared to labrafil- and labrasol-based NLCs. This can be attributed to the smaller sizes of the NLCs formulated using Capryol 90. Nanoparticles with smaller particle sizes possess a higher surface area that is available for drug release, resulting in a faster dissolution profile and higher Q6h [44].

Likewise, Pluronic F68-based NLCs with reduced particle size demonstrated a greater release rate in comparison to Tween 80-based NLCs.The release profile features of diverse formulations using different surfactants exhibited a link between the percentage enhancement of drug release and the HLB of surfactant-active agents [43].

#### 2.1.5. Selection of the Optimized ATO-Loaded NLCs Formula

The formulation factors were optimized to find the formula with the lowest PS and highest EE%, ZP, and Q6h. Design Expert software chose ATO-NLC F2 as the optimum formula, with a 0.821 desire factor, to be combined with SPION for further investigation. Capryol 90 served as a liquid lipid, Pluronic F68 functioned as a non-ionic surfactant, while Compritol 888 ATO acted as a solid lipid, and Lecithin was used to construct the optimal formula (ATO-NLC F2). ATO-NLC F2 demonstrated observed parameters of 84.0 ± 0.67% for EE%, 282.50 ± 0.51 nm for PS, −18.40 ± 0.15 mV for ZP, and 89.23 ± 1.09% for Q6h, respectively.

### 2.2. Characterization of ATO-NLC F2 Loaded with SPION (ASN)

#### 2.2.1. Entrapment Efficiency, Particle Size, Zeta Potential, and In Vitro Released After 6 H

SPION was fabricated with a small PS of 10.32 ± 1.04 nm and a positive ZP of 21.3 ± 0.68. The incorporation of SPION with ATO-NLC F2 (ASN) resulted in a slight rise in PS from 282.50 ± 0.51 nm to 294.4 ± 0.81 nm, accompanied by a slight enhancement in EE%. The ZP value of the formulated ASN was reduced (−14.8 mV) compared to ATO-NLC F2, which may be related to the integration of positively charged iron oxide nanoparticles into the NLC surface [27]. Finally, Q6h from ASN was also reduced to 78 ± 0.16%, which is likely attributed to the magnetic attraction between the adjacent NLCs when magnetic nanoparticles are incorporated into the structure of NLCs, resulting in a slower release rate [21,46] (Table 1).

#### 2.2.2. Magnetic Behavior

**Figure 2:**
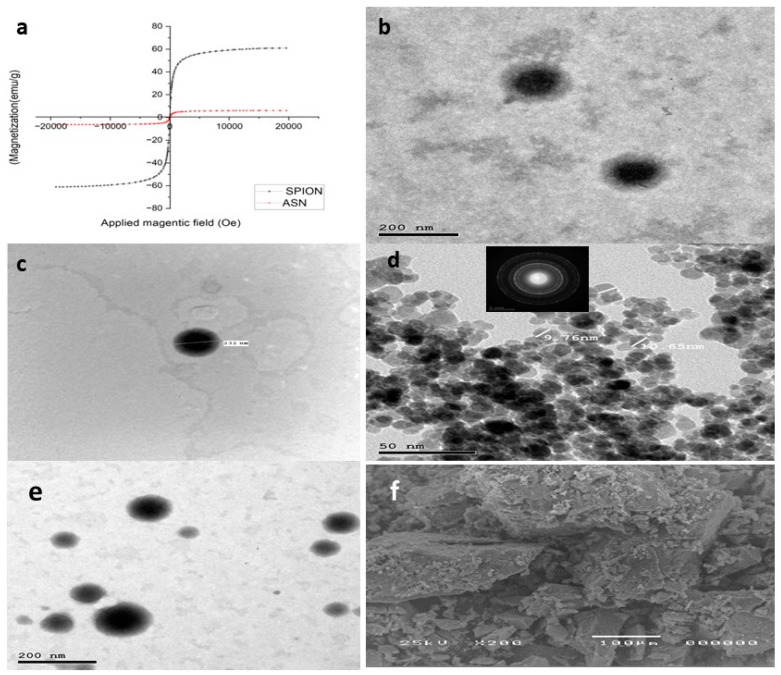
(**a**) Vibrating sample magnetometer (VSM), (**b**,**c**) transmission electron microscopy image of ATO-NLC F2, (**d**) SPION, (**e**) ASN, and (**f**) scanning electron microphotograph of SPION.

(Figure 2a) depicts the hysteresis loop profiles for SPIONs and ASN. The saturation magnetization (Ms) values were 61.05 emu g^−1^ and 6.1703 emu g^−1^ for SPIONs and ASN, respectively, indicating their superparamagnetic properties. All the samples’ hysteresis curves originated from the origin (i.e., both remanences were zero). As mentioned earlier by Thapa et al. [47], the tiny diameters of SPIONs contribute to a reduction in oxygen content and an increase in divalent iron ion (Fe^2+^) concentration (lower valence state), which accounts for their high saturation magnetization (61.05 emu g^−1^). Although ASN has a lower saturation magnetization than SPIONs, it nonetheless shows significant magnetic responsiveness to external magnetic fields due to the inclusion of SPIONs with the optimum NLCs.

#### 2.2.3. Morphology Analysis

The transmission electron micrographs indicated that the optimum formula ATO-NLC F2 exhibited nanoscaled particles with a spherical shape and no observable irregularities or aggregates, as presented in (Figure 2b,c). The observed PS agreed with the PS previously measured by Zetasizer. The lipid nanocarriers also show clearly defined boundaries correlated with the amphiphilic nature of the non-ionic SAAs, which triggers the formation of spherical vesicles aiming to reduce surface-free energy [21,48]. At the same time, the TEM pictures of the formed SPIONs showed spherical particles with an average size of 10 nm, as previously estimated. The crystalline structure of SPIONS is shown in the selected area electron diffraction (SAED) image in the inset of (Figure 2d). Lastly, as shown in (Figure 2e), ASN particles showed spherical NPs with SPIONS impacted inside.

The SEM examination of SPIONs with evenly distributed particles throughout the sample is shown in (Figure 2f).

#### 2.2.4. Differential Scanning Calorimetry (DSC)

**Figure 3:**
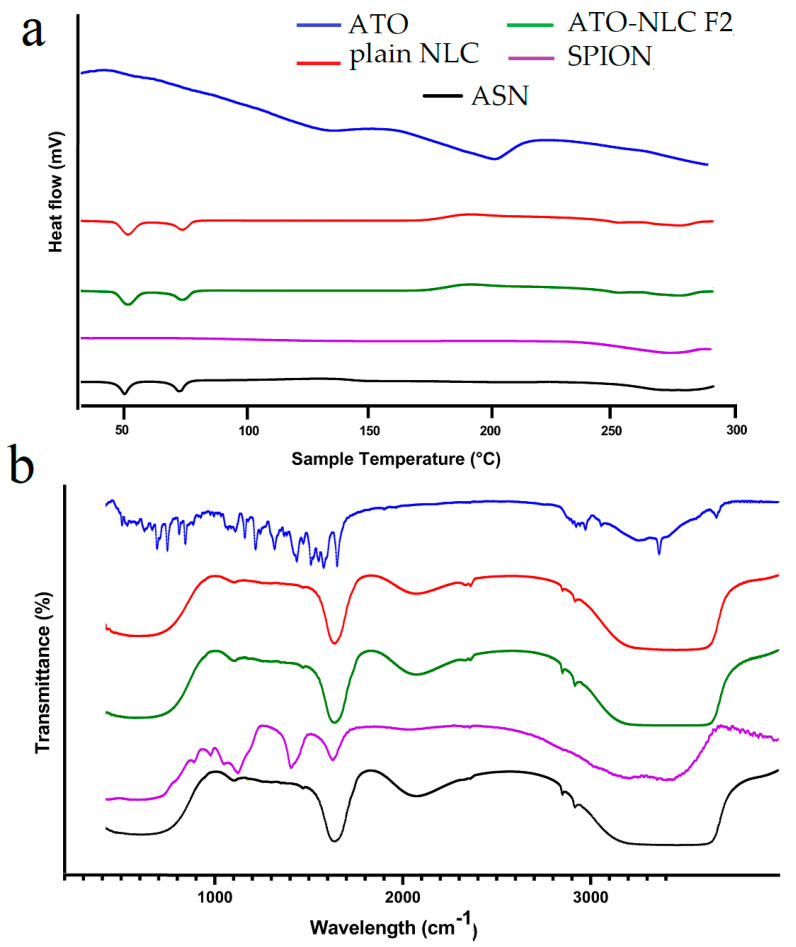
(**a**) Differential scanning calorimetry (DSC) and (**b**) Fourier-transform infrared spectroscopy of ATO, plain NLC, ATO-NLC F2, SPION, and ASN.

The DSC thermogram depicts the thermal characteristics of ATO, NLC, SPION, and ASN formulations, elucidating their crystallinity and thermal stability. The ATO curve displays extensive endothermic transitions, signifying its crystalline characteristics, whereas the NLC curve maintains relative stability with slight peaks, implying a semi-crystalline or amorphous lipid structure. The SPION thermogram exhibits negligible thermal transitions, thereby affirming its exceptional thermal stability. The ASN formulation indicates that the absence of atorvastatin’s distinct melting peaks signifies effective encapsulation within the lipid carrier, resulting in its amorphization or molecular dispersion. The incorporation of SPIONs does not induce notable phase transitions, thereby affirming its compatibility with the lipid system. In conclusion, the DSC analysis confirms that atorvastatin is effectively encapsulated in the lipid matrix, transitioning from crystalline to amorphous state, while SPIONs are well-integrated, resulting in a thermally stable and efficient drug delivery system as shown in (Figure 3a).

#### 2.2.5. Fourier-Transform Infrared Spectroscopy (FTIR)

FTIR is considered an efficient way to validate that the entrapment of drugs in lipid carriers is manifested via the disappearance of drug peaks [49]. The FTIR spectrum of ATO, plain NLC, ATO-NLC F2, SPION, and ASN are shown in (Figure 3b). Pure ATO showed a distinct peak at approximately 3666.5 cm^−1^, which indicates non-hydrogen-bonded O–H stretching. Other notable peaks were observed at 3363.7 cm^−1^ for N–H stretching, 3257.65 cm^−1^ for O–H stretching, 1651.1 cm^−1^ for C–O stretching, 1508.31 cm^−1^ for C–N stretching, and 1381.2 cm^−1^ for C–O stretching linked to the carboxyl group [50,51].

However, the ATO-NLC F2 spectrum demonstrated that the characteristic ATO peaks were absent in comparison to the pure ATO spectrum. As previously stated by Ghaname et al., this might imply molecular hydrogen bonding interactions between ATO and lipids [52]. However, the SPION spectrum reveals that surface hydroxyl groups (Fe–OH) and the adsorbed water are responsible for the weak band at 1620 cm^−1^ and also a broad band around 3350–3400 cm^−1^ [53]. The absence of the ATO and core composition peaks in the ASN spectrum confirmed NLC-SPION fabrication with efficient loading of ATO.

#### 2.2.6. Effect of Short-Term Storage

The physical and chemical instability of lipid nano-carriers, such as NLCs, along with their tendency to aggregate during prolonged storage, generally limits their application [13]. A short-term stability study was conducted on the storage of the optimum ATO-NLC F2 and ASN for a duration of 3 months [54]. The EE%, PS, ZP, and Q6h were assessed prior to and following storage at 4 ± 1° C and 25 ± 1° C. The statistical analysis revealed no significant difference (*p* > 0.05), as presented in Table 3. The formulations demonstrated no signs of precipitation, aggregation, or phase separation throughout the storage period. Our 90-day storage experiment revealed phase separation, aggregation, and no precipitation. Minimal variations in particle size (PS) and zeta potential (ZP) point to maintain structural integrity. We confirmed the retention of atorvastatin and SPION within the lipid matrix, sustained encapsulation efficiency (EE%), consistent drug release (Q6h%), displaying sustained dissolution behavior. These findings imply that under normal storage conditions, ATO-NLCs and ASN are both physically and chemically stable.

#### 2.2.7. Characterization of ATO/SPION-NLC Hydrogel

**Figure 4:**
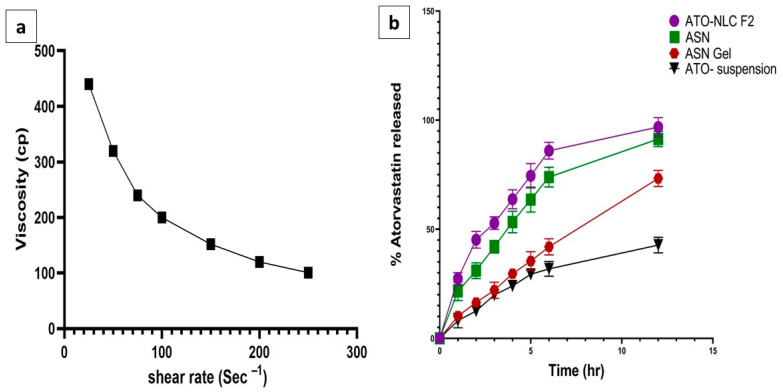
(**a**) Rheological characterization (relationship between viscosity and a shear rate of the ASN-loaded intranasal hydrogel) and (**b**) in vitro release of ATO from ATO-NLC F2, ASN-loaded intranasal hydrogel and ATO suspension, mean ± SD, *n* = 3.

The formulated ATO-loaded ASN was incorporated into the hydrogel to improve its adhesion and enhance its contact time in the nasal cavity for better drug permeation [55]. The formulated hydrogel was smooth and free of any grittiness or precipitated particles. The pH of ASN hydrogel was 5.8, which falls within the acceptable range (5–6.5) for intranasal delivery, indicating its possible non-irritant and safe application [48]. The drug content of ASN hydrogel was 95.21 ± 0.6%, which complies with the pharmacopeial acceptable range, indicating a homogeneous distribution of ATO in the gel matrix [56]. The viscosity of the ASN hydrogel was 440.4 ± 15.7 cp. As obvious in (Figure 4a), applying shear force reduced formulation viscosity, indicating a shear-thinning (pseudoplastic) behavior. These results suggested that the use of HPMC K15 created a hydrogel with high viscosity, anticipating prolonged contact time in the nasal cavity and sustaining drug release. This could boost intranasal absorption and ATO bioavailability.

#### 2.2.8. In Vitro Drug Releases Study

The release profile of ATO from ATO-NLC F2, ASN, ASN hydrogel, and ATO suspension was analyzed to anticipate the in vivo efficacy of the different formulae.

As presented in (Figure 4b), the amount of ATO released after 12 h from ATO-NLC F2, ASN, ASN hydrogel, and ATO suspension was 99.21 ± 0.71%, 93.78 ± 0.06%,74.3 ± 1.01%, and 40.23 ± 0.09, respectively.

The NLC system and ASN exhibited a biphasic release pattern, with a burst release within 1 h (21.37 ± 1.64% and 20.56 ± 2.11%) and a prolonged release thereafter.

Surface drug dissolution and NLC erosion may cause initial ATO release. The drug is likely concentrated in the outer layer surrounding the particles, complemented by the steric stabilizing action of Pluronic F68, which envelops the NLC surface and may encapsulate some ATO molecules [13].

It was previously stated by Hu et al. [57] that there is a possibility of rapid crystallization of solid lipids compared to liquid lipids during NLC preparation due to the differences in their melting points. This could result in a liquid-lipid-stripped inner core and liquid-lipid-enriched outer shell with a soft, less rigid barrier to the superficial drug release, resulting in an initial burst effect. On the other hand, the slower phase of release could be attributed to the lipid inner core, which retains the drug during the formulation process [58]. Also, the notable rise in ATO release rate could be related to the reduced particle size of the formulated nanocarrier. Decreased PS and increased total surface area improve hydration and dissolving efficiency [59].

No significant differences in the release pattern were identified in ATO-NLC and ASN, indicating a similar diffusion rate. 

Incorporating the ATO-SPION-loaded NLC system into the hydrogel resulted in a substantial reduction in the ATO release rate. This may relate to the hydrogel viscosity, which constitutes additional resistance to ATO diffusion to the release medium. This slower release profile, together with the increased nasal residence, may prolong the ATO effect and decrease the dosing frequency upon nasal administration [41]. Conversely, ATO release after suspension exhibited a reduced rate of release after 12 h, reaching 40.23% ± 0.09. This may be attributed to the crystalline form of ATO, which is known to have greater stability and reduced aqueous solubility [60].

#### 2.2.9. Mathematical Modeling of Release Profile

The in vitro release data from the optimum ATO-NLC F2, ASN, ASN–hydrogel, and ATO suspension were fitted to the different kinetic models to predict the drug release mechanism [43]. As presented in Table 4, the Higuchi diffusion was the most fitting model in illustrating the drug release from ATO-NLC F2 and ASN with a correlation coefficient value (r^2^) of 0.9935 and 0.9725, respectively. This signifies that the drug diffuses into the release medium following the erosion of the lipid matrix [20]. The results obtained align with those stated by Pradhan et al. [61]. On the other hand, ATO release from ASN hydrogel and ATO suspension followed a zero-order kinetics (r^2^ = 0.9525 and r^2^ = 0.9785). This suggests a consistent rate of ATO release and a direct relation between the time and the cumulative proportion of drugs released [62].

### 2.3. In Vitro Study of Anti-Glioma Effects of ATO-NLC F2 and ASN

#### 2.3.1. Cytotoxicity Using the MTT Assay

In vitro cytotoxicity test assessed the anticancer potential of the repurposed ATO.

**Figure 5:**
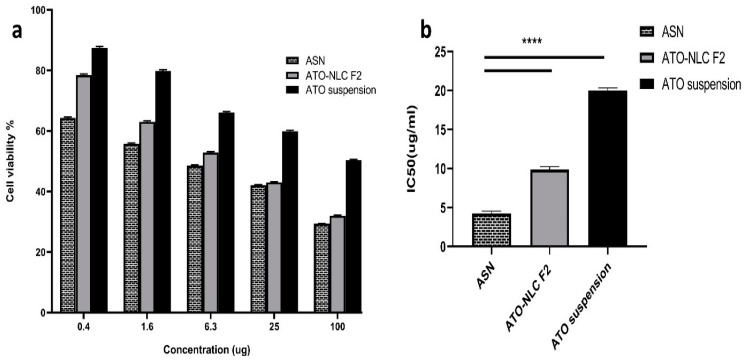
Cytotoxicity cell study: (**a**) the cell viability on T98G cancer cells of ASN, ATO-NLC F2, and ATO suspension; (**b**) IC50 of ASN, ATO-NLC F2, and ATO suspension on the T98 G cell line. Abbreviations: ATO: atorvastatin, NLCs: nanostructure lipid carriers, ASN: atorvastatin/SPION-loaded NLC. Note: ****: significant at *p* < 0.0001.

ATO suspension, the optimal formula (ATO-NLC F2), and ASN (optimum ATO-NLC combined with SPION) were tested for cytotoxicity using MTT assay on human brain adenocarcinoma (T98G) cells. Following a 24 h exposure to varying doses (0.5, 1.5, 6, 25, and 100 μg/mL) among the three evaluated treatments, the viability of the T98G cell was evaluated and represented by the growth inhibitory concentration (IC50) as depicted in (Figure 5). The findings indicate that the cell viability was diminished in a dose-dependent manner following treatment with ATO suspension, ATO-NLC F2, and ASN, as illustrated in (Figure 5a). Additionally, ASN shows a higher cytotoxic effect than the optimal ATO-NLC F2, especially at lower doses.

The half-maximal inhibitory concentration (IC_50_) values of ATO-NLC F2 and ASN were determined to be 10.057 μg/mL and 4.544 μg/mL, respectively. In contrast, the IC50 value of ATO suspension showed a significantly higher value of 20.328 µg/mL (*p* < 0.0001) rather than the other formulations, as displayed in (Figure 5b).

The optimum ATO-NLC F2 and ASN greatly increased ATO’s cytotoxicity against the T98G cell line, with IC50 values 2- to 4.5-fold lower than ATO suspension. This may be attributed to the lipid component of NLC, which promoted the drug’s intracellular uptake. Furthermore, the small size of NLCs was proven to be crucial for cellular drug uptake [63]. The small PS and large surface area of ATO-NLC F2 and ASN, together with their lipid content, may promote drug–cell interaction, enhance drug uptake by brain cancer cells, and subsequently augment their cytotoxic effect [64]. Moreover, ATO-NLC F2 was shown to exhibit a slightly lower level of cell growth inhibition compared to ASN. This could be related to the ability of SPION to traverse biological membranes and tissue. Intracellular accumulation of SPION may lead to an increase in cell tensions, which may affect mitochondrial function and result in its destruction via positive catalysis of reactive oxygen species production, ultimately impacting cell viability. This could provide an additive benefit to ATO in the ATO-SPION-NLC combination [65,66]. The results were aligned with those obtained by Tan et al. [23] in their work on zerumbone/SPION-NLC for breast cancer treatment.

#### 2.3.2. Annexin-V-FITC Analysis: (Apoptosis and Necrosis Assay)

This test was conducted to elucidate the mechanism by which ATO, either free or loaded into NLC, inhibits cell growth by measuring their apoptotic and necrotic effect.

**Figure 6:**
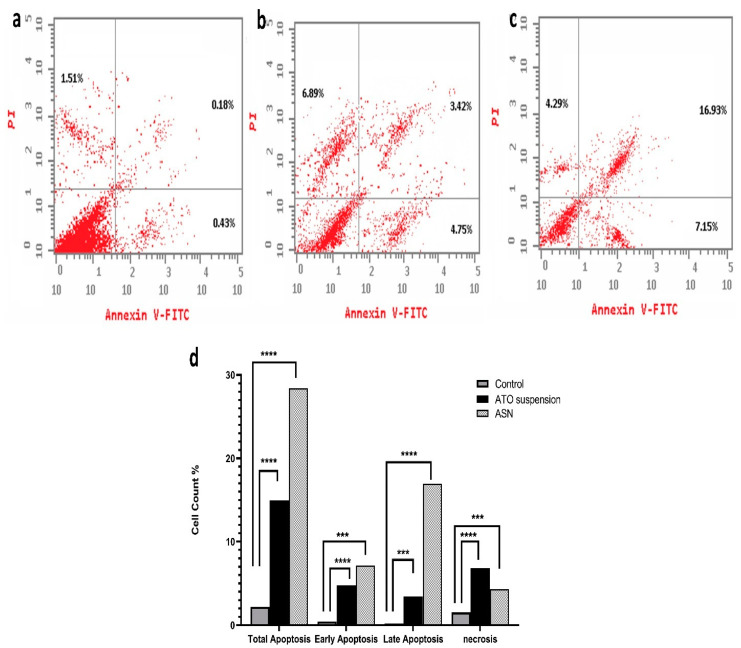
Assay of apoptosis and necrosis for (**a**) control, (**b**) ATO suspension, (**c**) ASN, and (**d**) percentage of cell count analysis. Abbreviations: ATO: atorvastatin, ASN: atorvastatin/SPION-loaded NLC. Note: *p*-value **** means <0.0001, *** means 0.0003 relative to early apoptosis, and *** means 0.0002 in late apoptosis and necrosis.

The results shown in (Figure 6) demonstrate significant differences in apoptosis induction by the tested formulations. Comparing the ASN formulation (28.37 ± 0.6%) to the ATO suspension formulation (15.06 ± 0.7%) and the control group (2.12 ± 0.2%), the ASN formulation showed a higher total apoptosis percent. Although ATO suspension caused lower total apoptosis than ASN, it remained notably greater (*p* < 0.0001) than the control group. The distribution of apoptosis showed a significant difference (*p* = 0.0049) between the early (4.75 ± 0.05%) and late phases of ATO suspension (3.42 ± 0.15%). Conversely, ASN demonstrated a markedly elevated total apoptosis percentage (*p* = 0.0002), with a significant impact on early apoptosis (7.15 ± 0.32%) and late apoptosis (16.93 ± 0.11%) (*p* = 0.0003). This implies its capacity to efficiently initiate apoptotic pathways in T98G cells. The evaluation of necrosis indicated significant variations among the formulations. Necrosis rates were significantly higher in the ATO suspension (6.8 ± 0.15%) (*p* = 0.0015) compared to the control group (1.51 ± 0.21) and optimum ASN formulation (4.29 ± 0.2%) (*p* = 0.0016). This suggests that ATO’s cytotoxic action was connected with its capacity to cause cellular damage and compromised membrane integrity. Loading ATO into the optimal ASN formulation slightly lowered its necrotic effect, suggesting that ATO-loaded ASN may exert its cytotoxic effects primarily through apoptotic mechanisms rather than necrosis. An investigation of the cell cycle was conducted to better understand the mechanism behind the cytotoxic effects of ASN.

#### 2.3.3. Study of the Cell Cycle

**Figure 7:**
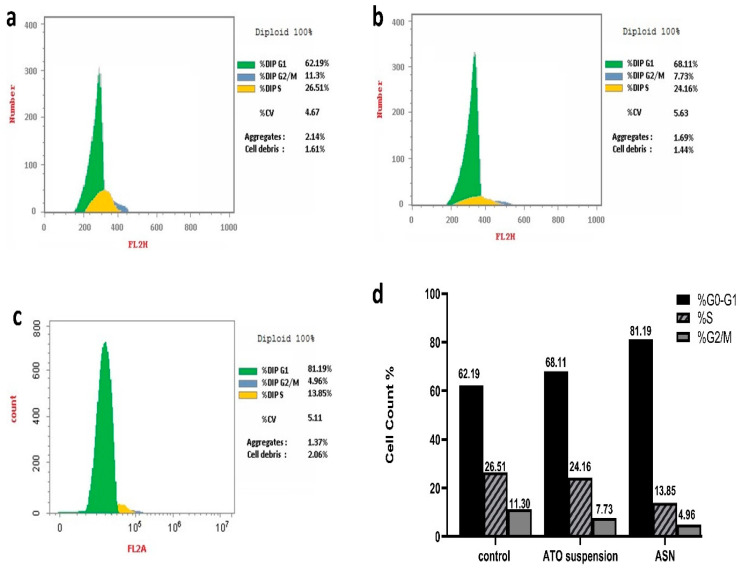
Analysis of cell cycle for the (**a**) T98G untreated cells, (**b**) T98G cells received the ATO suspension, (**c**) T98G cells received the ASN, and (**d**) a percentage of the cell cycle.

DNA replication happens during cellular division, nuclear division, and cytoplasmic partitioning follow. DNA replication helps to enable a fundamental process of cellular reproduction called the cell cycle [12]. Using nuclear PI labeling and flow cytometry, (Figure 7a–d) shows the T98G cell cycle behavior of ASN and ATO suspension. Notably, when T98G cells were exposed to ASN and ATO suspension, there was a significant increase in the percentage of cells in the phase of G0-G1, with respective 1.3- and 1.1-fold increments compared to the control. Specifically, the proportions of cells aggregating in this phase were 81.19% and 68.11% for ASN and ATO suspension, respectively, compared to 62.19% with the control T98G cells.

On the other hand, the concentration of cells in the G2/M and S phases decreased in comparison to the control group as a result of the treatment with ATO suspension and ASN.

The percentage of cells in phase G2/M showed a respective 2.3- and 1.5-fold reduction upon treatment with ASN and ATO suspension. At the same time, the cells in the S phase, upon being treated with ASN and ATO suspension, were reduced by 1.9 and 1.1 folds, respectively, to the control group. Consequently, the phase of G0–G1 of brain cancer cell growth could be the main time when the ideal mixture inhibits cancer cells. This was significant because, although DNA synthesis occurs within the S phase, the G0/G1 phase was necessary to prime the cells for the replication and division of DNA [40]. Previous investigation indicates that mevalonate deprivation results in G0-G1 phase cell cycle arrest in breast cancer MCF-7 cells. This phenomenon is partially attributed to the reduced activity of cyclin-dependent kinase 2 (CDK2) and the diminished expression of positive regulators of the cell cycle progression from the G1 phase to the S phase [67]. A further investigation revealed that administering ATO to ovarian cancer cells resulted in G1 phase arrest and a dose-dependent increase in annexin V expression while cleaved Poly (ADP-ribose) polymerase (PARP). This is consistent with the suggested mechanisms of action of ATO [68].

#### 2.3.4. Assessment of Inflammatory Biomarkers

**Figure 8:**
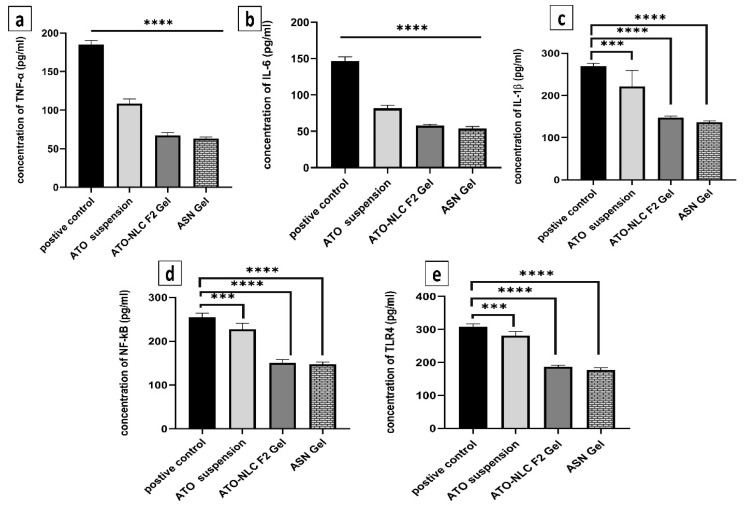
The anti-inflammatory effects of various experimental groups (control, ATO oral suspension, ATO-NLC F2 IN hydrogel, and ASN IN hydrogel) on LPS-induced brain inflammation as observed by the biomarkers (**a**) TNF-α, (**b**) IL-1β, (**c**) IL-6, (**d**) TLR4, and (**e**) NF-kB. Note: *p*-value **** <0.0001, and *** <0.001.

Inflammatory biomarkers were evaluated to determine the mechanism by which ATO/SPION-loaded NLC (ASN) intranasal hydrogel efficiently cures GBM by reducing the NF-κB/IL-1β/IL-6/TNF-α/TLR4 signaling pathways. The inflammatory microenvironment is known to promote malignancy, particularly in glioblastoma. Pro-inflammatory cytokines, growth factors, and chemokines envelop glioblastoma cells and enhance their proliferation [15]. Figure 8 depicts how ASN, ATO-NLC F2, and ATO suspension affect serum inflammatory cytokine protein profiles. LPS was used to generate inflammatory cytokines as a positive control. The biomarker profiles were measured after ASN, ATO-NLC F2, and ATO suspension treatment. The ASN administration markedly decreased (*p* < 0.05) the synthesis of TNF-α, IL-6, IL-1β, NF-κB, and TLR4 by 34.7%, 38.1%, 51.08%, 52.4%, and 54.48%, respectively. A comparable inflammatory profile was seen with ATO-NLC F2 Gel treatment. The progression, invasion, and angiogenesis of glioblastoma are contingent upon interleukins and cytokines. Interleukin-1β (IL-1β) is a dominant pro-inflammatory cytokine found in the glioblastoma microenvironment and regulates its growth. The binding of IL-1β to its receptor (IL-1R) promotes MAPK and NF-kB signaling pathways, leading to increased gene expression via multiple target genes. Thus, specific suppression of IL-1β production and/or activity may be an effective approach to limit GBM growth [15].

On the other hand, oncogenic mutations, along with other external or internal triggers, may cause cancer cells to secrete IL-6. IL-1β and TNF-α activate signaling pathways that lead to the stabilization of IL-6 mRNA and its subsequent production [69]. NF-κB controls genes that contribute to biological functions such as proliferation of cells, cell differentiation, motility, and survival. Abnormal NF-κB activation is prevalent across various cancer types, including glioblastoma. NF-κB signaling activation promotes GBM mesenchymal growth by modulating downstream transcriptional pathways [70]. Compared to the control group, ASN-treated cells displayed lower NF-κB and TLR4 levels, indicating that ATO-loaded nanoparticles effectively reduced LPS-induced inflammation. ATO suspension showed comparable outcomes although at a decreased degree. The results show that NLC formulation synergistically improved the anti-inflammatory action of ATO, thereby maybe increasing its antiproliferative efficacy in GBM. The results show that a brain-targeting approach for the treatment of GBM may be ATO-SPION-loaded NLC intranasal hydrogel.

### 2.4. In Vivo Studies

#### 2.4.1. In Vivo Pharmacokinetics Study

**Figure 9:**
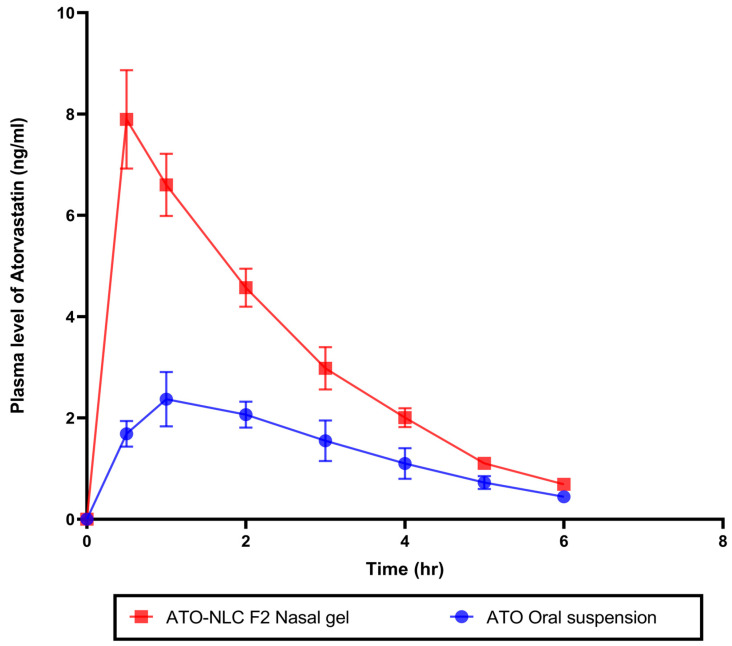
In vivo study of plasma concentration–time curve for optimal atorvastatin-loaded nanostructure lipid carrier gel (ATO-NLC F2) administrated intranasally and atorvastatin oral suspension.

An in vivo pharmacokinetics study assessed ATO bioavailability following intranasal therapy with the optimum ATO-NLC F2 hydrogel. Plasma samples from rabbits in three groups were taken at specified times following IN of ATO-NLC F2 hydrogel, oral administration of ATO suspension, or 0.9% saline solution. ATO calibration curve in plasma was constructed at a concentration range of 0.5 to 50 ng/mL with R^2^ values of 0.9999. The amount of ATO in plasma samples was measured and calculated, as shown in (Figure 9). Non-compartmental pharmacokinetic analysis was employed to estimate the pharmacokinetic parameters of ATO (Table 5). The IN ATO-NLC F2 hydrogel showed a significantly higher C_max_ (7.89 ng/mL) compared to ATO oral suspension (2.37 ng/mL) (*p* < 0.0001). Also, the calculated AUC_0→6_ was significantly higher (*p* < 0.0001) in the intranasally administered ATO-NLC F2 gel than in oral suspension, where the obtained value was 17.92 ng·h/mL and 7.86 ng·h/mL, respectively, with a 2.28-fold enhancement in the bioavailability. The improved bioavailability of ATO in the ATO-NLC F2 intranasal hydrogel could be related to different factors, such as the lipophilic and elastic properties of NLC, which facilitate the partitioning of particles into nasal epithelial cells and enable direct passage through the cells, resulting in enhanced systemic absorption [18]. Intranasal ATO is conveyed by the olfactory nerves, either extracellularly or intracellularly, in the upper nasal cavity to the central nervous system (CNS) [71]. The extracellular process involves the increased movement of ATO molecules between nasal epithelial cells. This method improved the transport of ATO to the olfactory bulbs and the central nervous system within minutes after the intranasal administration of the optimized ATO formulation. Additionally, the intracellular mechanism encompasses the endocytosis mechanisms or passive diffusion of ATO molecules within the olfactory receptor neuron, which is followed by the slower (within several hours) axonal transport of ATO to the olfactory bulbs and other brain regions. Also, it is reported that a portion of the trigeminal nerve terminates in the olfactory bulbs [27]. Consequently, ATO delivered intranasally from the optimum formulation may access the olfactory bulb and other rostral brain regions via trigeminal pathways. Including HPMC in the nasal gel results in a favorable mucoadhesive characteristic, effectively extending the duration of the drug’s presence in the nasal cavity and leading to an increase in bioavailability [55]. It has been demonstrated that nanostructured lipid carriers (NLCs) serve as excellent nanocarriers for targeting ATO in the brain.

#### 2.4.2. Brain Imaging by Confocal Laser Scanning Microscopy (CLSM) for Drug Distribution

CLSM was utilized to examine the diffusion of nanoformulation into the brain.

**Figure 10:**
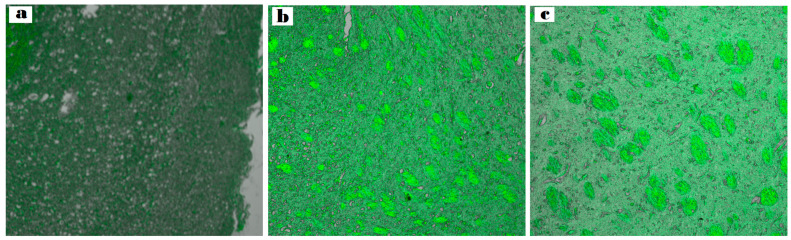
Comparative CLSM pictures illustrate the depth of (**a**) RhB–oral solution, (**b**) RhB-loaded ATO-NLC F2 intranasal hydrogel, and (**c**) RhB-loaded ASN intranasal hydrogel.

The ASN hydrogel was reprepared using a fluorescent dye (RhB) instead of the drug. After intranasal administration, the rabbit’s brain was separated, and rabbit brain cryosections were imaged to verify the formulation’s capacity to target the tissues of the brain.

(Figure 10a–c) illustrates the depth of penetration quantified along the Z-axis by fluorescence detection. As observed at a depth of 33.5 μm, the brain sections of the rabbits treated with intranasal RhB-ASN hydrogel exhibited the highest fluorescent intensity (127.731 ± 0.23) followed by those treated with intranasal RhB-ATO-NLC F2 hydrogel (106.512 ± 1.09) in contrast to the low fluorescence in the brain tissues of those treated with RhB solution orally (79.952 ± 1.67). Significant differences between the three groups were identified through statistical analysis (*p* < 0.05), demonstrating a significant increment in dye distribution to the brain tissues upon the formulation of NLC systems and a further increase upon the inclusion of SPION into the formulation. NLCs provide advantageous physicochemical characteristics and biocompatibility, enhancing the drug distribution to cells of the brain. The surfactants in the NLC formulation tend to enhance the fluidity, permeability, and bioavailability of ATO-NLC [72]. They also facilitate the transporter-mediated mechanisms that help with drug absorption. In addition, the small size of the nanoparticles enhances their capacity to traverse the narrow openings in the olfactory mucosa tissue and the BBB tight junctions, despite potential repulsion with the negatively charged NLC and the negative of the nasal mucosa [73]. Furthermore, the lipid properties of the carrier improved the drug’s solubility and permeability, enabling its direct passage through the nasal olfactory region while also offering protection against metabolic enzymes located in the nasal mucosal canal and the epithelial cells that line it [74].

Furthermore, as (Figure 10c) shows, adding SPION enhanced brain-targeting behavior. An external magnetic field navigated the nanoparticles toward the intended tissue quite effectively. These results indicate that the optimal ASN is capable of transporting medications directly from the nostril to the brain with minimal dissemination to other body organs. Preventing drug distribution to other tissues, bypassing the first-pass metabolism, and enhancing the drug bioavailability while decreasing the off-target (side) might provide significant advantages in treating brain cancer glioma. These findings indicate that administering the tailored ATO/SPION-loaded NLC via the intranasal route may provide greater therapeutic benefits compared to utilizing an oral ATO suspension. This approach can be suggested as an alternative, non-invasive, and efficient method for treating brain tumors.

#### 2.4.3. Histopathological Study 

**Figure 11:**
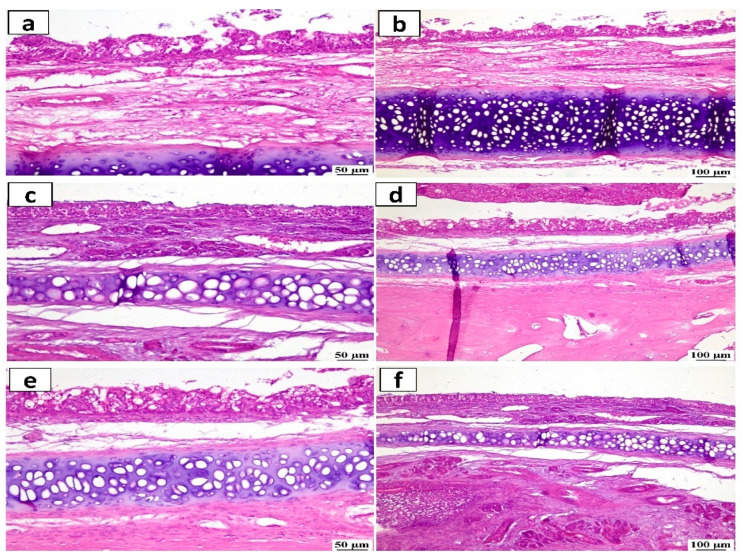
Histopathological study of nasal mucosa (hematoxylin and eosin stained) from (**a**,**b**) the control group (untreated), (**c**,**d**) the group received the ATO-NLC F2 intranasal hydrogel, and (**e**,**f**) the group received the ASN intranasal hydrogel with a magnification scale of 50 μm (left side) and magnification scale of 100 μm (right side).

The histopathology analysis was used to evaluate the safety and potential irritant effect following the IN administration of ATO-NLC F2 and ASN hydrogel formulation. (Figure 11) depicts stained photomicrographs of the anterior cross-section of the nasal cavity of rabbits exposed to both systems. The examination of both the control and treated mucosa revealed a relatively normal histological structure with no evidence of irritation, subepithelial edema, or vascular congestion. Furthermore, none of the rabbits had any severe symptoms, including shedding of epithelial cells, bleeding, or the development of necrotic tissues.

### 2.5. In Silico Studies

#### ATO-Loaded Nanostructured Lipid Carrier (NLC) Formation and ADME Study

**Figure 12:**
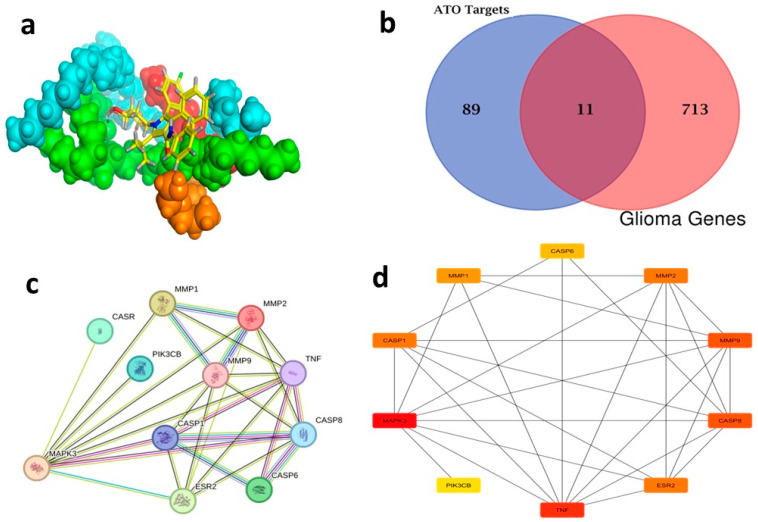
(**a**) Three-dimensional representation of ATO–Compritol complex, (**b**) Venn diagram of ATO target and disease target, (**c**) PPI network of glioma common targets, and (**d**) Top 10 genes in the PPI network of malignant glioma-related targets of ATO.

In silico studies were applied to evaluate the chemical compatibility between the optimum NLC ingredients (Caproyl 90, Lecithin, Pluronic f-68, and Glyceryl 1,2-dibehenate (Compritol)) and ATO and predict the final complex structure of the optimum ATO-NLCs. MOE software (MOE 2019.0102) was used to add protons and minimize energy, followed by molecular dynamic (MD) simulation. Interestingly, the results showed that the ability of NLC ingredients to enclose ATO, as depicted in the higher EE% of the optimum formula, could be due to hydrophobic Vanderwaal interaction forces and a single hydrogen bond between Compritol and ATO (Figure 12a). Comparing the ADME properties of ATO to those of Compritol, the main lipid ingredient of the optimum NLC, revealed that ATO-loaded NLC would offer an enhanced in vivo therapeutic effect. As presented in Table 6, Compritol-based NLCs would escape the effect of multidrug resistance protein-1 (Pgp) (a transmembrane unidirectional efflux pump) located at the luminal side of the BBB and in the olfactory system [75], which may subsequently enhance ATO absorption. Moreover, ATO-loaded Compritol-based NLC could delay the metabolism of ATO by abolishing its suitability to CYP3A4 enzyme. Finally, ATO-loaded NLC would have a lower clearance level, prolonging its bioresidence time and enhancing its in vivo activity (Table 6).

### 2.6. Network Pharmacology

#### 2.6.1. Candidate Ingredient Screening

ATO targets were analyzed using the Swiss Target server, resulting in the identification of 100 targets. Following the merging of 724 malignant glioma targets obtained from the DisGeNET database using the Venn diagram intersection feature of FunRich 3.1.3 software, 11 overlapping targets were identified as potential targets, as seen in (Figure 12b) (Table S4).

#### 2.6.2. Construction and Analysis of the PPI Network

By selecting the “human” species from the String database, a network graph with 11 vertices and 29 edges was generated (Figure 12c). The cytoHubba plug-in (Cytoscape 3.10.1) and the TSV file exported from this website were employed to identify the top ten genes, which included MAPK3, TNF, CASP8, MMP9, ESR2, CASP1, MMP2, MMP1, CASP6 and PIK3CB (Figure 12d) (Table S5). These top 10 genes, particularly MAPK3 (ERK1) within the PPI network of malignant glioma-related targets of ATO, are interconnected and work collaboratively to suppress the progression of malignant glioma. The treatment of malignant glioma is significantly influenced by the core genes that are regulated by the network [76].

#### 2.6.3. The KEGG Pathway Assessment of Prospective Targets

**Figure 13:**
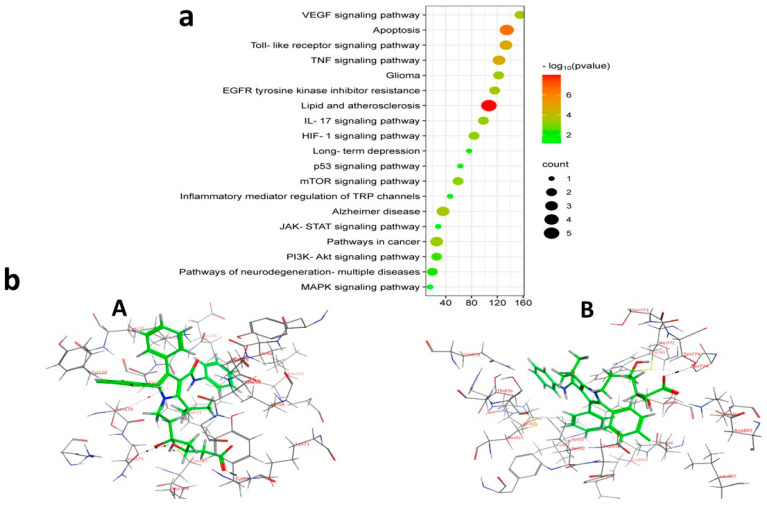
(**a**) KEGG pathway enrichment study of results for malignant glioma treatment using ATO (**b**) 3D representation of ATO against target enzymes: (**A**) MAPK3 and (**B**) PI3K.

KEGG enrichment assessment of the 11 prospective targets was performed through Funrich and ShinyGO 0.8. Based on *p*-value ranking (*p* < 0.05), the top 19 pathways were determined, and a bubble diagram was displayed in (Figure 13a) (Table S6). The study identifies several signaling pathways associated with malignant gliomas. Among them are the PI3K-Akt signal pathway, apoptosis, EGFR inhibitor resistance, glioma, pathways in cancer, and the neuro-inflammatory toll-like receptor signaling pathway. As shown in supplementary figures (Figures S1–S6) downloaded from the KEGG database and PPI target gene ranking results, MAPK3 and PI3K (PIK3CB) were shown as common targets among all malignant glioma pathways and the neuroinflammatory toll-like receptor signaling pathway. It appears to be involved in controlling the G0/G1 phase pro-survival Bcl2 (Apoptosis), cell proliferation, growth and angiogenesis (EGFR inhibitor resistance pathway, PI3K-Akt signal pathway, pathways in cancer and glioma), and finally, inflammatory cytokines such as TNF-α, Il-1β, and IL-6 in addition to TLR4 neuroinflammatory receptor (toll-like receptor signaling pathway). Moreover, PI3K (PIK3CB) that manages the PI3K signaling pathway also revealed a synergistically regulated crossing talk between NF-ĸB and P53 pathways. These results indicated that ATO might play an anticancer role by regulating this group of signaling pathways via the intrinsic co-leaders MAPK3 and PI3K (PIK3CB).

#### 2.6.4. Molecular Docking

To further investigate and verify the pattern of action of ATO for GBM treatment and associated neuroinflammation, the core targets, MAPK3 and PI3K, identified by the PPI network and KEGG pathways, were chosen for docking simulation studies against ATO using MOE software. The findings indicated that ATO demonstrated interactions with the core targets (Table 7). For MAPK3, ATO possessed a promising binding score equal to −7.8959 kcal/mol through binding interactions, including hydrogen bonding, and ionic and hydrophobic interactions with key amino acid residues Cys138, Lys71, Asn171, and Ser170 (Table 7) (Figure 13(bA)). On the other hand, ATO revealed a good binding interaction score with PI3K (−7.0106 kcal/mol) with various binding forces, such as H-Bonding, ionic and lipophilic interactions with the key residues Ser774, Lys802, and Ile932 (Table 7) (Figure 13(bB)). Briefly, the in silico molecular docking study revealed the promising effect of ATO against two important Glioma pathways, namely, PI3K and MAPK, with high binding affinity that was demonstrated via the binding interactions of the key residues of both the targets and the higher values of the binding score.

## 3. Materials and Methods

### 3.1. Materials

We used Labrasol (Caprylocaproyl macrogol-8 glycerides), Compritol 888 ATO (glycerol dibehenate), Capryol 90 (propylene glycol monocaprylate (type II), Labrafil M 1944 CS (oleoyl polyoxyl-6 glycerides), gratefully donated by Gattefosse (St Priest Cedex, France). Atorvastatin was kindly provided by the Al-Arabiya Pharmaceutical Company (Cairo, Egypt). Pluronic F68 (polyoxyethylene-polypropylene (150:29) block copolymer, poloxamer 188), HPMC K15M (hydroxypropyl methylcellulose), and cellulose membrane (cut off 12 kDa) were obtained from Sigma Aldrich, Inc. (St. Louis, MO, USA). Lecithin (70% phosphatidylcholine) was gifted by Lipoid (Ludwigshafen, Germany). Tween 80 (polyoxyethylene-80-sorbitan monooleate, polysorbate 80), Ferrous sulfate tetrahydrate, and ferric chloride hexahydrate were purchased from ADWIC Chemicals (Cairo, Egypt). All reagents were of analytical quality and required no additional purification.

### 3.2. Optimization Design

A three-level, two-factor 3^2^ full factorial design was developed to evaluate the impact of formulation variables such as the type of liquid lipid (X_1_; Capryol, Labrafil, or Labrasol) as well as surfactant type (X_2_; Tween 80, Pluronic F68, or a 1:1 mixture) on ATO-NLC characteristics namely entrapment efficacy (EE,%, Y_1_), particle size (PS, nm, Y_2_), zeta potential (ZP, mV, Y_3_), and ATO released after 6 h (Q6h,%, Y_4_), as depicted in Table 8. The Design Expert^®^ software version 13 (Stat Ease, Inc., Minneapolis, MN, USA) produced nine experimental trials to be conducted in duplicates, as shown in (Table 8). Analysis of Variance (ANOVA) with a *p*-value of 0.05 was used to evaluate the significance of each formulation factor on the dependent variables. The optimal model, whether the main effects or two-factor interaction models, was used to statistically analyze the observed responses. Ultimately, the software enhanced the results and determined the optimal formulation parameters for the best formula.

### 3.3. Preparation of Atorvastatin-Nanostructured Lipid Carriers (ATO-NLCs)

ATO-NLCs were formulated via emulsification with a high-speed homogenizer followed by ultrasonication. First, ATO (10 mg) was mixed with the liquid lipid (Capryol, Labrafil, or Labrasol; 0.5% *w*/*w* (50 mg)) followed by the addition of 100 mg of Compritol 888 ATO (1% *w*/*w*) as the solid lipid, and 50 mg lecithin (0.5% *w*/*w*) as a hydrophobic emulsifier. Simultaneously, the hydrophilic emulsifier 100 mg (Tween 80, Pluronic F68, or their mixture) was introduced to 10 mL of double-distilled water. Each phase was heated separately at 75 °C for 15 min. The aqueous phase was then introduced to the lipid phase drop by drop. The ingredients were blended for 10 min at 10,000 rpm at the same temperature using an Ika T10 basic homogenizer (Ultra-Terrax, IKA-Werke GmbH & Co. KG, Staufen, Germany). Finally, the emulsion was sonicated using a probe sonicator “Sonics Vibra-Cell VCX130, (Sonics & Materials, Inc., Newtown, CT, USA)” for 10 min. Then, the dispersion was cooled at room temperature [13].

### 3.4. Characterization of ATO-NLCs

#### 3.4.1. Measurement of EE%

Utilizing a cooling ultracentrifuge (Sigma 3–30 KS, Roedermark, Germany), 1 mL of the different formulae was centrifuged for 2 h at 4 °C and 20,000 rpm. The supernatant was discarded, and the sedimented residue was dissolved in methyl alcohol to disturb the formulated nanosystems and release the encapsulated drug. Finally, the amount of ATO was quantified using a UV spectrophotometer (Shimadzu UV1650 Spectrophotometer, Koyoto, Japan) at ƛ max 246 nm [12]. The EE% was measured using the following equation:

(1) $$\mathrm{EE}\%=\frac{Total\;amount\;of\;ATO-Total\;amount\;of\;free\;ATO}{Total\;amount\;of\;ATO}\times 100$$

EE% is the encapsulation efficiency %, AA is the amount of ATO entrapped in NLCs, and TA is the total amount of ATO in each formula.

#### 3.4.2. Measurement of PS, ZP, and PDI

Zetasizer NanoZS (Zetasizer Nano Series, Malvern, UK) was used to evaluate PS, PDI, and ZP at 25 °C after 100-fold dilution in deionized water for each developed ATO-NLC formulation [77].

#### 3.4.3. In Vitro Drug Release Study

The ATO-NLC release was investigated using a USP dissolution apparatus device type II (Pharma Test, Hainburg, Germany). A 100 mL solution of phosphate-buffer saline (PBS, pH 6.4) was stirred at a rate of 100 rpm while the temperature stayed at 37 ± 0.5° C. Trimming the needle end turned a 5 mL syringe into a tube. The syringe was filled with 2 mg of ATO-equivalent NLCs from the top after removing the pump. A 12 kDa pre-soaked cellulose membrane was placed atop the syringe and connected to the rotating paddle [55]. Three mL samples of the dissolution medium were withdrawn at specified points of time (1, 2, 3, 4, 5, and 6 h) and substituted with a new medium. The quantity of ATO released at each time point was quantified spectrophotometrically at a wavelength of 246 nm, and the percentage released was calculated. The percentage of ATO released after 6 h (Q6h) was used for the evaluation of various formulations.

#### 3.4.4. Optimization of ATO-NLC

The optimal ATO-NLC formula was selected using Design Expert software, according to the desirability function and constraints on increasing EE%, Q6h%, ZP, and decreasing PS. The ATO-NLC formula with the greatest desirability function (approaching 1) was chosen for further characterization [21].

### 3.5. Superparamagnetic Iron Oxide Nanoparticle (SPION) Preparation

The SPION was prepared utilizing the co-precipitation technique outlined by Abbas et al. in a prior study [24]. In brief, 1.17 g of ferric chloride hexahydrate and 0.6 g of ferrous sulphate tetrahydrate (molar ratio 1.75:1) were mixed well in 50 mL of deionized water for one hour at 70 °C in an N2 atmosphere. After that, the solution of ammonium hydroxide (32%) was introduced to the entire mixture while stirring for a further hour. The final dispersion was subsequently permitted to cool at the ambient temperature, and a magnet was used to separate magnetite (Fe_3_O_4_). Finally, the SPION was washed thoroughly with warm water five times and dried in the oven overnight, maintained at 50 °C.

### 3.6. ATO/SPION-Loaded NLC Preparation

The fabrication of ATO/SPION-loaded NLC (ASN) was conducted utilizing a method previously outlined [23]. ATO-NLC-SPION were synthesized utilizing a standardized procedure including the dispersion of 20 mg of SPION in 500 μL of ethanol. The mixture was then ultrasonicated at 37 kHz for 30 min at room temperature. Subsequently, the resulting solution was mixed with the optimum lipid matrices, and ATO/SPION-loaded NLC (ASN) was prepared as previously described.

### 3.7. Characterization of ASN

#### 3.7.1. Assessment of the PZ, EE%, ZP, and Q6h

The amount of ATO entrapped within ASN, the size of the particle, zeta potential, and the amount of ATO released after 6 h were measured as mentioned before under section the in vitro drug release study.

#### 3.7.2. Assessment of Magnetic Behavior

Using a vibrating sample magnetometer (Lake Shore Model, Lake Shore Cryotronics, Westerville, OH, USA), the magnetism of SPION and ASN was determined at room temperature. The samples were affixed to a sample holder, and a magnetic field ranging from −2 to 2 kG was applied for this investigation [23].

#### 3.7.3. Morphology Study

The morphology and surface topography of the optimum formula (ATO-NLC F2), SPION, and ASN were visualized using transmission electron microscopy (TEM) after negative staining. A single drop of the aqueous dispersion was applied to a carbon-coated copper grid for one minute and allowed to adhere. The surplus liquid was removed using filter paper. After staining with 1% phosphotungstic acid solution, the sample was desiccated and assessed using TEM (JEM 1230, Joel, Tokyo, Japan) at 100 kV [21]. Furthermore, The SPION’s morphology was evaluated using a scanning electron microscope (JSM-6360, JEOL, Tokyo, Japan). The samples were gold-coated before the examination. A double-sided adhesive strip was employed to adhere approximately 1 mg of the sample to a receptacle. SEM pictures were obtained at an accelerating voltage of 15 kV [23].

#### 3.7.4. Differential Scanning Calorimetry (DSC)

The DSC thermogram depicts the thermal characteristics of ATO, NLC, SPION, and ASN formulations, elucidating their crystallinity and thermal stability using a DSC-60 (Shimadzu Corporation, Kyoto, Japan) with indium (m.p = 156.6 °C, purity = 99.99%) at a 10 °C/min heating rate [78].

#### 3.7.5. Fourier-Transform Infrared (FTIR) Spectroscopy

Pure ATO, blank NLC, the optimum formula (ATO-NLC F2), SPION, and ASN were evaluated using FTIR (Jasco, FT/IR 6100,Tokyo, Japan). Two milligrams of each sample were mixed with dry KBr. The mixture was then compressed into a disk under pressure at room temperature. Subsequently, the disk underwent scanning within the 400–4000 cm^−1^ wavelength range [62].

#### 3.7.6. Short-Term Storage Study

The physical stability of ATO-NLC F2 was investigated in terms of drug leakage, crystal growth, and physical alterations after storage. The sample was stored in glass vials for 90 days at 25 ± 1 °C and 4 ± 1 °C. After that, the samples were visually examined for any sedimentation or color changes. Furthermore, the EE%, PS, ZP, and Q6h were re-evaluated and compared with the newly generated samples [34]. The measures were performed three times, and the findings were analyzed using a t-test with a significance threshold of *p* < 0.05 (GraphPad Prism, Version 9, San Diego, CA, USA) [79].

### 3.8. Preparation of ASN Hydrogel

ASN was incorporated in a hydrogel matrix to improve ATO nasal adhesion to the nasal mucosa. HPMC K15M (2%, *w*/*w*) was gradually added in half of the required volume of double-distilled water at 70 °C with constant stirring until a gelling consistency was achieved. The prepared gel was chilled overnight to obtain a transparent gel devoid of air bubbles. Ultimately, ASN was included, and the gel volume was modified using deionized water [21].

### 3.9. Characterization of ASN Intranasal Hydrogel

The appearance, homogeneity, and clarity of the freshly formulated intranasal hydrogel were assessed. The pH of the ASN hydrogel was evaluated in triplicate after dilution (10-fold) using a digital pH meter (InolabpH720, WTW, Weilheim, Germany) [80].

The drug content of the hydrogel was also assessed, where one gram of the gel was solubilized in methyl alcohol (100 mL). Following filtration via 0.45 µm filter membrane, the solution was evaluated at λmax 246 nm by spectrophotometer [80].

(2) $$\mathrm{Drug}\;\mathrm{content}\%=\frac{actual\;amount\;(1\;g\;gel)}{theoretical\;amount\;(1\;g\;gel)}\times 100$$

The rheological behavior of ASN hydrogel was investigated at a temperature of 25 ± 1 °C utilizing a cone and plate viscometer (Brookfield programmable DVII + Model pro II type, Brookfield, IL, USA) equipped with a 52-spindle number at 50 rpm, and measurements were recorded in triplicates [55].

#### 3.9.1. In Vitro Release Study of ASN Intranasal Hydrogel

ATO was released from the loaded gel using USP dissolution tester equipment II, as mentioned previously under the section of in vitro drug release study.

#### 3.9.2. Mathematical Modeling of ATO In Vitro Release

The release data were fit into different mathematical models (zero, first, second-order, Higuchi diffusion, and Baker–Lonsdale) using the DD Solver programmer (Excel Add-in) to study the release kinetics. The correlation coefficients (r^2^) were calculated for each model, and the model with the highest r^2^ was selected as the best-fitting model [62].

### 3.10. In Vitro Study of Anti-Glioma Effects of ASN

#### 3.10.1. Culture and Cell Lines

The formulations were evaluated on the glioblastoma human cell line T98G from the American Type Culture Collection (ATCC) via VACSERA Egypt to test these formulations on the brain tissue. T98G cell lines were cultured in Dulbecco’s Modified Eagle Medium (Invitrogen/Life Technologies) with 10% FBS, (100 μg/mL) streptomycin/penicillin, HEPES (10 mM), and glutamine (2 mM) at 37 °C with 7.4 pH and 5% CO_2_ humidity. During cell passage, 90% of the medium was substituted daily with new media [81]. The cultured cells were treated with ATO suspension, ATO-NLC-F2, and ASN.

#### 3.10.2. In Vitro Cytotoxicity Using the MTT Assay

Using an in vitro toxicology assay kit (Sigma, M-5655), the MTT test was used to determine the in vitro cytotoxicity of ATO suspension, ATO-NLC-F2, and ASN on T98G cell lines as previously stated [12]. On a 96-well plate, T98G brain cancer cells that had undergone a brief treatment were planted at 2 × 10^4^ cells/mL density. For 48 h, several doses of ATO suspension, ATO-NLC F2, and ASN—equivalent to 0.4 to 100 μg/mL—were incubated with the cancer cells. Each well thereafter received MTT solution (0.5 mg/mL). After incubating for 4 h, the formazan crystals were dissolved with 150 μL of DMSO. Optical density was measured with a spectrophotometer at a wavelength of 570 nm. The experiment was conducted in triplicate. The IC_50_ value, representing the concentration that inhibits cell division by 50%, was determined to assess the efficacy of the tested formulation in suppressing cell proliferation.

#### 3.10.3. Annexin-V-FITC Analysis: (Apoptosis Assay)

The processes of apoptosis were evaluated utilizing the Annexin V-FITC Apoptosis Detection Kit (Mountain View, CA, USA) on T98G cells, as previously reported [81]. The T98G cells were cultivated in culture flasks with a 25 cm^3^ surface area and 1 × 10^6^ cells/mL density for 48 h. After that, cells were treated with ATO suspension and ASN at its IC_50_ concentration. Trypsin-collected cells were centrifuged for five minutes at 1000 rpm. The cell pellets were washed using ice-cold PBS (pH = 7). After that, the cells were resuspended at a 1 × 10^6^ cells/mL density in a binding buffer. Annexin V-FITC (5 μL)/PI solution (10 μL) was added and incubated for thirty minutes in the dark at 37 °C, and then (400 μL) of binding buffer was added. The flow cytometer (ACEA Biosciences Inc., San Jose, CA, USA) was utilized to perform a fluorometric examination of the immune-stained cancer cell lines.

#### 3.10.4. Cell Cycle Analysis

As previously explained, the cell cycle distribution shift caused by ATO suspension and ASN treatment was calculated using the flow cytometer [82].

T98G cells were kept in culture flasks of 25 cm^2^ at 1 × 10^6^ cells/mL density. After 24 h, the medium was replaced with either ATO suspension or ASN at their IC50. Control cells were given DMSO for 48 h. After incubation, trypsin-treated cells were harvested and rinsed two times with PBS. After fixation in cold ethanol (70%), cells were stained with 20 μg/mL PI, 0.1% Triton X-100, and 0.1 mg/mL RNase A in a 37 °C incubation. The BD Cell Quest Pro program was used to ascertain the percentage of cells in various phases of the cell cycle, analyzing 10,000 cells per sample.

### 3.11. Assessment of Inflammatory Biomarkers

By using specific ELISA kits (Cat. Nos. abx050220, Abbexa, Ca, UK), SEA563Ra, and SEA079Ra (Cloud-clone Corp., Houston, TX, USA), the levels of TNF-α, IL-1β, IL-6, TLR4, and NF-κB were measured to examine the efficacy of (ATO) suspension, (ATO-NLC F2), and ASN formulations. T98G cells were seeded at a density of 1 × 10⁵ cells per well in tissue culture plates and incubated for 48 h. Following incubation, the medium was discarded, and all the cells were treated first with 100 ng/mL of Lipopolysaccharide (LPS) used to provoke an inflammatory response, then distinct sets of cells treated with ATO suspension, ATO-NLC F2, and ASN at different doses (50, 150, and 300 µg/mL) with one group remaining untreated as positive control.

The test was conducted following the manufacturer’s protocol. The ELISA kits were combined with the samples according to the instructions. To quantify cytokine production and evaluate the immunogenic and apoptotic effects of the formulations, absorbance was measured with a microplate reader at wavelengths of 450 nm and 600 nm, respectively [83].

### 3.12. In Vivo Studies

Cairo University’s research ethics committee for the Faculty of Pharmacy approved the in vivo study plan (PI 2843). Weighing 2.5 ± 0.5 kg, New Zealand white rabbits were kept in an environment with relative humidity of 60 ± 10% and 23 ± 2 °C. The rabbits had full access to food and drink.

#### 3.12.1. In Vivo Pharmacokinetic Study

Nine rabbits were randomly assigned into 3 groups: G1 and G2 received ATO-NLC F2 intranasal hydrogel and oral ATO suspension, respectively, at a dose of 0.4 mg/kg, whereas G3 was administered 0.9% normal saline as control. The rabbit dose was calculated following this equation:

(3) $$\mathrm{Human}\;\mathrm{dose}(\mathrm{mg}/\mathrm{kg})=Animal\;dose\left(\frac{mg}{kg}\right)\times \frac{\left(Animal\;Km\right)}{\left(Human\;Km\right)}$$

The human conversion factor Km is 37, whereas the rabbit’s factor is 12 [84]. Microinjectors with soft polyethylene tubes with 0.10 mm inner diameters were used to administer the intranasal gel. After treatment, rabbits were supine with a 90-degree head angle for two minutes to prevent gel leakage. A 2 mL blood samples were withdrawn from the marginal ear vein into heparinized tubes at predetermined sampling intervals (0.5, 1, 2, 3, 4, 5, 6 h). The blood samples were instantaneously centrifuged at 3000 rpm for 15 min to separate plasma from blood samples [20].

#### 3.12.2. Sample Preparation

Rosuvastatin was used as an internal standard (IS) to construct the calibration curve of ATO in plasma. In summary, different amounts of ATO stock solution were mixed with 25 µL of Rosuvastatin stock solution (500 ng/mL). This mixture was then spiked into 225 µL of plasma to obtain the following concentration: (0.5, 1, 5, 10, 25, 50 ng/mL). To extract ATO from the samples, 250 µL of plasma samples were combined with 25 µL of (IS) and 2 mL of ethyl acetate to precipitate the proteins. This mixture was vortexed for 1 min and then centrifuged for 10 min at 3000 rpm. The supernatant was transferred to a fresh tube to get rid of the organic solvent by evaporating it using a vacuum concentrator (Eppendorf 5301, Hamburg, Germany). The dried residue was dissolved in 150 µL of the mobile phase, which consisted of a mixture of methanol and 0.1% formic acid in water (90:10, *v*/*v*). Subsequently, 10 µL of the resulting solution was injected into the LC-MS/MS system for analysis.

#### 3.12.3. LC/MS/MS Chromatographic Conditions

ATO was analyzed in the plasma sample utilizing UPLC MS/MS (Waters^®^ 3100, Milford, MA, USA) linked to TQ Detector (Acquity ultra-performance LC). Aliquot (10 μL) of the samples was injected into a Waters X BRIDGE^®^ BEH SHIELD RP C18 column (18 2.5 µm, 2.1 × 150 mm); Phenomenex, Torrance, CA, USA). The mobile phase consisted of methanol and 0.1% formic acid in water (90%:10%, *v*/*v*) at a 0.3 mL/min flow rate. The m/z value of the precursor to production of ATO was 559.54 to 440.3, while it was from 482.41 *m*/*z* to 258.17 *m*/*z* in the case of rosuvastatin. The results were evaluated utilizing analyst software (Mass Lynx V4.1, Milford, MA, USA).

#### 3.12.4. Pharmacokinetic and Statistical Analysis of Data

A pharmacokinetic study of plasma data was conducted using PKanalix Software (Monolix Suite 2020R1 by Lixoft, Antony, France) to assess the relative bioavailability of ATO after oral administration of ATO suspension and intranasal administration of ATO-NLC F2 hydrogel. The software was used to calculate ATO pharmacokinetic parameters, adopting a non-compartmental pharmacokinetic model. Peak plasma concentration (C_max_, ng/mL), corresponding time (T_max_, h), and area under the plasma concentration–time curve from time zero to the final time (AUC _0–12_, ng. h/mL) were estimated [41]. The calculated pharmacokinetic parameters were statistically analyzed via one-way analysis of variance (ANOVA) using IBM SPSS Statistics version 26, Armonk, NY, USA. The significance level was set at α = 0.05, with *p*-values ≤ 0.05 regarded as statistically significant.

#### 3.12.5. Brain Imaging Using Confocal Laser Scanning Microscopy (CLSM)

Rhodamine- B (RhB) dye was used to assess the targeting behavior of the formulated ASN. Briefly, the optimum NLC (F2) and SPION/NLC (ASN) were re-formulated using RhB instead of ATO and incorporated in HPMC hydrogel, as previously explained. The nine New Zealand white rabbits were randomly divided into three groups as follows.

Group I received the RhB solution as a control, group II received the formulated RhB-loaded NLC F2 intranasal hydrogel, while group III rabbits received the formulated RhB-loaded ASN intranasal hydrogel. Group III rabbits were allowed to lie in a supine position on a platform, with their heads directly placed on a neodymium–iron–boron magnet. One-hour post-treatment, the rabbits were sacrificed, and their brains were separated, washed with ringer’s solution, and sliced into 5 μm thick tissue using a microtome. CLSM (LSM 710, ZEN 2.3, Carl Zeiss, Oberkochen, Germany) was used to visually inspect tissue [84].

#### 3.12.6. Histopathological Study

The nine New Zealand white rabbits were randomly assigned to three groups as follows. Group I received 0.9% normal saline as a negative control, while groups II and III received ATO-NLC F2 and ASN hydrogels intranasally. The nasal tissues were then separated and histologically examined to evaluate the safety and biological compatibility of both hydrogels on the nasal epithelial cell membrane. Tissues were preserved in 10% neutral buffered formalin, and specimens underwent decalcification with 10% formic acid. Paraffin blocks were prepared through a series of steps: cutting, washing, dehydration using ethyl alcohol, clearing with xylene, and fixation at 70 °C. Microtome-cut slices (4–6 µm) were stained with hematoxylin and eosin (H&E). An Olympus BX43 microscope (Olympus Corporation, Tokyo, Japan) was used to look at the slides and find signs of edema, necrosis, or changes in the structure of the epithelial cell [20].

### 3.13. In Silico Studies

#### Atorvastatin (ATO)-Loaded Nanostructured Lipid Carrier (NLC) Formation and ADME Study

The PubChem database [85] was used to search for the 2D structure file (SDF) and SMILES of ATO, Caproyl 90, Lecithin, Pluronic f-68, and Glyceryl 1,2-dibehenate (Compritol). ATO and Compritol (lipid structure carrier of ATO) were further evaluated for their ADME properties using the predictive web tool ADMET Lab 2.0 [86] (Table S1). Final molecular dynamic simulation was performed using Molecular Operating Environment (MOE), 2019.0102 software, 2023 for ATO, Caproyl 90, Lecithin, Pluronic f-68, and Glyceryl 1,2-dibehenate (Compritol) [87]. The assumed 3D structure and binding affinity of ATO with NLC ingredients are predicted and studied using molecular dynamic simulation.

### 3.14. Network Pharmacology

#### 3.14.1. Screening of Atorvastatin (ATO) and Nanoparticle Formulation Ingredients and Gathering ATO Targets

ATO biological targets were predicted through the Swiss target prediction web tool (Table S2). Furthermore, the assembled biological targets (100 targets) were annotated using the UniProt database to gain the UniProt IDs of their genes [88].

#### 3.14.2. Gathering of Malignant Glioma Target Genes and Matching with Those of ATO for Cancer Treatment

The DisGeNET database [89] was searched for cancer-associated genes using the key term malignant glioma (Table S3). The Venn diagram intersection feature of FunRich 3.1.3 software was used to correlate cancer-associated genes with previously identified target genes of ATO [90].

#### 3.14.3. Constructing a Protein–Protein Interaction (PPI) Network

The STRING database facilitated the acquisition of a PPI network, while Cytoscape 3.9.0 software was employed for subsequent analysis and embellishment. Initially, prospective treatment targets for malignant glioma were included in the internet database STRING. The parameters and constraints were established to restrict the species to Homo sapiens and to apply a filter based on a combined score with a threshold of (≥ 0.95). The PPI network was acquired in TSV format, and the results were imported into the Cytoscape 3.9.0 software platform for network diagram visualization. Each network node was calculated, and core target genes were screened using the CytoHubba plug-in.

#### 3.14.4. KEGG Pathway Enrichment Assessment

The findings of the KEGG pathway assessment were evaluated using ShinyGO 0.8 [91]. A *p*-value level of 0.05 was employed for pathway discovery. The visualization analysis was performed using the online tools of the bioinformatics platform to make bubble charts.

#### 3.14.5. Molecular Docking Simulation Study (MDS)

The MOE 2019.0102, 2023 molecular docking model investigated ATO binding affinity against PI3K and MAPK3. Reduce energy, add hydrogen, and calculate partial charges to draw ATO. PDB IDs 4QTB and 5XJI contain PI3K and MAPK3. Molecular Operating software (MOE 2019.0102) prepared orders automatically and checked them using Amber10 Forcefield simulated docking. Visualization of poses and scoring function allowed ligand–protein complex interaction evaluation [92]. The validation of docking studies was examined using the root mean square deviation (RMSD) values for co-crystalized ligand protein isozymes (PI3K: 0.6217, MAPK3: 0.3826).

## 4. Conclusions

Glioblastoma therapy with an ATO/SPION-loaded NLC hydrogel administered intravenously has been shown to be effective. The optimal Formula ATO-LC F2 with 0.5% *w*/*w* Capryol 90, 1% *w*/*w* Compritol, and 1% *w*/*w* Pluronic F68 has a high EE%, Q6h%, and a low PS for direct brain passage. Thus, SPION was prepared to be combined with the optimal NLC (ASN) to improve brain-targeting. The prepared system (ASN) was added to an intranasal hydrogel for nasal mucosal retention. Cytotoxicity study revealed an enhanced cytotoxic effect with a 4.5 reduction in the IC50 compared to ATO suspension. Studying the cell cycle progression of the Glioblastoma cancer cell line (T98G) treated with the optimal ASN showed that they function mainly through the apoptotic mechanism and arrest of the G0-G1 phase. The pharmacokinetic study demonstrated that the prepared intranasal hydrogel ATO-NLC F2 could enhance the bioavailability of ATO by 2.28-fold compared to oral administration. Furthermore, ASN intranasal hydrogel showed a great significant reduction in TNF-α, IL-6, IL-1β, NF-kB, and TLR4 levels in the LPS-induced inflammatory model. The in silico studies were used to confirm the efficient ability of NLC to encapsulate the drug and improve its ADME properties. Furthermore, the comprehensive network pharmacology analysis revealed the top 10 glioblastoma-associated genes corresponding with the target genes of ATO. A docking simulation study using MOE software was conducted to elucidate the effect of ATO against two important pathways, including PI3K and MAPK, in the treatment of GBM. Finally, the findings of this study suggest that intranasal delivery of ATO/SPION-loaded NLC could be an effective technique for direct nose-to-brain transport of atorvastatin, potentially increasing its efficacy against GBM.

## Figures and Tables

**Table 1:** The experimental runs, the composition of ATO-loaded NLCs as per the constructed 3^2^ full factorial design and their dependent responses.

Formula Code	X_1_ Type of Liquid Lipid	X_2_ Type of SAA	Y_1_ EE% (% *w*/*w*)	Y_2_ PS (nm)	PDI	Y_3_ ZP (mV)	Y_4_ Q6h (%)
ATO-NLC F1	Capryol 90	Tween 80	92.85 ± 0.52	300.40 ± 0.65	0.448 ± 0.01	−19.10 ± 0.07	83.95 ± 1.83
ATO-NLC F2	Capryol 90	Pluronic F68	84.00 ± 0.67	282.50 ± 0.51	0.53 ± 0.08	−18.40 ± 0.15	89.23 ± 1.09
ATO-NLC F3	Capryol 90	Tween 80- Pluronic F68	72.18 ± 0.98	298.40 ± 3.28	0.57 ± 0.01	−22.20 ± 0.25	62.27 ± 2.07
ATO-NLC F4	Labrafil	Tween 80	89.40 ± 0.25	320.30 ± 3.64	0.54 ± 0.1	−18.80 ± 0.11	58.95 ± 1.87
ATO-NLC F5	Labrafil	Pluronic F68	83.02 ± 0.31	270.30 ± 0.54	0.48 ± 0.02	−22.05 ± 0.12	72.90 ± 1.72
ATO-NLC F6	Labrafil	Tween 80-Pluronic F68	68.73 ± 0.54	314.35 ± 2.42	0.51 ± 0.01	−18.20 ± 0.29	60.11 ± 1.06
ATO-NLC F7	Labrasol	Tween 80	78.75 ± 1.09	354.10 ± 2.72	0.51 ± 0.02	−21.20 ± 0.47	54.00 ± 1.95
ATO-NLC F8	Labrasol	Pluronic F68	72.37 ± 0.084	286.50 ± 2.43	0.48 ± 0.01	−18.20 ± 0.34	63.36 ± 2.67
ATO-NLC F9	Labrasol	Tween 80-Pluronic F68	58.05 ± 0.32	310.40 ± 3.63	0.56 ± 0.03	−17.20 ± 0.39	35.40 ± 1.77
ASN			85.40 ± 1.00	294.4 ± 0.81	0.48 ± 0.08	−14.80 ± 0.15	78.00 ± 0.16
SPION			-	10.32 ± 1.04	-	21.30 ± 0.68	-

**Table 2:** Regression assessment of the measured responses in accordance with the best-fitting model.

Response	EE%	PS (nm)	ZP (mv)	Q6h %
Model	Main Effects	2FI	2FI	2FI
F-value	21.82	56.07	30.62	20.40
*p*-value	˂0.0001	˂0.0001	˂0.0001	˂0.0001
Adequate Precision	13.736	26.018	13.732	15.014
R^2^	0.870	0.980	0.965	0.948
Adjusted (R^2^)	0.831	0.962	0.933	0.901
Predicted (R^2^)	0.751	0.921	0.858	0.791
Significant Factors	X_1_, X_2_	X_1_, X_2_, X_1_X_2_	X_1_	X_1_, X_2_, X_1_X_2_
Actual values (ATO-NLC F2)	84.0%	282.50	−18.40	89.23

**Table 3:** Stability Study data of ATO-NLC F2 and ASN before and after 90 days of storage at 25 °C and 4 °C.

Parameters	ATO-NLC F2 Freshly Prepared	ATO-NLC F2 After a Storage Period of 3 Months at 25 °C	ATO-NLC F2 After a Storage Period of 3 Months at 4 °C
EE%	84.0 ± 0.67	77.41 ± 0.40	82.76 ± 0.92
PS (nm)	282.5.60 ± 0.51	288.5 ± 0.57	284.40 ± 0.54
ZP (mV)	−18.40 ± 0.15	−17.8 ± 0.01	−17.7 ± 2.67
Q6h (%)	89.230 ± 1.09	84.04 ± 0.25	88.97 ± 1.70
PDI	0.531 ± 0.08	0.527 ± 0.71	0.568 ± 0.0
**Parameters**	**ASN** **Freshly prepared**	**ASN** **After a storage period of 3 months at 25 °C**	**ASN** **After a storage period of 3 months at 4 °C**
EE%	85.40 ± 1.00	83.36 ± 0.23	85.01 ± 0.65
PS (nm)	294.4 ± 0.81	313.4 ± 0.71	293.6 ± 0.21
ZP (mV)	−14.80 ± 0.15	−14.1 ± 0.12	−14.6 ± 0.76
Q6h (%)	78.00 ± 0.16	75.74 ± 0.15	78.60 ± 0.12
PDI	0.488 ± 0.08	0.514 ± 0.13	0.316 ± 0.05

Abbreviations: EE%: entrapment efficiency %,PS: Particle size, ZP: zeta potential, Q6h: the released percent of drug after 6 h, PDI: polydispersity index, ATO: atorvastatin, NLCs: nanostructure lipid carriers, ASN: atorvastatin/SPION-loaded NLC.

**Table 4:** The correlation coefficient (R^2^) values after fitting the in vitro release data of atorvastatin from ATO-NLC F2, ASN, and prepared intranasal hydrogel to different kinetic release models.

Model	ATO-NLC F2	ASN	ASN Hydrogel	ATO Suspension
Zero Order	0.9620	0.9544	0.9525	0.9785
First Order	0.9191	0.9225	0.9141	0.9210
Second Order	0.8066	0.9688	0.9443	0.8029
Baker–Lonsdale	0.9438	0.8538	0.8636	0.9549
Higuchi Model	0.9935	0.9725	0.8924	0.9757

Abbreviations: ATO: atorvastatin, NLCs: nanostructure lipid carriers, ASN: atorvastatin/SPION-loaded NLC.

**Table 5:** Pharmacokinetic parameters of atorvastatin after administration of optimized intranasal hydrogel (ATO-NLC F2) and oral suspension.

Parameters.	ATO-NLC F2 Nasal Gel	ATO-Oral Suspension
Cp_max_ (ng. mL^−1^)	7.89 ± 0.97	2.37 ± 0.54
AUC_0-t_ (h. ng.mL^−1^)	17.92 ± 0.74	7.86 ± 1.41
AUC_0→∞_ (h. ng.mL^−1^)	19.32 ± 0.48	8.95 ± 1.16
T_max_ (h)	0.50 ± 00	1 ± 00
% Relative BA	228.128%	

Abbreviations: ATO: atorvastatin, Cp_max_: maximum plasma concentration, T_max_: time to reach C_max_, AUC: area under the curve, BA: bioavailability. Notes: data are presented as mean ± SD (*n* = 3).

**Table 6:** ADME properties of ATO and Compritol as a lipid component of NLC as per the predictive web tool ADMET Lab 2.0.

	Absorption	Distribution	Metabolism	Excretion
	Pgp	BBB Penetration	CYP 1A2/2C19/2C9/2D6/3A4 Substrate	Clearance (CL)
ATO	Pgp substrate	pass BBB	CYP2C9 substrate CYP3A4 substrate	6.746 (moderate CL)
Compritol	Non-Pgp substrate	pass BBB	CYP2C9 substrate	4.554 (low CL)

**Table 7:** Results of docking simulations of ATO with target enzymes (MAPK3 and PI3K).

Target Enzymes	Binding Score kcal/mol	Key Amino Acid Residues	Binding Type	Representation in Two-Dimensional
MAPK3	−7.8959	Cys138	Hydrogen Bonding	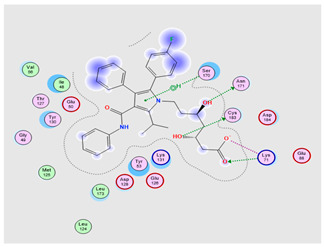
		Lys71	Hydrogen Bonding	
		Lys71	Ionic	
		Asn171	Hydrogen Bonding	
		Ser170	Hydrophobic	
PI3K	−7.0106	Ser774	Hydrogen Bonding,	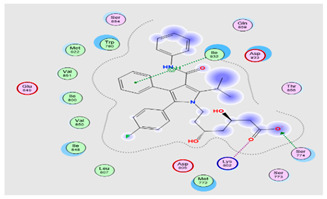
		Lys802	Ionic	
		Ile932	Hydrophobic	

**Table 8:** The independent variables affecting ATO-loaded NLCs as per a 3^2^ full factorial design and the optimization criteria of the responses.

Factors (Independent Variables)	Levels		
X_1_: Type of liquid lipid	Capryol 90	Labrafil	Labrasol
X_2_: Type of SAA	Tween 80	Pluronic F68	Tween 80-Pluronic F68
**Responses** **(dependent Variables)**	**Constraints**		
Y_1_: EE (%)	Maximize		
Y_2_: PS (nm)	Minimize		
Y_3_: ZP (Mv)	Maximize (absolute value)		
Y_4_: Q6h (%)	Maximize		

Abbreviations: SAA: surfactants, PS: particle size, EE%: entrapment efficacy, ZP: zeta potential, Q6h: the released percent of drug after 6 h, ATO: atorvastatin, and NLCs: nanostructured lipid carriers.

## Data Availability

The authors confirm that the data supporting the findings of this study are available within the article.

## Supplementary Materials

The following supporting information can be downloaded at: https://www.mdpi.com/article/10.3390/ph18030421/s1, Figure S1: Apoptosis; Figure S2: EGFR Tyrosine Kinase inhibitor resistance pathway; Figure S3: Glioma; Figure S4: pathways in cancer; Figure S5: PI3K-Akt signal pathway; Figure S6: Toll-like receptor signaling pathway; Table S1: ADMET Lab ADME PREDICTION; Table S2: ATO predicted targets; Table S3: Malignant Glioma Involved Genes; Table S4: Venn intersecting Common Targets: Table S5: Top 10 GENES in network ranked by Degree method; Table S6: Enriched Pathways.

## Abbreviations

The following abbreviations are used in this manuscript:

GBM	Glioblastoma Multiforme
IN	Intranasal
BBB	Blood–Brain Barrier
ATO	Atorvastatin
NLC	Nanostructured Lipid Carrier
EE%	Entrapment Efficiency Percentage
PS	Particle Size
ZP	Zeta Potential
Q6h	Percentage of Drug Release After 6 Hours
2FI	two-factor interaction
BCS	Biopharmaceutics Classification System
MCT	Medium-Chain Triglycerides
DoE	Design of Experiments
ASN	ATO/SPION loaded NLC
PBS	Phosphate-Buffer Saline
HPMC	Hydroxypropyl Methylcellulose
TEM	Transmission Electron Microscopy
PDI	Polydispersity Index
SPSS	Statistical Package for Social Sciences
ATCC	American Type Culture Collection
FBS	Fetal Bovine Serum
MTT	3-(4,5-dimethylthiazol-2-yl)-2,5-diphenyltetrazolium bromide
IC50	Inhibitory Concentration 50
FITC	Fluorescein Isothiocyanate
PI	Propidium Iodide
ELISA	Enzyme-Linked Immunosorbent Assay
TNF-α-alpha	Tumor Necrosis Factor-alpha
IL-1β	Interleukin-1 beta
IL-6	Interleukin-6
SDS-PAGE	Sodium Dodecyl Sulfate Polyacrylamide Gel Electrophoresis
SPION	superparamagnetic iron oxide
DMSO	dimethyl sulfoxide
