# Supplementary material for: Enhanced Intranasal Delivery of Atorvastatin via Superparamagnetic Iron-Oxide-Loaded Nanocarriers: Cytotoxicity and Inflammation Evaluation and In Vivo, In Silico, and Network Pharmacology Study for Targeting Glioblastoma Management
Source: Pharmaceuticals (Basel). 2025 Mar 16;18(3):421. doi: 10.3390/ph18030421 (PMC11944838; doi:10.3390/ph18030421)
Supplement: Supplementary file 1 [file pharmaceuticals-18-00421-s001.zip › pharmaceuticals-3505180-supplementary.pdf]

Enhanced Intranasal Delivery of Atorvastatin via Superparamagnetic Iron-Oxide Loaded Nanocarriers: Cytotoxicity, Inflammation Evaluation, *In-Vivo* Study, In Silico and Network Pharmacology Study for Targeting Glioblastoma Management

(Supplementary tables)

	smiles	LogP	Pgp-inh	Pgp-sub	BBB	CYP1A2-inh	CYP1A2-sub	CYP2C19-inh	CYP2C19-sub	CYP2C9-inh	CYP2C9-sub	CYP2D6-inh	CYP2D6-sub	CYP3A4-inh	CYP3A4-sub	CL
**Compitrol**	[H]C([H])(C(OC([H])([C@@](C([H])(O[H])[H])(OC(C([H])([H])[H])[H])[H])[H])[H])[H])[H])[H])[H])[H])[H])[H])[H])[H])[H])[H])[H])[H])[H])[H])[H])=O)[H])[H])=O)[H])[H])[H])[H])[H])[H])[H])[H])[H])[H])[H])[H])[H])[H])[H])[H])[H])[H])[H])[H])[H]	16.421	0	0.003	0	0.009	0.088	0.06	0.029	0.004	0.986	0.018	0.002	0.107	0.006	4.554
**ATO**	CC(C)C1=C(C(=C(N1CCC(CC(CC(=O)O)O)O)C2=CC=C(C=C2)F)C3=CC=CC=C3)C(=O)NC4=CC=CC=C4	4.51	0.043	0.943	0.056	0.311	0.099	0.428	0.095	0.857	0.961	0.074	0.299	0.075	0.729	6.746

1. **Supplementary Table S1: ADMET Lab ADME PREDICTION**

2. Supplementary Table S2: ATO predicted targets

Target	Common name	Uniprot ID	ChEMBL ID	Target Class	Probability*	Known actives (3D/2D)
Cytochrome P450 3A4	CYP3A4	P08684	CHEMBL340	Cytochrome P450	1	4 / 2Â Â Â Â Â
HMG-CoA reductase	HMGCR	P04035	CHEMBL402	Oxidoreductase	1	459 / 220Â Â Â Â Â
Histone deacetylase 6	HDAC6	Q9UBN7	CHEMBL1865	Eraser	0.404267953	24 / 5Â Â Â Â Â
Histone deacetylase 2	HDAC2	Q92769	CHEMBL1937	Eraser	0.404267953	11 / 7Â Â Â Â Â
Histone deacetylase 1	HDAC1	Q13547	CHEMBL325	Eraser	0.404267953	41 / 9Â Â Â Â Â
Phosphodiesterase 6D	PDE6D	O43924	CHEMBL3860	Phosphodiesterase	0.163521902	1 / 7Â Â Â Â Â
Squalene synthetase	FDFT1	P37268	CHEMBL3338	Enzyme	0.074392272	105 / 0Â Â Â Â Â
Glucocorticoid receptor	NR3C1	P04150	CHEMBL2034	Nuclear receptor	0.074392272	53 / 0Â Â Â Â Â
Prostanoid EP4 receptor (by homology)	PTGER4	P35408	CHEMBL1836	Family A G protein-coupled receptor	0.074392272	301 / 0Â Â Â Â Â
Phosphodiesterase 5A	PDE5A	O76074	CHEMBL1827	Phosphodiesterase	0.074392272	46 / 0Â Â Â Â Â
Prostanoid IP receptor	PTGIR	P43119	CHEMBL1995	Family A G protein-coupled receptor	0.074392272	159 / 0Â Â Â Â Â
Estrogen receptor beta	ESR2	Q92731	CHEMBL242	Nuclear receptor	0.074392272	35 / 0Â Â Â Â Â
Prostanoid FP receptor	PTGFR	P43088	CHEMBL1987	Family A G protein-coupled receptor	0.074392272	40 / 0Â Â Â Â Â
Prostanoid EP3 receptor	PTGER3	P43115	CHEMBL3710	Family A G protein-coupled receptor	0.074392272	51 / 0Â Â Â Â Â

11-beta-hydroxysteroid dehydrogenase 1	HSD11B1	P28845	CHEMBL4235	Enzyme	0.074392272	106 / 0Â Â Â Â Â
Cytosolic phospholipase A2	PLA2G4A	P47712	CHEMBL3816	Enzyme	0.074392272	136 / 0Â Â Â Â Â
Prostanoid EP1 receptor	PTGER1	P34995	CHEMBL1811	Family A G protein-coupled receptor	0.074392272	98 / 0Â Â Â Â Â
c-Jun N-terminal kinase 3	MAPK10	P53779	CHEMBL2637	Kinase	0.074392272	27 / 0Â Â Â Â Â
TNF-alpha	TNF	P01375	CHEMBL1825	Secreted protein	0.074392272	13 / 0Â Â Â Â Â
Egl nine homolog 1	EGLN1	Q9GZT9	CHEMBL5697	Oxidoreductase	0.074392272	24 / 0Â Â Â Â Â
Vitamin D receptor	VDR	P11473	CHEMBL1977	Nuclear receptor	0.074392272	11 / 0Â Â Â Â Â
Matrix metalloproteinase 1	MMP1	P03956	CHEMBL332	Protease	0.074392272	31 / 0Â Â Â Â Â
Peroxisome proliferator-activated receptor alpha	PPARA	Q07869	CHEMBL239	Nuclear receptor	0.074392272	338 / 0Â Â Â Â Â
Growth factor receptor-bound protein 2	GRB2	P62993	CHEMBL3663	Other cytosolic protein	0.074392272	65 / 0Â Â Â Â Â
Peroxisome proliferator-activated receptor delta	PPARD	Q03181	CHEMBL3979	Nuclear receptor	0.074392272	255 / 0Â Â Â Â Â
Gastric inhibitory polypeptide receptor	GIPR	P48546	CHEMBL4383	Family B G protein-coupled receptor	0.074392272	16 / 0Â Â Â Â Â
Leukotriene B4 receptor 1	LTB4R	Q15722	CHEMBL3911	Family A G protein-coupled receptor	0.074392272	161 / 0Â Â Â Â Â
Aldo-keto-reductase family 1 member C3	AKR1C3	P42330	CHEMBL4681	Enzyme	0.074392272	32 / 0Â Â Â Â Â
Bile acid receptor FXR	NR1H4	Q96RI1	CHEMBL2047	Nuclear receptor	0.074392272	190 / 0Â Â Â Â Â
Phosphodiesterase 4A	PDE4A	P27815	CHEMBL254	Phosphodiesterase	0.074392272	22 / 0Â Â Â Â Â
Phosphodiesterase 4B	PDE4B	Q07343	CHEMBL275	Phosphodiesterase	0.074392272	125 / 0Â Â Â Â Â

Phosphodiesterase 4D	PDE4D	Q08499	CHEMBL288	Phosphodiesterase	0.074392272	96 / 0Â Â Â Â Â
Nitric oxide synthase, inducible (by homology)	NOS2	P35228	CHEMBL4481	Enzyme	0.074392272	8 / 0Â Â Â Â Â
Caspase-1	CASP1	P29466	CHEMBL4801	Protease	0.074392272	254 / 0Â Â Â Â Â
Retinoic acid receptor gamma	RARG	P13631	CHEMBL2003	Nuclear receptor	0.074392272	29 / 0Â Â Â Â Â
Retinoic acid receptor beta	RARB	P10826	CHEMBL2008	Nuclear receptor	0.074392272	38 / 0Â Â Â Â Â
Protein-tyrosine phosphatase 1B	PTPN1	P18031	CHEMBL335	Phosphatase	0.074392272	333 / 0Â Â Â Â Â
Tyrosine-protein kinase receptor TYRO3	TYRO3	Q06418	CHEMBL5314	Kinase	0.074392272	3 / 0Â Â Â Â Â
Leukocyte adhesion glycoprotein LFA-1 alpha	ITGAL	P20701	CHEMBL1803	Adhesion	0.074392272	32 / 0Â Â Â Â Â
Caspase-3	CASP3	P42574	CHEMBL2334	Protease	0.074392272	187 / 0Â Â Â Â Â
Integrin alpha-4	ITGA4	P13612	CHEMBL278	Membrane receptor	0.074392272	103 / 0Â Â Â Â Â
Caspase-7	CASP7	P55210	CHEMBL3468	Protease	0.074392272	68 / 0Â Â Â Â Â
Caspase-8	CASP8	Q14790	CHEMBL3776	Protease	0.074392272	61 / 0Â Â Â Â Â
Carnitine O-palmitoyltransferase 1, liver isoform	CPT1A	P50416	CHEMBL1293194	Enzyme	0.074392272	202 / 0Â Â Â Â Â
Ileal bile acid transporter	SLC10A2	Q12908	CHEMBL2778	Electrochemical transporter	0.074392272	56 / 0Â Â Â Â Â
Prostaglandin I2 synthase	PTGIS	Q16647	CHEMBL4428	Cytochrome P450	0.074392272	14 / 0Â Â Â Â Â
Liver glycogen phosphorylase	PYGL	P06737	CHEMBL2568	Enzyme	0.074392272	14 / 0Â Â Â Â Â
Endothelin receptor ET-B	EDNRB	P24530	CHEMBL1785	Family A G protein-coupled receptor	0.074392272	92 / 0Â Â Â Â Â

FK506-binding protein 1A	FKBP1A	P62942	CHEMBL1902	Isomerase	0.074392272	66 / 0Â Â Â Â Â
Matrix metalloproteinase 13	MMP13	P45452	CHEMBL280	Protease	0.074392272	200 / 0Â Â Â Â Â
Matrix metalloproteinase 9	MMP9	P14780	CHEMBL321	Protease	0.074392272	50 / 0Â Â Â Â Â
Matrix metalloproteinase 12	MMP12	P39900	CHEMBL4393	Protease	0.074392272	85 / 0Â Â Â Â Â
Carnitine O-palmitoyltransferase 1, muscle isoform	CPT1B	Q92523	CHEMBL2216739	Group translocator	0.074392272	145 / 0Â Â Â Â Â
Hematopoietic cell protein-tyrosine phosphatase 70Z-PEP	PTPN22	Q9Y2R2	CHEMBL2889	Phosphatase	0.074392272	58 / 0Â Â Â Â Â
Peptidyl-prolyl cis-trans isomerase FKBP5	FKBP5	Q13451	CHEMBL2052031	Enzyme	0.074392272	16 / 0Â Â Â Â Â
Complement factor D	CFD	P00746	CHEMBL2176771	Protease	0.074392272	28 / 0Â Â Â Â Â
Lysophosphatidic acid receptor Edg-7	LPAR3	Q9UBY5	CHEMBL3250	Family A G protein-coupled receptor	0.074392272	5 / 0Â Â Â Â Â
Lysophosphatidic acid receptor Edg-2	LPAR1	Q92633	CHEMBL3819	Family A G protein-coupled receptor	0.074392272	6 / 0Â Â Â Â Â
FK506 binding protein 4	FKBP4	Q02790	CHEMBL4050	Enzyme	0.074392272	10 / 0Â Â Â Â Â
Steroid 5-alpha-reductase 2	SRD5A2	P31213	CHEMBL1856	Oxidoreductase	0.074392272	31 / 0Â Â Â Â Â
GABA transporter 1	SLC6A1	P30531	CHEMBL1903	Electrochemical transporter	0.074392272	223 / 0Â Â Â Â Â
Plasminogen activator inhibitor-1	SERPINE1	P05121	CHEMBL3475	Secreted protein	0.074392272	38 / 0Â Â Â Â Â
Transitional endoplasmic reticulum ATPase	VCP	P55072	CHEMBL1075145	Primary active transporter	0.074392272	35 / 0Â Â Â Â Â
Histamine H1 receptor	HRH1	P35367	CHEMBL231	Family A G protein-coupled receptor	0.074392272	67 / 0Â Â Â Â Â
Phospholipase A2 group IIA	PLA2G2A	P14555	CHEMBL3474	Enzyme	0.074392272	42 / 0Â Â Â Â Â

LXR-alpha	NR1H3	Q13133	CHEMBL2808	Nuclear receptor	0.074392272	8 / 0Â Â Â Â Â
Caspase-6	CASP6	P55212	CHEMBL3308	Protease	0.074392272	7 / 0Â Â Â Â Â
LXR-beta	NR1H2	P55055	CHEMBL4093	Nuclear receptor	0.074392272	7 / 0Â Â Â Â Â
Solute carrier family 22 member 6 (by homology)	SLC22A6	Q4U2R8	CHEMBL1641347	Electrochemical transporter	0.074392272	1 / 0Â Â Â Â Â
Thyroid hormone receptor alpha	THRA	P10827	CHEMBL1860	Nuclear receptor	0.074392272	97 / 0Â Â Â Â Â
Integrin alpha-V/beta-3	ITGAV ITGB3	P06756 P05106	CHEMBL1907598	Membrane receptor	0.074392272	582 / 0Â Â Â Â Â
Thyroid hormone receptor beta-1	THRB	P10828	CHEMBL1947	Nuclear receptor	0.074392272	106 / 0Â Â Â Â Â
Retinoid X receptor alpha (by homology)	RXRA	P19793	CHEMBL2061	Nuclear receptor	0.074392272	35 / 0Â Â Â Â Â
Folate receptor alpha	FOLR1	P15328	CHEMBL2121	Membrane receptor	0.074392272	7 / 0Â Â Â Â Â
C-C chemokine receptor type 1	CCR1	P32246	CHEMBL2413	Family A G protein-coupled receptor	0.074392272	9 / 0Â Â Â Â Â
Carnitine palmitoyltransferase 2	CPT2	P23786	CHEMBL3238	Enzyme	0.074392272	62 / 0Â Â Â Â Â
Epoxide hydratase	EPHX2	P34913	CHEMBL2409	Protease	0.074392272	78 / 0Â Â Â Â Â
Leukocyte elastase	ELANE	P08246	CHEMBL248	Protease	0.074392272	58 / 0Â Â Â Â Â
PI3-kinase p110-beta subunit	PIK3CB	P42338	CHEMBL3145	Enzyme	0.074392272	4 / 0Â Â Â Â Â
Matrix metalloproteinase 2	MMP2	P08253	CHEMBL333	Protease	0.074392272	149 / 0Â Â Â Â Â
MAP kinase ERK1 (by homology)	MAPK3	P27361	CHEMBL3385	Kinase	0.074392272	2 / 0Â Â Â Â Â
Glucagon	GCG	P01275	CHEMBL5736	Unclassified protein	0.074392272	55 / 0Â Â Â Â Â

Signal transducer and activator of transcription 5B	STAT5B	P51692	CHEMBL5817	Transcription factor	0.074392272	9 / 0Â Â Â Â Â
Cholecystokinin B receptor	CCKBR	P32239	CHEMBL298	Family A G protein-coupled receptor	0.074392272	348 / 9Â Â Â Â Â
Integrin alpha-IIb/beta-3	ITGA2B ITGB3	P08514 P05106	CHEMBL2093869	Membrane receptor	0.074392272	398 / 0Â Â Â Â Â
Protein-tyrosine phosphatase LC-PTP	PTPN7	P35236	CHEMBL2219	Phosphatase	0.074392272	12 / 0Â Â Â Â Â
Phosphodiesterase 3	PDE3A	Q14432	CHEMBL241	Phosphodiesterase	0.074392272	1 / 0Â Â Â Â Â
Beta secretase 2	BACE2	Q9Y5Z0	CHEMBL2525	Protease	0.074392272	2 / 0Â Â Â Â Â
Phosphodiesterase 3B	PDE3B	Q13370	CHEMBL290	Phosphodiesterase	0.074392272	1 / 0Â Â Â Â Â
Protein-tyrosine phosphatase 1C	PTPN6	P29350	CHEMBL3166	Phosphatase	0.074392272	13 / 0Â Â Â Â Â
Leukocyte common antigen	PTPRC	P08575	CHEMBL3243	Enzyme	0.074392272	17 / 0Â Â Â Â Â
Hexokinase type IV	GCK	P35557	CHEMBL3820	Enzyme	0.074392272	52 / 0Â Â Â Â Â
Beta-secretase 1	BACE1	P56817	CHEMBL4822	Protease	0.074392272	50 / 0Â Â Â Â Â
Lysophosphatidic acid receptor Edg-4	LPAR2	Q9HBW0	CHEMBL3724	Family A G protein-coupled receptor	0.074392272	9 / 0Â Â Â Â Â
Discoidin domain-containing receptor 2	DDR2	Q16832	CHEMBL5122	Kinase	0.074392272	6 / 0Â Â Â Â Â
Calcium sensing receptor	CASR	P41180	CHEMBL1878	Family C G protein-coupled receptor	0.074392272	39 / 0Â Â Â Â Â
Purinergic receptor P2Y12	P2RY12	Q9H244	CHEMBL2001	Family A G protein-coupled receptor	0.074392272	170 / 0Â Â Â Â Â
Leukotriene A4 hydrolase	LTA4H	P09960	CHEMBL4618	Protease	0.074392272	87 / 0Â Â Â Â Â
Receptor-type tyrosine-protein phosphatase S	PTPRS	Q13332	CHEMBL2396508	Phosphatase	0.074392272	8 / 0Â Â Â Â Â

Dual specificity protein phosphatase 3	DUSP3	P51452	CHEMBL2635	Phosphatase	0.074392272	34 / 0Â Â Â Â Â

3. Supplementary Table S3: Malignant Glioma Involved Genes

Disease	Disease_id	Gene	Gene_id	UniProt	Gene_ Full_Name	Protein_Class	N_diseases_g	DSI_g	DPI_g	pLI	Score_gda	EL_gda	EI_gda	N_PMIDs	N_SNPs_gda	First_Ref	Last_Ref
Malignant Glioma	C0555198	TP53	7157	P04637	tumor protein p53	Transcription factor	2494	0.236	0.962	0.5324	0.6		1	57	1	1992	2019
Malignant Glioma	C0555198	SPP1	6696	P10451	secreted phosphoprotein 1		824	0.353	0.885	2E-06	0.56		1	7	0	1998	2018
Malignant Glioma	C0555198	HRAS	3265	P01112	HRas proto-oncogene, GTPase	Enzyme modulator	698	0.378	0.885	0.0798	0.5		1	1	0	2018	2018
Malignant Glioma	C0555198	IDH1	3417	O75874	isocitrate dehydrogenase (NADP(+)) 1		399	0.424	0.808	3E-12	0.4		1	34	2	2009	2019
Malignant Glioma	C0555198	MGMT	4255	P16455	O-6-methylguanine-DNA methyltransferase		444	0.412	0.885	1E-07	0.4		0.971	34	2	1992	2019
Malignant Glioma	C0555198	TNFSF10	8743	P50591	TNF superfamily member 10	Signaling	445	0.413	0.885	0.135	0.4		1	13	0	2000	2014
Malignant Glioma	C0555198	PTEN	5728	P60484	phosphatase and tensin homolog	Enzyme	1349	0.305	0.923	0.2565	0.4		1	17	0	1998	2018
Malignant Glioma	C0555198	CDKN2A	1029	P42771;Q8N726	cyclin dependent kinase inhibitor 2A		1314	0.3	0.885	0.3947	0.4		0.957	23	0	1994	2019
Malignant Glioma	C0555198	TERT	7015	O14746	telomerase reverse transcriptase	Enzyme	703	0.374	0.846	0.9905	0.39		1	11	0	2001	2016
Malignant Glioma	C0555198	TNF	7124	P01375	tumor necrosis factor	Signaling	2724	0.231	0.962	0.8033	0.36		1	7	0	1993	2019
Malignant Glioma	C0555198	CDKN2B	1030	P42772	cyclin dependent kinase inhibitor 2B		440	0.417	0.769	0.0073	0.34		1	6	0	1995	2009
Malignant Glioma	C0555198	PTGS2	5743	P35354	prostaglandin-endoperoxide synthase 2	Enzyme	1234	0.314	0.962	0.996	0.34		1	5	0	2005	2017
Malignant Glioma	C0555198	FGF2	2247	P09038	fibroblast growth factor 2	Signaling	635	0.383	0.923	0.0184	0.34		1	5	0	1997	2011
Malignant Glioma	C0555198	BRAF	673	P15056	B-Raf proto-oncogene, serine/threonine kinase	Kinase	1228	0.319	0.846	1	0.34		1	5	2	2013	2019
Malignant Glioma	C0555198	RAF1	5894	P04049	Raf-1 proto-oncogene, serine/threonine kinase	Kinase	470	0.418	0.885	0.853	0.33		1	4	0	2006	2013
Malignant Glioma	C0555198	GDNF	2668	P39905	glial cell derived neurotrophic factor	Signaling	409	0.434	0.885	0.2035	0.33		1	3	0	2009	2017

Malignant Glioma	C0555198	ERBB2	2064	P04626	erb-b2 receptor tyrosine kinase 2	Kinase	995	0.328	0.923	0.006	0.33		1	3	0	2005	2012
Malignant Glioma	C0555198	SLC5A5	6528	Q92911	solute carrier family 5 member 5	Transporter	195	0.499	0.769	0.0029	0.32		1	3	0	2003	2015
Malignant Glioma	C0555198	NTRK1	4914	P04629	neurotrophic receptor tyrosine kinase 1	Kinase	443	0.422	0.808	2E-06	0.32		1	3	0	2014	2019
Malignant Glioma	C0555198	ROS1	6098	P08922	ROS proto-oncogene 1, receptor tyrosine kinase	Kinase	356	0.439	0.885	2E-72	0.32		1	2	0	2009	2017
Malignant Glioma	C0555198	PPM1D	8493	O15297	protein phosphatase, Mg2+/Mn2+ dependent 1D	Enzyme	177	0.513	0.808	2E-11	0.32		1	3	0	2012	2016
Malignant Glioma	C0555198	CHI3L1	1116	P36222	chitinase 3 like 1		420	0.419	0.885	1E-05	0.32		1	3	0	2011	2016
Malignant Glioma	C0555198	ATRX	546	P46100	ATRX chromatin remodeler		412	0.452	0.808	1	0.32		1	4	0	2013	2019
Malignant Glioma	C0555198	STMN1	3925	P16949	stathmin 1		284	0.458	0.885	0.0752	0.31		1	1	0	2007	2007
Malignant Glioma	C0555198	MDK	4192	P21741	midkine	Signaling	209	0.494	0.808	0.2301	0.31		1	2	0	2004	2011
Malignant Glioma	C0555198	FGFR1	2260	P11362	fibroblast growth factor receptor 1	Kinase	816	0.362	0.885	0.9998	0.31		1	2	0	2013	2019
Malignant Glioma	C0555198	ATP1B2	482	P14415	ATPase Na+/K+ transporting subunit beta 2	Transporter	28	0.705	0.308	0.0737	0.31		1	2	0	2003	2006
Malignant Glioma	C0555198	NTRK3	4916	Q16288	neurotrophic receptor tyrosine kinase 3	Kinase	150	0.522	0.692	0.9983	0.31		1	1	0	2014	2014
Malignant Glioma	C0555198	CAT	847	P04040	catalase	Enzyme	794	0.359	0.962	2E-10	0.31		1	2	0	2011	2013
Malignant Glioma	C0555198	H3-3A	3020	P84243	H3.3 histone A		134	0.559	0.692	0.1231	0.31		1	3	3	2012	2019
Malignant Glioma	C0555198	HLA-A	3105	P04439	major histocompatibility complex, class I, A		672	0.37	0.846	0.0013	0.31		1	2	0	1994	2003
Malignant Glioma	C0555198	ACVR1	90	Q04771	activin A receptor type 1	Kinase	144	0.535	0.731	0.3254	0.31		1	3	0	2014	2014
Malignant Glioma	C0555198	PLK1	5347	P53350	polo like kinase 1	Kinase	253	0.467	0.808	0.9785	0.31		1	1	0	2012	2012
Malignant Glioma	C0555198	RTEL1	51750	Q9NZ71	regulator of telomere elongation helicase 1	Enzyme	321	0.459	0.808	9E-10	0.31		1	3	0	2009	2014
Malignant Glioma	C0555198	SOD1	6647	P00441	superoxide dismutase 1	Enzyme	689	0.379	0.962	0.1773	0.31		1	2	0	1999	2002

Malignant Glioma	C0555198	CLCN2	1181	P51788	chloride voltage-gated channel 2	Ion channel	67	0.626	0.462	6E-12	0.3		1	1	0	2003	2003
Malignant Glioma	C0555198	CCR4	1233	P51679	C-C motif chemokine receptor 4	G-protein coupled receptor	168	0.51	0.731	0.0686	0.3		1	1	0	2008	2008
Malignant Glioma	C0555198	FBXW7	55294	Q969H0	F-box and WD repeat domain containing 7	Enzyme modulator	257	0.465	0.808	0.9995	0.3		1	1	0	2008	2008
Malignant Glioma	C0555198	NF1	4763	P21359	neurofibromin 1	Enzyme modulator	380	0.44	0.885	0.9018	0.3		1	1	0	2014	2014
Malignant Glioma	C0555198	FAM107A	11170	O95990	family with sequence similarity 107 member A		36	0.674	0.462	8E-07	0.3		1	1	0	2006	2006
Malignant Glioma	C0555198	CLCN5	1184	P51795	chloride voltage-gated channel 5	Ion channel	101	0.593	0.577	0.9924	0.3		1	1	0	2003	2003
Malignant Glioma	C0555198	TFRC	7037	P02786	transferrin receptor	Enzyme	359	0.437	0.885	7E-05	0.3		1	1	0	2006	2006
Malignant Glioma	C0555198	XAF1	54739	Q6GPH4	XIAP associated factor 1		83	0.587	0.769	7E-12	0.3		1	1	0	2017	2017
Malignant Glioma	C0555198	TRIM29	23650	Q14134	tripartite motif containing 29		70	0.606	0.654	8E-08	0.3		1	1	0	2019	2019
Malignant Glioma	C0555198	DMXL1	1657	Q9Y485	Dmx like 1		8	0.805	0.154	1	0.3		1	1	0	2006	2006
Malignant Glioma	C0555198	CCDC26	137196	Q8TAB7	CCDC26 long non-coding RNA		60	0.647	0.615		0.3		1	1	0	2009	2009
Malignant Glioma	C0555198	FTH1	2495	P02794	ferritin heavy chain 1	Storage	107	0.559	0.731	0.14	0.3		1	1	0	2011	2011
Malignant Glioma	C0555198	NTRK2	4915	Q16620	neurotrophic receptor tyrosine kinase 2	Kinase	284	0.479	0.808	1	0.3		1	1	0	2014	2014
Malignant Glioma	C0555198	BRCA2	675	P51587	BRCA2 DNA repair associated	Nucleic acid binding	656	0.379	0.846	2E-25	0.3			0	0		
Malignant Glioma	C0555198	CLCN3	1182	P51790	chloride voltage-gated channel 3	Ion channel	67	0.621	0.615	0.9997	0.3		1	1	0	2003	2003
Malignant Glioma	C0555198	BMI1	648	P35226	BMI1 proto-oncogene, polycomb ring finger		309	0.448	0.808	0.9429	0.3		1	1	0	2013	2013
Malignant Glioma	C0555198	MYLK	4638	Q15746	myosin light chain kinase	Kinase	199	0.51	0.846	9E-12	0.3		1	1	0	1999	1999
Malignant Glioma	C0555198	MYB	4602	P10242	MYB proto-oncogene, transcription factor		206	0.49	0.692	0.8192	0.3		1	2	0	2013	2016
Malignant Glioma	C0555198	MYBL1	4603	P10243	MYB proto-oncogene like 1		25	0.705	0.308	1	0.3		1	1	0	2013	2013
Malignant Glioma	C0555198	H3C2	8358	P68431	H3 clustered histone 2		43	0.653	0.423	1E-05	0.3		1	1	0	2012	2012

Malignant Glioma	C0555198	POT1	25913	Q9NUX5	protection of telomeres 1	Nucleic acid binding	107	0.56	0.731	0.8534	0.3			0	0		
Malignant Glioma	C0555198	HMGN5	79366	P82970	high mobility group nucleosome binding domain 5		42	0.65	0.423	0.3096	0.3		1	1	0	2012	2012
Malignant Glioma	C0555198	IL12A	3592	P29459	interleukin 12A		261	0.491	0.808	0.0475	0.3		1	1	0	2008	2008
Malignant Glioma	C0555198	SUZ12	23512	Q15022	SUZ12 polycomb repressive complex 2 subunit	Nucleic acid binding	165	0.529	0.577	1	0.3		1	1	0	2014	2014
Malignant Glioma	C0555198	XRCC1	7515	P18887	X-ray repair cross complementing 1		410	0.421	0.923	6E-09	0.3		1	1	0	2014	2014
Malignant Glioma	C0555198	XBP1	7494	P17861	X-box binding protein 1		234	0.477	0.846	0.032	0.3		1	1	0	2011	2011
Malignant Glioma	C0555198	PSD3	23362	Q9NYI0	pleckstrin and Sec7 domain containing 3		28	0.691	0.423	0.0095	0.3		1	1	0	2006	2006
Malignant Glioma	C0555198	GSTP1	2950	P09211	glutathione S-transferase pi 1		610	0.383	0.923	0.0142	0.3		1	1	0	2006	2006
Malignant Glioma	C0555198	QKI	9444	Q96PU8	QKI, KH domain containing RNA binding	Nucleic acid binding	61	0.617	0.5	0.7512	0.3		1	1	0	2016	2016
Malignant Glioma	C0555198	PHLDB1	23187	Q86UU1	pleckstrin homology like domain family B member 1		20	0.722	0.423	0.1067	0.3		1	1	0	2009	2009
Malignant Glioma	C0555198	TOP2A	7153	P11388	DNA topoisomerase II alpha		200	0.497	0.731	0.9909	0.3		1	1	0	2008	2008
Malignant Glioma	C0555198	HLA-B	3106	P01889	major histocompatibility complex, class I, B		706	0.379	0.885	4E-05	0.3		1	1	0	2003	2003
Malignant Glioma	C0555198	HLA-C	3107	P10321	major histocompatibility complex, class I, C		435	0.415	0.846	2E-06	0.3		1	1	0	2003	2003
Malignant Glioma	C0555198	APOD	347	P05090	apolipoprotein D		89	0.584	0.577	0.0005	0.3		1	1	0	2006	2006
Malignant Glioma	C0555198	IL12B	3593	P29460	interleukin 12B	Signaling	249	0.48	0.885	0.0001	0.3		1	1	0	2008	2008
Malignant Glioma	C0555198	GJA1	2697	P17302	gap junction protein alpha 1	Cell-cell junction	662	0.393	0.885	0.1552	0.26		1	7	0	2010	2019
Malignant Glioma	C0555198	MMP2	4313	P08253	matrix metallopeptidase 2	Enzyme	1021	0.333	0.923	0.8388	0.26		1	7	0	1999	2018
Malignant Glioma	C0555198	AKT2	208	P31751	AKT serine/threonine kinase 2	Kinase	264	0.474	0.769	0.6443	0.26		1	7	0	2005	2015
Malignant Glioma	C0555198	MET	4233	P08581	MET proto-oncogene, receptor tyrosine kinase	Kinase	594	0.38	0.846	0.97	0.22		1	3	0	2008	2015

Malignant Glioma	C0555198	TNC	3371	P24821	tenascin C		369	0.433	0.846	1E-07	0.22		1	3	0	2006	2019
Malignant Glioma	C0555198	CXCR3	2833	P49682	C-X-C motif chemokine receptor 3	G-protein coupled receptor	367	0.436	0.808	0.181	0.21		1	2	0	2006	2011
Malignant Glioma	C0555198	BCAN	63827	Q96GW7	brevican	Extracellular structure	25	0.7	0.269	2E-06	0.21		1	3	0	2001	2005
Malignant Glioma	C0555198	ST6GAL1	6480	P15907	ST6 beta-galactoside alpha-2,6-sialyltransferase 1		101	0.569	0.731	0.2341	0.21		1	1	0	2001	2001
Malignant Glioma	C0555198	ANGPT2	285	O15123	angiopoietin 2	Signaling	457	0.41	0.846	0.8321	0.21		1	2	0	2001	2008
Malignant Glioma	C0555198	IFNG	3458	P01579	interferon gamma		1519	0.288	0.962	0.4716	0.21		1	2	0	1999	2010
Malignant Glioma	C0555198	F2	2147	P00734	coagulation factor II, thrombin	Enzyme	490	0.415	0.885	0.0011	0.2		1	1	0	2005	2005
Malignant Glioma	C0555198	BAD	572	Q92934	BCL2 associated agonist of cell death		125	0.553	0.769	0.0046	0.2		1	1	0	2006	2006
Malignant Glioma	C0555198	MKI67	4288	P46013	marker of proliferation Ki-67		351	0.431	0.808	1E-28	0.2		1	1	0	2012	2012
Malignant Glioma	C0555198	SIRT2	22933	Q8IXJ6	sirtuin 2	Epigenetic regulator	172	0.51	0.769	2E-08	0.2		1	1	0	2013	2013
Malignant Glioma	C0555198	HMGB1	3146	P09429	high mobility group box 1	Nucleic acid binding	724	0.368	0.923	0.8204	0.2		1	1	0	2000	2000
Malignant Glioma	C0555198	BIRC5	332	O15392	baculoviral IAP repeat containing 5	Enzyme modulator	199	0.495	0.731	0.0559	0.2		1	1	0	2007	2007
Malignant Glioma	C0555198	AREG	374	P15514	amphiregulin	Signaling	221	0.487	0.769	0.3541	0.2		1	1	0	1996	1996
Malignant Glioma	C0555198	EPOR	2057	P19235	erythropoietin receptor	Receptor	162	0.526	0.692	0.0101	0.2		1	1	0	2011	2011
Malignant Glioma	C0555198	IL7	3574	P13232	interleukin 7		275	0.464	0.808	0.9257	0.2		1	1	0	1999	1999
Malignant Glioma	C0555198	PCMT1	5110	P22061	protein-L-isoaspartate (D-aspartate) O-methyltransferase	Enzyme	30	0.691	0.423	0.7845	0.2		1	1	0	2005	2005
Malignant Glioma	C0555198	LGALS3	3958	P17931	galectin 3	Signaling	557	0.392	0.846	2E-05	0.2		1	1	0	2013	2013
Malignant Glioma	C0555198	YEATS4	8089	O95619	YEATS domain containing 4	Nucleic acid binding	40	0.666	0.231	7E-05	0.2		1	1	0	2001	2001
Malignant Glioma	C0555198	CTSB	1508	P07858	cathepsin B	Enzyme	304	0.457	0.846	6E-11	0.2		1	1	0	2007	2007
Malignant Glioma	C0555198	SH3GL1	6455	Q99961	SH3 domain containing GRB2 like 1, endophilin A2		33	0.686	0.346	0.0117	0.2		1	1	0	2012	2012

Malignant Glioma	C0555198	CSF1	1435	P09603	colony stimulating factor 1		259	0.473	0.808	0.9963	0.2		1	1	0	2005	2005
Malignant Glioma	C0555198	SLC6A3	6531	Q01959	solute carrier family 6 member 3	Transporter	373	0.453	0.885	0.9976	0.2		1	1	0	2004	2004
Malignant Glioma	C0555198	BDKRB2	624	P30411	bradykinin receptor B2	G-protein coupled receptor	105	0.585	0.808	0.1186	0.2		1	1	0	2010	2010
Malignant Glioma	C0555198	SPHK1	8877	Q9NYA1	sphingosine kinase 1	Kinase	236	0.48	0.808	3E-07	0.2		1	1	0	2007	2007
Malignant Glioma	C0555198	CAV2	858	P51636	caveolin 2	Enzyme modulator	96	0.575	0.692	0.027	0.2		1	1	0	2012	2012
Malignant Glioma	C0555198	CAV1	857	Q03135	caveolin 1	Enzyme modulator	633	0.388	0.885	0.0083	0.2		1	1	0	2012	2012
Malignant Glioma	C0555198	ATG7	10533	O95352	autophagy related 7	Enzyme	179	0.505	0.769	7E-12	0.2		1	1	0	2015	2015
Malignant Glioma	C0555198	CD80	941	P33681	CD80 molecule		233	0.475	0.769	0.0109	0.2		1	1	0	1999	1999
Malignant Glioma	C0555198	ATM	472	Q13315	ATM serine/threonine kinase	Kinase	684	0.374	0.885	6E-47	0.11		1	1	1	1998	1998
Malignant Glioma	C0555198	EGFR	1956	P00533	epidermal growth factor receptor	Kinase	1394	0.295	0.885	0.3684	0.1		0.969	64	0	1987	2018
Malignant Glioma	C0555198	VEGFA	7422	P15692	vascular endothelial growth factor A	Signaling	1899	0.266	0.923	2E-05	0.1		1	22	0	2000	2018
Malignant Glioma	C0555198	IDH2	3418	P48735	isocitrate dehydrogenase (NADP(+)) 2		380	0.434	0.808	0.8827	0.1		1	10	0	2009	2019
Malignant Glioma	C0555198	GFAP	2670	P14136	glial fibrillary acidic protein		447	0.421	0.885	1E-06	0.1		1	12	0	1996	2019
Malignant Glioma	C0555198	EGF	1950	P01133	epidermal growth factor		774	0.357	0.923	9E-17	0.1		1	12	0	1985	2018
Malignant Glioma	C0555198	BCL2	596	P10415	BCL2 apoptosis regulator	Signaling	1456	0.291	0.885	0.559	0.1		1	12	0	1995	2015
Malignant Glioma	C0555198	C11orf65	160140	Q8NCR3	chromosome 11 open reading frame 65		45	0.67	0.462	2E-14	0.1			0	1		
Malignant Glioma	C0555198	IFNB1	3456	P01574	interferon beta 1		426	0.421	0.846		0.1		1	13	0	1992	2015
Malignant Glioma	C0555198	HIF1A	3091	Q16665	hypoxia inducible factor 1 subunit alpha	Transcription factor	1044	0.327	0.923	0.9777	0.09		1	9	0	2006	2019
Malignant Glioma	C0555198	H3P10	1.2E+08		H3 histone pseudogene 10		769	0.35	0.846		0.09		0.889	9	0	1996	2006
Malignant Glioma	C0555198	AKT1	207	P31749	AKT serine/threonine kinase 1	Kinase	1250	0.311	0.962	0.9759	0.07		1	7	0	2005	2019

Malignant Glioma	C0555198	PROM1	8842	O43490	prominin 1	Transporter	477	0.41	0.846	2E-22	0.07		1	7	0	2008	2019
Malignant Glioma	C0555198	STAT3	6774	P40763	signal transducer and activator of transcription 3	Nucleic acid binding	1193	0.32	0.923	1	0.07		1	7	0	2007	2017
Malignant Glioma	C0555198	PIK3CA	5290	P42336	phosphatidylinositol-4,5-bisphosphate 3-kinase catalytic subunit alpha	Kinase	1511	0.292	0.923	1	0.06		1	6	0	2002	2019
Malignant Glioma	C0555198	FAS	355	P25445	Fas cell surface death receptor		754	0.372	0.923	0.8117	0.06		1	6	0	1995	2018
Malignant Glioma	C0555198	PIK3CB	5291	P42338	phosphatidylinositol-4,5-bisphosphate 3-kinase catalytic subunit beta	Kinase	1083	0.322	0.885	0.9996	0.06		1	6	0	2002	2019
Malignant Glioma	C0555198	PIK3CD	5293	O00329	phosphatidylinositol-4,5-bisphosphate 3-kinase catalytic subunit delta	Kinase	1119	0.319	0.885	1	0.06		1	6	0	2002	2019
Malignant Glioma	C0555198	FABP7	2173	O15540	fatty acid binding protein 7		82	0.584	0.577	0.0008	0.06		1	6	0	2000	2013
Malignant Glioma	C0555198	MDM2	4193	Q00987	MDM2 proto-oncogene	Nucleic acid binding	702	0.362	0.846	0.9998	0.06		1	6	0	1993	2012
Malignant Glioma	C0555198	PIK3CG	5294	P48736	phosphatidylinositol-4,5-bisphosphate 3-kinase catalytic subunit gamma	Kinase	1101	0.32	0.885	1E-06	0.06		1	6	0	2002	2019
Malignant Glioma	C0555198	MIR221	407006		microRNA 221		304	0.449	0.846		0.05		0.8	5	0	2010	2019
Malignant Glioma	C0555198	OLIG2	10215	Q13516	oligodendrocyte transcription factor 2	Enzyme	104	0.56	0.577	0.0946	0.05		1	5	0	2006	2017
Malignant Glioma	C0555198	FASLG	356	P48023	Fas ligand	Signaling	398	0.43	0.885	0.1835	0.05		1	5	0	1998	2002
Malignant Glioma	C0555198	LOC110806263	1.1E+08		TERT 5' regulatory region		245	0.464	0.731		0.05		1	5	0	2013	2019
Malignant Glioma	C0555198	IL13	3596	P35225	interleukin 13		587	0.386	0.846	0.0144	0.05		1	5	0	1999	2008
Malignant Glioma	C0555198	TGFB1	7040	P01137	transforming growth factor beta 1	Signaling	1558	0.287	0.962	0.0369	0.05		1	5	0	1991	2007
Malignant Glioma	C0555198	NES	10763	P48681	nestin		302	0.45	0.808	2E-06	0.05		1	5	0	2005	2019
Malignant Glioma	C0555198	SOX2	6657	P48431	SRY-box transcription factor 2	Transcription factor	503	0.405	0.808	0.7066	0.05		1	5	0	2007	2019
Malignant Glioma	C0555198	PDGFRA	5156	P16234	platelet derived growth factor receptor alpha	Kinase	452	0.415	0.808	1	0.05		1	5	0	1992	2019

Malignant Glioma	C0555198	CTNNB1	1499	P35222	catenin beta 1		1368	0.303	0.885	1	0.05		1	5	0	2011	2017
Malignant Glioma	C0555198	FOXM1	2305	Q08050	forkhead box M1		323	0.441	0.769	0.0007	0.04		1	4	0	2006	2019
Malignant Glioma	C0555198	ATF5	22809	Q9Y2D1	activating transcription factor 5		41	0.674	0.385	0.2916	0.04		1	4	0	2010	2017
Malignant Glioma	C0555198	CDK2	1017	P24941	cyclin dependent kinase 2	Kinase	270	0.456	0.692	0.6105	0.04		1	4	0	1994	2004
Malignant Glioma	C0555198	TGFB2	7042	P61812	transforming growth factor beta 2	Signaling	389	0.433	0.885	0.9991	0.04		1	4	0	1991	2016
Malignant Glioma	C0555198	IL4R	3566	P24394	interleukin 4 receptor	Receptor	242	0.474	0.846	0.0002	0.04		1	4	2	2000	2010
Malignant Glioma	C0555198	SND1	27044	Q7KZF4	staphylococcal nuclease and tudor domain containing 1	Transcription factor	101	0.576	0.769	0.9821	0.04		1	4	0	2015	2018
Malignant Glioma	C0555198	TERC	7012		telomerase RNA component		296	0.471	0.846		0.04		1	4	0	2000	2015
Malignant Glioma	C0555198	EGR1	1958	P18146	early growth response 1	Nucleic acid binding	378	0.433	0.923	0.3509	0.04		1	4	0	1998	2017
Malignant Glioma	C0555198	CD44	960	P16070	CD44 molecule (Indian blood group)		711	0.363	0.962	2E-07	0.04		1	4	0	1997	2017
Malignant Glioma	C0555198	MIR21	406991		microRNA 21		726	0.363	0.846		0.04		1	4	0	2012	2017
Malignant Glioma	C0555198	MAPK1	5594	P28482	mitogen-activated protein kinase 1	Kinase	1059	0.33	0.923	0.997	0.04		1	4	0	2010	2017
Malignant Glioma	C0555198	CDK4	1019	P11802	cyclin dependent kinase 4	Kinase	433	0.412	0.808	0.0629	0.03		1	3	0	1996	2011
Malignant Glioma	C0555198	MMP9	4318	P14780	matrix metallopeptidase 9	Enzyme	1337	0.305	0.923	2E-17	0.03		1	3	0	1996	2010
Malignant Glioma	C0555198	IL1B	3553	P01584	interleukin 1 beta		1801	0.276	0.962	0.1301	0.03		1	3	0	2014	2019
Malignant Glioma	C0555198	WT1	7490	P19544	WT1 transcription factor	Nucleic acid binding	446	0.422	0.808	0.9965	0.03		1	3	0	2007	2012
Malignant Glioma	C0555198	MMP14	4323	P50281	matrix metallopeptidase 14	Enzyme	340	0.447	0.769	0.9952	0.03		1	3	0	1999	2000
Malignant Glioma	C0555198	MBP	4155	P02686	myelin basic protein		184	0.51	0.885	0.2845	0.03		1	3	0	1997	2000
Malignant Glioma	C0555198	IFNA13	3447	P01562	interferon alpha 13		646	0.374	0.923		0.03		1	3	0	1993	2015
Malignant Glioma	C0555198	MIR146B	574447		microRNA 146b		187	0.496	0.769		0.03		1	3	0	2013	2016
Malignant Glioma	C0555198	ZFP36	7538	P26651	ZFP36 ring finger protein	Nucleic acid binding	161	0.523	0.692	0.7524	0.03		1	3	0	2008	2015
Malignant Glioma	C0555198	MDM4	4194	O15151	MDM4 regulator of p53	Nucleic acid binding	265	0.461	0.808	0.9999	0.03		1	3	0	1999	2003

Malignant Glioma	C0555198	EGFL7	51162	Q9UHF1	EGF like domain multiple 7	Calcium-binding protein	68	0.612	0.577	6E-18	0.03		1	3	0	2010	2018
Malignant Glioma	C0555198	DSC3	1825	Q14574	desmocollin 3	Cell adhesion	103	0.57	0.808	2E-21	0.03		1	3	0	2017	2017
Malignant Glioma	C0555198	CCL2	6347	P13500	C-C motif chemokine ligand 2	Signaling	1157	0.321	0.962	0.6079	0.03		1	3	0	1994	2012
Malignant Glioma	C0555198	IFNA1	3439	P01562	interferon alpha 1		662	0.371	0.923		0.03		1	3	0	1993	2015
Malignant Glioma	C0555198	BDNF	627	P23560	brain derived neurotrophic factor	Signaling	992	0.345	0.923	0.6563	0.03		1	3	0	1993	2019
Malignant Glioma	C0555198	DCN	1634	P07585	decorin		297	0.457	0.808	0.3123	0.03		1	3	0	1998	1999
Malignant Glioma	C0555198	KDR	3791	P35968	kinase insert domain receptor	Kinase	623	0.378	0.885	0.9998	0.03		1	3	0	2006	2018
Malignant Glioma	C0555198	EPHB2	2048	P29323	EPH receptor B2	Kinase	649	0.374	0.846	1	0.03		1	3	0	2010	2017
Malignant Glioma	C0555198	CCND1	595	P24385	cyclin D1	Enzyme modulator	859	0.344	0.923	0.8906	0.03		1	3	0	1999	2011
Malignant Glioma	C0555198	CASP8	841	Q14790	caspase 8	Enzyme	480	0.404	0.923	4E-06	0.03		1	3	0	2002	2017
Malignant Glioma	C0555198	TIMP2	7077	P16035	TIMP metallopeptidase inhibitor 2	Enzyme modulator	404	0.425	0.846	0.7889	0.03		1	3	0	2000	2004
Malignant Glioma	C0555198	TGFA	7039	P01135	transforming growth factor alpha	Signaling	376	0.432	0.885	0.7198	0.03		1	3	0	1988	1999
Malignant Glioma	C0555198	WWTR1	25937	Q9GZV5	WW domain containing transcription regulator 1	Enzyme modulator	134	0.529	0.615	0.9391	0.03		1	3	0	2011	2019
Malignant Glioma	C0555198	LRIG1	26018	Q96JA1	leucine rich repeats and immunoglobulin like domains 1	Receptor	83	0.601	0.538	0.035	0.03		1	3	0	2009	2018
Malignant Glioma	C0555198	MAPK14	1432	Q16539	mitogen-activated protein kinase 14	Kinase	626	0.379	0.923	0.3747	0.03		1	3	0	2007	2013
Malignant Glioma	C0555198	ABCB1	5243	P08183	ATP binding cassette subfamily B member 1	Transporter	933	0.344	0.885	1E-05	0.03		1	3	0	1995	2005
Malignant Glioma	C0555198	THBS1	7057	P07996	thrombospondin 1		480	0.407	0.885	0.9999	0.03		1	3	0	2000	2004
Malignant Glioma	C0555198	ZHX2	22882	Q9Y6X8	zinc fingers and homeoboxes 2	Transcription factor	172	0.51	0.654	0.0337	0.03		1	3	0	2006	2010
Malignant Glioma	C0555198	EFEMP1	2202	Q12805	EGF containing fibulin extracellular matrix protein 1	Extracellular structure	153	0.54	0.769	0.9999	0.03		1	3	0	2011	2017

Malignant Glioma	C0555198	CYP2B6	1555	P20813	cytochrome P450 family 2 subfamily B member 6		388	0.428	0.846	2E-10	0.03		1	3	0	1996	2004
Malignant Glioma	C0555198	ANXA2	302	P07355	annexin A2		294	0.453	0.808	1E-04	0.02		1	2	0	1992	2015
Malignant Glioma	C0555198	TICAM2	353376	Q86XR7;Q9Y3B3	toll like receptor adaptor molecule 2		332	0.438	0.769	0.0026	0.02		1	2	0	1999	2004
Malignant Glioma	C0555198	IL6	3569	P05231	interleukin 6		2367	0.248	0.962	0.3154	0.02		1	2	0	2004	2012
Malignant Glioma	C0555198	CASP6	839	P55212	caspase 6	Enzyme	73	0.61	0.615	0.0001	0.02		1	2	0	2001	2004
Malignant Glioma	C0555198	IL4	3565	P05112	interleukin 4		996	0.332	0.962	0.0047	0.02		1	2	0	2005	2007
Malignant Glioma	C0555198	TRIM11	81559	Q96F44	tripartite motif containing 11		31	0.686	0.385	0.9173	0.02		1	2	0	2013	2016
Malignant Glioma	C0555198	IGFBP2	3485	P18065	insulin like growth factor binding protein 2	Enzyme modulator	241	0.478	0.769	0.119	0.02		1	2	0	2011	2016
Malignant Glioma	C0555198	MTDH	92140	Q86UE4	metadherin		200	0.491	0.769	0.3292	0.02		1	2	0	2008	2018
Malignant Glioma	C0555198	AURKB	9212	Q96GD4	aurora kinase B	Kinase	161	0.51	0.769	0.0045	0.02		1	2	0	2009	2012
Malignant Glioma	C0555198	HGF	3082	P14210	hepatocyte growth factor	Enzyme	671	0.374	0.885	0.9995	0.02		1	2	0	2001	2007
Malignant Glioma	C0555198	LGI1	9211	O95970	leucine rich glioma inactivated 1		118	0.568	0.731	0.9991	0.02		1	2	0	1998	2005
Malignant Glioma	C0555198	CD2	914	P06729	CD2 molecule		50	0.641	0.577	0.4711	0.02		1	2	0	2017	2018
Malignant Glioma	C0555198	XIAP	331	P98170	X-linked inhibitor of apoptosis	Enzyme modulator	321	0.447	0.808	0.9184	0.02		1	2	0	1999	2010
Malignant Glioma	C0555198	ICAM1	3383	P05362	intercellular adhesion molecule 1		737	0.364	0.962	0.0353	0.02		1	2	0	1994	2015
Malignant Glioma	C0555198	GADL1	339896	Q6ZQY3	glutamate decarboxylase like 1		192	0.497	0.808	1E-21	0.02		1	2	0	2018	2019
Malignant Glioma	C0555198	TAX1BP3	30851	O14907	Tax1 binding protein 3		27	0.716	0.615	0.0052	0.02		1	2	0	2012	2014
Malignant Glioma	C0555198	HAVCR2	84868	Q8TDQ0	hepatitis A virus cellular receptor 2		299	0.45	0.808	0.0387	0.02		1	2	0	2017	2020
Malignant Glioma	C0555198	IFI27	3429	P40305	interferon alpha inducible protein 27		332	0.437	0.769	0.0107	0.02		1	2	0	1999	2004
Malignant Glioma	C0555198	CFLAR	8837	O15519	CASP8 and FADD like apoptosis regulator	Enzyme	215	0.485	0.846	0.9997	0.02		1	2	0	2008	2009
Malignant Glioma	C0555198	FADD	8772	Q13158	Fas associated via death domain		137	0.537	0.846	0.526	0.02		1	2	0	1998	2001
Malignant Glioma	C0555198	KIF23	9493	Q02241	kinesin family member 23	Cellular structure	51	0.682	0.385	1	0.02		1	2	0	2012	2016

Malignant Glioma	C0555198	ROCK1	6093	Q13464	Rho associated coiled-coil containing protein kinase 1	Kinase	157	0.513	0.731	1	0.02		1	2	0	2010	2015
Malignant Glioma	C0555198	PCNA	5111	P12004	proliferating cell nuclear antigen	Nucleic acid binding	581	0.382	0.846	0.9761	0.02		1	2	0	2001	2011
Malignant Glioma	C0555198	TMED7	51014	Q86XR7;Q9Y3B3	transmembrane p24 trafficking protein 7	Transporter	336	0.436	0.808	0.4797	0.02		1	2	0	1999	2004
Malignant Glioma	C0555198	SOX11	6664	P35716	SRY-box transcription factor 11	Transcription factor	246	0.494	0.808	0.8584	0.02		1	2	0	2005	2013
Malignant Glioma	C0555198	MIR584	693169		microRNA 584		42	0.65	0.385		0.02		1	2	0	2016	2019
Malignant Glioma	C0555198	TEK	7010	Q02763	TEK receptor tyrosine kinase	Kinase	300	0.462	0.692	1	0.02		1	2	0	2006	2009
Malignant Glioma	C0555198	NFE2L2	4780	Q16236	nuclear factor, erythroid 2 like 2	Enzyme	823	0.357	0.885	0.0036	0.02		1	2	0	2015	2015
Malignant Glioma	C0555198	MSI1	4440	O43347	musashi RNA binding protein 1		116	0.55	0.615	0.9936	0.02		1	2	0	2007	2020
Malignant Glioma	C0555198	MSH2	4436	P43246	mutS homolog 2	Nucleic acid binding	490	0.406	0.808	0.8954	0.02		1	2	0	2004	2007
Malignant Glioma	C0555198	SATB1	6304	Q01826	SATB homeobox 1	Transcription factor	147	0.525	0.769	0.9905	0.02		0.5	2	0	2013	2018
Malignant Glioma	C0555198	PDCD1	5133	Q15116	programmed cell death 1		497	0.402	0.846	0.4171	0.02		1	2	0	2017	2017
Malignant Glioma	C0555198	PVR	5817	P15151	PVR cell adhesion molecule		177	0.51	0.769	4E-10	0.02		1	2	0	2012	2017
Malignant Glioma	C0555198	PSMD9	5715	O00233	proteasome 26S subunit, non-ATPase 9	Enzyme modulator	344	0.435	0.846	5E-06	0.02		1	2	0	1999	2004
Malignant Glioma	C0555198	AJAP1	55966	Q9UKB5	adherens junctions associated protein 1		26	0.722	0.192	0.9947	0.02		1	2	0	2014	2014
Malignant Glioma	C0555198	TRPM7	54822	Q96QT4	transient receptor potential cation channel subfamily M member 7	Ion channel	151	0.531	0.731	5E-16	0.02		1	2	0	2015	2016
Malignant Glioma	C0555198	GOLPH3	64083	Q9H4A6	golgi phosphoprotein 3		140	0.53	0.808	0.2359	0.02		1	2	0	2015	2016
Malignant Glioma	C0555198	PLG	5340	P00747	plasminogen	Enzyme	586	0.389	0.923	0.0103	0.02		1	2	0	1999	2007
Malignant Glioma	C0555198	ADA2	51816	Q9NZK5	adenosine deaminase 2	Enzyme	165	0.545	0.846	8E-08	0.02		1	2	0	2017	2017
Malignant Glioma	C0555198	SERPINF1	5176	P36955	serpin family F member 1	Enzyme modulator	294	0.46	0.846	8E-08	0.02		1	2	0	2003	2004
Malignant Glioma	C0555198	XRCC6P5	442459		X-ray repair cross complementing 6 pseudogene 5		84	0.579	0.769		0.02		1	2	0	2003	2006

Malignant Glioma	C0555198	THY1	7070	P04216	Thy-1 cell surface antigen		197	0.496	0.808	0.0499	0.02		1	2	0	2013	2017
Malignant Glioma	C0555198	MMP1	4312	P03956	matrix metallopeptidase 1	Enzyme	589	0.385	0.885	8E-18	0.02		1	2	0	2005	2010
Malignant Glioma	C0555198	MIR148A	406940		microRNA 148a		172	0.506	0.846		0.02		1	2	0	2014	2017
Malignant Glioma	C0555198	MIR145	406937		microRNA 145		366	0.431	0.846		0.02		1	2	0	2012	2017
Malignant Glioma	C0555198	MIR126	406913		microRNA 126		305	0.447	0.846		0.02		1	2	0	2017	2018
Malignant Glioma	C0555198	LIG4	3981	P49917	DNA ligase 4		293	0.478	0.846	4E-06	0.02		1	2	0	2005	2007
Malignant Glioma	C0555198	CXCR4	7852	P61073	C-X-C motif chemokine receptor 4	G-protein coupled receptor	739	0.362	0.923	0.0178	0.02		1	2	0	2011	2018
Malignant Glioma	C0555198	LGALS1	3956	P09382	galectin 1	Signaling	362	0.435	0.846	0.0045	0.02		1	2	0	2007	2019
Malignant Glioma	C0555198	TFPI2	7980	P48307	tissue factor pathway inhibitor 2	Enzyme modulator	161	0.521	0.769	0.0008	0.02		1	2	0	2006	2011
Malignant Glioma	C0555198	CALCR	799	P30988	calcitonin receptor	G-protein coupled receptor	282	0.464	0.846	3E-09	0.02		1	2	0	2017	2019
Malignant Glioma	C0555198	MIR182	406958		microRNA 182		211	0.486	0.846		0.02		1	2	0	2010	2016
Malignant Glioma	C0555198	MIR200B	406984		microRNA 200b		180	0.5	0.769		0.02		1	2	0	2013	2016
Malignant Glioma	C0555198	TSPAN8	7103	P19075	tetraspanin 8		63	0.617	0.577	0.0055	0.02		1	2	0	2015	2015
Malignant Glioma	C0555198	MYC	4609	P01106	MYC proto-oncogene, bHLH transcription factor	Transcription factor	821	0.344	0.923	0.998	0.02		1	2	0	2017	2017
Malignant Glioma	C0555198	AKT3	10000	Q9Y243	AKT serine/threonine kinase 3	Kinase	183	0.512	0.808	0.9996	0.02		1	2	0	2010	2015
Malignant Glioma	C0555198	TPO	7173	P07202	thyroid peroxidase	Enzyme	306	0.455	0.846	2E-21	0.02		1	2	0	2011	2011
Malignant Glioma	C0555198	MCL1	4170	Q07820	MCL1 apoptosis regulator, BCL2 family member	Signaling	375	0.43	0.808	0.9635	0.02		1	2	0	2010	2011
Malignant Glioma	C0555198	MIR29A	407021		microRNA 29a		278	0.459	0.808		0.02		1	2	0	2017	2018
Malignant Glioma	C0555198	MIR215	406997		microRNA 215		96	0.559	0.654		0.02		1	2	0	2017	2017
Malignant Glioma	C0555198	CA9	768	Q16790	carbonic anhydrase 9		226	0.483	0.731	1E-09	0.02		1	2	0	2007	2019
Malignant Glioma	C0555198	AQP4	361	P55087	aquaporin 4	Ion channel	311	0.456	0.885	0.0023	0.02		1	2	0	2017	2019

Malignant Glioma	C0555198	CREB1	1385	P16220	cAMP responsive element binding protein 1		294	0.463	0.885	0.9967	0.02		1	2	0	2013	2015
Malignant Glioma	C0555198	IL24	11009	Q13007	interleukin 24		202	0.498	0.769	6E-07	0.02		1	2	0	2003	2010
Malignant Glioma	C0555198	ZNRD2	10534	O60232	zinc ribbon domain containing 2		311	0.443	0.769	2E-07	0.02		1	2	0	1999	2004
Malignant Glioma	C0555198	E2F1	1869	Q01094	E2F transcription factor 1	Nucleic acid binding	324	0.441	0.846	0.9877	0.02		1	2	0	2005	2011
Malignant Glioma	C0555198	ALDH1A3	220	P47895	aldehyde dehydrogenase 1 family member A3	Enzyme	83	0.578	0.654	0.1413	0.02		1	2	0	2013	2018
Malignant Glioma	C0555198	GOT2	2806	P00505	glutamic-oxaloacetic transaminase 2		148	0.529	0.731	0.0142	0.02		1	2	0	2010	2013
Malignant Glioma	C0555198	TMED7-TICAM2	1E+08	Q86XR7;Q9Y3B3	TMED7-TICAM2 readthrough		329	0.438	0.769	0.0026	0.02		1	2	0	1999	2004
Malignant Glioma	C0555198	AZIN2	113451	Q96A70	antizyme inhibitor 2	Enzyme	187	0.499	0.808	9E-07	0.02		1	2	0	2018	2019
Malignant Glioma	C0555198	HPGDS	27306	O60760	hematopoietic prostaglandin D synthase		570	0.388	0.923	2E-06	0.02		1	2	0	2000	2004
Malignant Glioma	C0555198	PLCB1	23236	Q9NQ66	phospholipase C beta 1	Enzyme	107	0.595	0.654	0.9834	0.02		1	2	0	2016	2019
Malignant Glioma	C0555198	GAST	2520	P01350	gastrin		236	0.485	0.808	0.0027	0.02		1	2	0	2001	2003
Malignant Glioma	C0555198	CSF2	1437	P04141	colony stimulating factor 2	Signaling	1028	0.33	0.962	0.8347	0.02		1	2	0	2006	2017
Malignant Glioma	C0555198	LAT	27040	O43561	linker for activation of T cells		124	0.548	0.731	0.0092	0.02		1	2	0	2013	2018
Malignant Glioma	C0555198	EZH2	2146	Q15910	enhancer of zeste 2 polycomb repressive complex 2 subunit	Epigenetic regulator	653	0.376	0.885	1	0.02		1	2	0	2011	2012
Malignant Glioma	C0555198	EPAS1	2034	Q99814	endothelial PAS domain protein 1	Transcription factor	293	0.454	0.731	0.5961	0.02		1	2	0	2014	2017
Malignant Glioma	C0555198	CCNO	10309	P22674	cyclin O	Enzyme modulator	67	0.621	0.538	0.0035	0.02		1	2	0	2014	2018
Malignant Glioma	C0555198	H3P23	1.2E+08		H3 histone pseudogene 23		310	0.443	0.769		0.02		1	2	0	1999	2004
Malignant Glioma	C0555198	SLC38A3	10991	Q99624	solute carrier family 38 member 3	Transporter	25	0.711	0.5		0.02		1	2	0	2004	2006
Malignant Glioma	C0555198	CYP27B1	1594	O15528	cytochrome P450 family 27 subfamily B member 1	Enzyme	229	0.502	0.769	2E-07	0.02		1	2	0	2001	2004

Malignant Glioma	C0555198	DCTN6	10671	O00399	dynactin subunit 6	Cellular structure	315	0.442	0.769	0.0557	0.02		1	2	0	1999	2004
Malignant Glioma	C0555198	MSH6	2956	P52701	mutS homolog 6	Nucleic acid binding	296	0.462	0.731	4E-05	0.02		1	2	0	2006	2013
Malignant Glioma	C0555198	LILRB1	10859	Q8NHL6	leukocyte immunoglobulin like receptor B1	Receptor	168	0.51	0.769	6E-14	0.02		1	2	0	2011	2014
Malignant Glioma	C0555198	DKK1	22943	O94907	dickkopf WNT signaling pathway inhibitor 1		372	0.439	0.885	0.1742	0.02		1	2	0	2014	2014
Malignant Glioma	C0555198	GADD45A	1647	P24522	growth arrest and DNA damage inducible alpha		151	0.526	0.769	0.0441	0.02		1	2	0	2010	2017
Malignant Glioma	C0555198	CD274	29126	Q9NZQ7	CD274 molecule	Receptor	1011	0.324	0.923	0.0192	0.02		1	2	0	2003	2018
Malignant Glioma	C0555198	DMBT1	1755	Q9UGM3	deleted in malignant brain tumors 1	Enzyme	135	0.533	0.654	4E-63	0.02		1	2	0	2001	2002
Malignant Glioma	C0555198	GSTT1	2952	P30711	glutathione S-transferase theta 1		541	0.393	0.923	0.0001	0.02		1	2	0	2006	2013
Malignant Glioma	C0555198	CXADR	1525	P78310	CXADR Ig-like cell adhesion molecule		305	0.454	0.885	0.3687	0.02		1	2	0	2007	2007
Malignant Glioma	C0555198	SOX9	6662	P48436	SRY-box transcription factor 9		466	0.419	0.846	0.9977	0.01		1	1	0	2015	2015
Malignant Glioma	C0555198	ADAM17	6868	P78536	ADAM metallopeptidase domain 17		316	0.453	0.808	0.9953	0.01		1	1	0	2017	2017
Malignant Glioma	C0555198	TSC1	7248	Q92574	TSC complex subunit 1		391	0.44	0.808	1	0.01		1	1	0	2016	2016
Malignant Glioma	C0555198	CNTN2	6900	Q02246	contactin 2	Receptor	99	0.581	0.692	0.0021	0.01		1	1	0	2003	2003
Malignant Glioma	C0555198	TRP-AGG2-6	7218		tRNA-Pro (anticodon AGG) 2-6		34	0.666	0.462		0.01		1	1	0	2004	2004
Malignant Glioma	C0555198	TREX1	11277	Q9NSU2	three prime repair exonuclease 1		241	0.517	0.808	0.5753	0.01		1	1	0	2013	2013
Malignant Glioma	C0555198	TAZ	6901	Q16635	tafazzin	Enzyme	252	0.477	0.808	0.7259	0.01		1	1	0	2011	2011
Malignant Glioma	C0555198	CD109	135228	Q6YHK3	CD109 molecule		81	0.595	0.654	2E-44	0.01		1	1	0	2018	2018
Malignant Glioma	C0555198	TCF4	6925	P15884	transcription factor 4	Transcription factor	378	0.464	0.846	0.9998	0.01		1	1	0	2011	2011
Malignant Glioma	C0555198	BSG	682	P35613	basigin (Ok blood group)		287	0.458	0.769	3E-05	0.01		1	1	0	2000	2000
Malignant Glioma	C0555198	STK11	6794	Q15831	serine/threonine kinase 11	Kinase	372	0.435	0.808	0.9934	0.01		1	1	0	2017	2017

Malignant Glioma	C0555198	SPG7	6687	Q9UQ90	SPG7 matrix AAA peptidase subunit, paraplegin	Enzyme	419	0.436	0.885	2E-36	0.01		1	1	0	2007	2007
Malignant Glioma	C0555198	TTR	7276	P02766	transthyretin	Transporter	461	0.423	0.885	0.5157	0.01		1	1	0	2001	2001
Malignant Glioma	C0555198	SPARC	6678	P09486	secreted protein acidic and cysteine rich	Signaling	344	0.445	0.846	0.8915	0.01		1	1	0	2009	2009
Malignant Glioma	C0555198	UHRF2	115426	Q96PU4	ubiquitin like with PHD and ring finger domains 2	Enzyme	33	0.7	0.308	0.9993	0.01		1	1	0	2012	2012
Malignant Glioma	C0555198	SST	6750	P61278	somatostatin	Signaling	535	0.399	0.885	0.3413	0.01		1	1	0	2004	2004
Malignant Glioma	C0555198	FBXO17	115290	Q96EF6	F-box protein 17		11	0.839	0.115	6E-13	0.01		1	1	0	2018	2018
Malignant Glioma	C0555198	SP1	6667	P08047	Sp1 transcription factor	Nucleic acid binding	209	0.493	0.769	1	0.01		1	1	0	2009	2009
Malignant Glioma	C0555198	STC1	6781	P52823	stanniocalcin 1	Signaling	137	0.532	0.731	0.2036	0.01		1	1	0	2015	2015
Malignant Glioma	C0555198	COX8A	1351	P10176	cytochrome c oxidase subunit 8A	Enzyme	526	0.4	0.846	0.1161	0.01		1	1	0	2017	2017
Malignant Glioma	C0555198	SLCO6A1	133482	Q86UG4	solute carrier organic anion transporter family member 6A1	Transporter	449	0.412	0.885	3E-11	0.01		1	1	0	2005	2005
Malignant Glioma	C0555198	TRAF6	7189	Q9Y4K3	TNF receptor associated factor 6	Signaling	254	0.472	0.808	0.9974	0.01		1	1	0	2015	2015
Malignant Glioma	C0555198	HHLA2	11148	Q9UM44	HERV-H LTR-associating 2	Enzyme modulator	44	0.653	0.423	2E-06	0.01		1	1	0	2019	2019
Malignant Glioma	C0555198	MNS16A	1.1E+08		MNS16A minisatellite promoter		35	0.663	0.5		0.01		1	1	0	2007	2007
Malignant Glioma	C0555198	TGFBI	7045	Q15582	transforming growth factor beta induced	Signaling	225	0.484	0.769	6E-08	0.01		1	1	0	2018	2018
Malignant Glioma	C0555198	NUDT21	11051	O43809	nudix hydrolase 21	Nucleic acid binding	32	0.682	0.231	0.9858	0.01		1	1	0	2019	2019
Malignant Glioma	C0555198	TSPO	706	P30536	translocator protein		341	0.444	0.885	0.0003	0.01		1	1	0	2019	2019
Malignant Glioma	C0555198	GIPC1	10755	O14908	GIPC PDZ domain containing family member 1	Enzyme	54	0.628	0.577	0.0083	0.01		1	1	0	2018	2018
Malignant Glioma	C0555198	TIMP1	7076	P01033	TIMP metallopeptidase inhibitor 1	Enzyme modulator	603	0.38	0.885	0.4993	0.01		1	1	0	2000	2000
Malignant Glioma	C0555198	SLC27A3	11000	Q5K4L6	solute carrier family 27 member 3	Transporter	16	0.769	0.115	5E-22	0.01		1	1	0	2009	2009

Malignant Glioma	C0555198	TK1	7083	P04183	thymidine kinase 1	Kinase	82	0.592	0.538	0.1373	0.01		1	1	0	2010	2010
Malignant Glioma	C0555198	C1QL1	10882	O75973	complement C1q like 1		73	0.612	0.654	6E-06	0.01		1	1	0	2012	2012
Malignant Glioma	C0555198	PSIP1	11168	O75475	PC4 and SFRS1 interacting protein 1	Signaling	210	0.49	0.846	0.9967	0.01		1	1	0	2013	2013
Malignant Glioma	C0555198	PRAF2	11230	O60831	PRA1 domain family member 2		15	0.78	0.269	0.7424	0.01		1	1	0	2010	2010
Malignant Glioma	C0555198	TCF3	6929	P15923	transcription factor 3	Transcription factor	244	0.476	0.846	0.0258	0.01		1	1	0	2013	2013
Malignant Glioma	C0555198	TPM3	7170	P06753	tropomyosin 3	Cellular structure	297	0.49	0.808	0.0067	0.01		1	1	0	2018	2018
Malignant Glioma	C0555198	MIR608	693193		microRNA 608		55	0.615	0.538		0.01		1	1	0	2016	2016
Malignant Glioma	C0555198	TCF7L2	6934	Q9NQB0	transcription factor 7 like 2		257	0.485	0.846	0.9954	0.01		1	1	0	2011	2011
Malignant Glioma	C0555198	TRA	6955	P0DSE1	T cell receptor alpha locus		51	0.628	0.577		0.01		1	1	0	2004	2004
Malignant Glioma	C0555198	KLF8	11279	O95600	Kruppel like factor 8	Nucleic acid binding	55	0.628	0.462	0.0022	0.01		1	1	0	2012	2012
Malignant Glioma	C0555198	CYTOR	112597		cytoskeleton regulator RNA		90	0.568	0.577		0.01		1	1	0	2018	2018
Malignant Glioma	C0555198	GJB6	10804	O95452	gap junction protein beta 6	Cell-cell junction	176	0.528	0.808	2E-07	0.01		1	1	0	2015	2015
Malignant Glioma	C0555198	TFF3	7033	Q07654	trefoil factor 3	Signaling	163	0.518	0.885	0.0159	0.01		1	1	0	2017	2017
Malignant Glioma	C0555198	TNFRSF1B	7133	P20333	TNF receptor superfamily member 1B		417	0.425	0.885	0.502	0.01		1	1	0	2013	2013
Malignant Glioma	C0555198	TMSB4X	7114	P62328	thymosin beta 4 X-linked		130	0.541	0.692	0.4121	0.01		0	1	0	2014	2014
Malignant Glioma	C0555198	MIR363	574031		microRNA 363		83	0.578	0.692		0.01		1	1	0	2016	2016
Malignant Glioma	C0555198	RAP1A	5906	P62834	RAP1A, member of RAS oncogene family	Enzyme modulator	215	0.502	0.808	0.6223	0.01		1	1	0	2013	2013
Malignant Glioma	C0555198	RB1	5925	P06400	RB transcriptional corepressor 1	Nucleic acid binding	339	0.444	0.885	1	0.01		1	1	0	2017	2017
Malignant Glioma	C0555198	CBLL2	158506	Q8N7E2	Cbl proto-oncogene like 2	Enzyme	235	0.476	0.808		0.01		1	1	0	2016	2016
Malignant Glioma	C0555198	CYP4B1	1580	P13584	cytochrome P450 family 4 subfamily B member 1	Enzyme	35	0.691	0.308	3E-10	0.01		1	1	0	1998	1998
Malignant Glioma	C0555198	ACTB	60	P60709	actin beta	Cellular structure	1110	0.325	0.923	0.9856	0.01		1	1	0	2011	2011
Malignant Glioma	C0555198	BCL6	604	P41182	BCL6 transcription repressor		309	0.444	0.808	0.9604	0.01		1	1	0	2017	2017

Malignant Glioma	C0555198	GAS5	60674		growth arrest specific 5		184	0.497	0.808		0.01		1	1	0	2019	2019
Malignant Glioma	C0555198	ROBO1	6091	Q9Y6N7	roundabout guidance receptor 1		144	0.535	0.731	7E-16	0.01		1	1	0	2009	2009
Malignant Glioma	C0555198	BAG1	573	Q99933	BAG cochaperone 1		128	0.538	0.769	7E-06	0.01		1	1	0	2000	2000
Malignant Glioma	C0555198	CUX1	1523	P39880;Q13948	cut like homeobox 1	Transcription factor	283	0.465	0.846	1	0.01		1	1	0	2013	2013
Malignant Glioma	C0555198	CTSZ	1522	Q9UBR2	cathepsin Z	Enzyme	51	0.647	0.577	2E-16	0.01		1	1	0	2018	2018
Malignant Glioma	C0555198	CTSK	1513	P43235	cathepsin K	Enzyme	221	0.497	0.808	3E-05	0.01		1	1	0	2018	2018
Malignant Glioma	C0555198	RANBP2	5903	P49792	RAN binding protein 2	Enzyme modulator	110	0.578	0.615	1	0.01		1	1	0	2004	2004
Malignant Glioma	C0555198	ADAM3A	1587		ADAM metallopeptidase domain 3A (pseudogene)		11	0.792	0.231		0.01		1	1	0	2011	2011
Malignant Glioma	C0555198	RAC1	5879	P63000	Rac family small GTPase 1	Enzyme modulator	415	0.429	0.962	0.7623	0.01		1	1	0	2002	2002
Malignant Glioma	C0555198	DCC	1630	P43146	DCC netrin 1 receptor		279	0.471	0.808	0.9945	0.01		1	1	0	1993	1993
Malignant Glioma	C0555198	DAXX	1616	Q9UER7	death domain associated protein		137	0.544	0.692	0.0986	0.01		1	1	0	2019	2019
Malignant Glioma	C0555198	MIR504	574507		microRNA 504		38	0.67	0.462		0.01		1	1	0	2015	2015
Malignant Glioma	C0555198	ADGRB1	575	O14514	adhesion G protein-coupled receptor B1	G-protein coupled receptor	34	0.695	0.231	1	0.01		1	1	0	2013	2013
Malignant Glioma	C0555198	SEMA6A	57556	Q9H2E6	semaphorin 6A	Signaling	211	0.486	0.808	0.9998	0.01		1	1	0	2016	2016
Malignant Glioma	C0555198	PTN	5764	P21246	pleiotrophin	Signaling	164	0.515	0.808	0.0075	0.01		1	1	0	2018	2018
Malignant Glioma	C0555198	ADGRB3	577	O60242	adhesion G protein-coupled receptor B3	G-protein coupled receptor	26	0.736	0.192	1	0.01		1	1	0	2004	2004
Malignant Glioma	C0555198	PTPN11	5781	Q06124	protein tyrosine phosphatase non-receptor type 11		702	0.385	0.923	1	0.01		1	1	0	2018	2018
Malignant Glioma	C0555198	BAX	581	Q07812	BCL2 associated X, apoptosis regulator	Signaling	420	0.417	0.885	0.3175	0.01		1	1	0	2007	2007
Malignant Glioma	C0555198	PXN	5829	P49023	paxillin	Cellular structure	116	0.547	0.654	0.0823	0.01		1	1	0	2017	2017
Malignant Glioma	C0555198	ENOPH1	58478	Q9UHY7	enolase-phosphatase 1	Enzyme	14	0.76	0.308	0.0061	0.01		1	1	0	2018	2018

Malignant Glioma	C0555198	RAB27B	5874	O00194	RAB27B, member RAS oncogene family		64	0.608	0.692	0.0387	0.01		1	1	0	2015	2015
Malignant Glioma	C0555198	PDIK1L	149420	Q8N165	PDLIM1 interacting kinase 1 like	Kinase	138	0.534	0.769	0.2644	0.01		1	1	0	2016	2016
Malignant Glioma	C0555198	VCAN	1462	P13611	versican	Extracellular structure	205	0.497	0.808	1	0.01		1	1	0	2007	2007
Malignant Glioma	C0555198	CCL5	6352	P13501	C-C motif chemokine ligand 5	Signaling	514	0.403	0.885	0.0018	0.01		1	1	0	2017	2017
Malignant Glioma	C0555198	CASD1	64921	Q96PB1	CAS1 domain containing 1		7	0.805	0.269	0.0448	0.01		1	1	0	1994	1994
Malignant Glioma	C0555198	SIX1	6495	Q15475	SIX homeobox 1	Transcription factor	200	0.496	0.692	0.653	0.01		1	1	0	2017	2017
Malignant Glioma	C0555198	SLC1A5	6510	Q15758	solute carrier family 1 member 5	Transporter	81	0.587	0.577	0.1687	0.01		1	1	0	2004	2004
Malignant Glioma	C0555198	BMP4	652	P12644	bone morphogenetic protein 4	Signaling	423	0.422	0.808	0.9564	0.01		1	1	0	2017	2017
Malignant Glioma	C0555198	PCSK6	5046	P29122	proprotein convertase subtilisin/kexin type 6	Enzyme	65	0.626	0.538	5E-13	0.01		1	1	0	2012	2012
Malignant Glioma	C0555198	CPOX	1371	P36551	coproporphyrinogen oxidase	Enzyme	246	0.488	0.846	0.1715	0.01		1	1	1	2017	2017
Malignant Glioma	C0555198	CXADRP1	653108		CXADR pseudogene 1		272	0.463	0.846		0.01		1	1	0	2007	2007
Malignant Glioma	C0555198	SLC34A1	6569	Q06495	solute carrier family 34 member 1	Transporter	73	0.619	0.423	9E-27	0.01		1	1	0	2014	2014
Malignant Glioma	C0555198	SNAI2	6591	O43623	snail family transcriptional repressor 2		189	0.505	0.692	0.7953	0.01		1	1	0	2019	2019
Malignant Glioma	C0555198	SNAI1	6615	O95863	snail family transcriptional repressor 1		243	0.474	0.808	0.2175	0.01		1	1	0	2019	2019
Malignant Glioma	C0555198	BNIP3	664	Q12983	BCL2 interacting protein 3		158	0.52	0.731	2E-05	0.01		1	1	0	2017	2017
Malignant Glioma	C0555198	SOAT1	6646	P35610	sterol O-acyltransferase 1	Enzyme	389	0.424	0.846	7E-10	0.01		1	1	0	2015	2015
Malignant Glioma	C0555198	CRH	1392	P06850	corticotropin releasing hormone	Signaling	402	0.439	0.808	0.7161	0.01		1	1	0	2012	2012
Malignant Glioma	C0555198	CRK	1398	P46108	CRK proto-oncogene, adaptor protein		544	0.394	0.923	0.9594	0.01		1	1	0	2013	2013
Malignant Glioma	C0555198	SIAH1	6477	Q8IUQ4	siah E3 ubiquitin protein ligase 1		54	0.631	0.538	0.6987	0.01		1	1	0	2015	2015
Malignant Glioma	C0555198	BIK	638	Q13323	BCL2 interacting killer		48	0.653	0.462	5E-06	0.01		1	1	0	2003	2003
Malignant Glioma	C0555198	SDC1	6382	P18827	syndecan 1	Extracellular structure	323	0.445	0.769	0.0084	0.01		1	1	0	2006	2006

Malignant Glioma	C0555198	GSC	145258	P56915	goosecoid homeobox		61	0.631	0.615	0.0139	0.01		1	1	0	2016	2016
Malignant Glioma	C0555198	NPAS3	64067	Q8IXF0	neuronal PAS domain protein 3	Transcription factor	29	0.729	0.269	0.9997	0.01		1	1	0	2011	2011
Malignant Glioma	C0555198	LAYN	143903	Q6UX15	layilin		22	0.722	0.385	1E-13	0.01		1	1	0	2019	2019
Malignant Glioma	C0555198	SAMSN1	64092	Q9NSI8	SAM domain, SH3 domain and nuclear localization signals 1		21	0.716	0.385	5E-11	0.01		1	1	0	2013	2013
Malignant Glioma	C0555198	MLST8	64223	Q9BVC4	MTOR associated protein, LST8 homolog		19	0.751	0.385	0.0082	0.01		1	1	0	2011	2011
Malignant Glioma	C0555198	SOX17	64321	Q9H6I2	SRY-box transcription factor 17	Transcription factor	113	0.555	0.615	0.885	0.01		1	1	0	2018	2018
Malignant Glioma	C0555198	CPEB1	64506	Q9BZB8	cytoplasmic polyadenylation element binding protein 1	Nucleic acid binding	57	0.638	0.538	0.999	0.01		1	1	0	2016	2016
Malignant Glioma	C0555198	SH3GL3	6457	Q99963	SH3 domain containing GRB2 like 3, endophilin A3		20	0.76	0.308	0.0001	0.01		1	1	0	2012	2012
Malignant Glioma	C0555198	SMYD3	64754	Q9H7B4	SET and MYND domain containing 3	Epigenetic regulator	57	0.633	0.462	1E-07	0.01		1	1	0	2015	2015
Malignant Glioma	C0555198	CREB3L2	64764	Q70SY1	cAMP responsive element binding protein 3 like 2		35	0.691	0.115	0.0015	0.01		1	1	0	2010	2010
Malignant Glioma	C0555198	CP	1356	P00450	ceruloplasmin	Enzyme	283	0.466	0.846	4E-10	0.01		1	1	0	2008	2008
Malignant Glioma	C0555198	NUMB	8650	P49757	NUMB endocytic adaptor protein	Signaling	55	0.619	0.731	0.004	0.01		1	1	0	2011	2011
Malignant Glioma	C0555198	CCND2	894	P30279	cyclin D2	Enzyme modulator	241	0.477	0.808	0.9866	0.01		1	1	0	1999	1999
Malignant Glioma	C0555198	CCND3	896	P30281	cyclin D3	Enzyme modulator	126	0.554	0.654	0.9835	0.01		1	1	0	1999	1999
Malignant Glioma	C0555198	NOL3	8996	O60936	nucleolar protein 3		100	0.575	0.808	0.0002	0.01		1	1	0	1985	1985
Malignant Glioma	C0555198	KLRC4-KLRK1	1E+08	P26718	KLRC4-KLRK1 readthrough		246	0.469	0.769		0.01		1	1	0	2013	2013
Malignant Glioma	C0555198	ANGPTL1	9068	O95841	angiopoietin like 1	Signaling	40	0.691	0.5	1E-10	0.01		1	1	0	2010	2010
Malignant Glioma	C0555198	CREB3L1	90993	Q96BA8	cAMP responsive element binding protein 3 like 1		88	0.601	0.615	0.9849	0.01		1	1	0	2018	2018
Malignant Glioma	C0555198	AIFM1	9131	O95831	apoptosis inducing factor mitochondria associated 1	Enzyme	205	0.527	0.769	0.998	0.01		1	1	0	2017	2017

Malignant Glioma	C0555198	CBFA2T2	9139	O43439	CBFA2/RUNX1 partner transcriptional co-repressor 2	Transcription factor	64	0.615	0.654	0.9959	0.01		1	1	0	1998	1998
Malignant Glioma	C0555198	MICA	1E+08	Q29983	MHC class I polypeptide-related sequence A		248	0.473	0.846	0.005	0.01		1	1	0	2003	2003
Malignant Glioma	C0555198	PDCD5	9141	O14737	programmed cell death 5	Enzyme modulator	67	0.61	0.538	0.0036	0.01		1	1	0	2012	2012
Malignant Glioma	C0555198	HOTAIRM1	1E+08		HOXA transcript antisense RNA, myeloid-specific 1		46	0.638	0.538		0.01		1	1	0	2019	2019
Malignant Glioma	C0555198	MIR1231	1E+08		microRNA 1231		20	0.736	0.308		0.01		1	1	0	2018	2018
Malignant Glioma	C0555198	MSC	9242	O60682	musculin		241	0.475	0.846	0.0247	0.01		1	1	0	2014	2014
Malignant Glioma	C0555198	MCM3AP	8888	O60318	minichromosome maintenance complex component 3 associated protein		58	0.647	0.654	5E-09	0.01		1	1	0	2009	2009
Malignant Glioma	C0555198	CDH11	1009	P55287	cadherin 11		203	0.512	0.808	0.9999	0.01		1	1	0	2012	2012
Malignant Glioma	C0555198	BECN1	8678	Q14457	beclin 1	Enzyme modulator	373	0.432	0.846	0.9372	0.01		1	1	0	2009	2009
Malignant Glioma	C0555198	PEA15	8682	Q15121	proliferation and apoptosis adaptor protein 15		110	0.553	0.692	0.7921	0.01		1	1	0	2016	2016
Malignant Glioma	C0555198	ABCC3	8714	O15438	ATP binding cassette subfamily C member 3	Transporter	160	0.517	0.769	1E-26	0.01		1	1	0	2001	2001
Malignant Glioma	C0555198	CDK6	1021	Q00534	cyclin dependent kinase 6	Kinase	266	0.471	0.731	0.9832	0.01		1	1	0	2016	2016
Malignant Glioma	C0555198	TNFRSF6B	8771	O95407	TNF receptor superfamily member 6b		145	0.528	0.731	4E-06	0.01		1	1	0	2001	2001
Malignant Glioma	C0555198	TRIM13	10206	O60858	tripartite motif containing 13		275	0.462	0.846	0.6366	0.01		1	1	0	2007	2007
Malignant Glioma	C0555198	TNFRSF10B	8795	O14763	TNF receptor superfamily member 10b		179	0.505	0.769	0.0002	0.01		1	1	0	2013	2013
Malignant Glioma	C0555198	TNFRSF10A	8797	O00220	TNF receptor superfamily member 10a		155	0.521	0.731	5E-15	0.01		1	1	0	1998	1998
Malignant Glioma	C0555198	TRIM24	8805	O15164	tripartite motif containing 24		79	0.581	0.615	1	0.01		1	1	0	2015	2015

Malignant Glioma	C0555198	TRIM28	10155	Q13263	tripartite motif containing 28		82	0.592	0.731	1	0.01		1	1	0	2018	2018
Malignant Glioma	C0555198	TRAP1	10131	Q12931	TNF receptor associated protein 1	Chaperone	90	0.59	0.615	2E-29	0.01		1	1	0	2015	2015
Malignant Glioma	C0555198	APLN	8862	Q9ULZ1	apelin		226	0.497	0.769	0.459	0.01		1	1	0	2007	2007
Malignant Glioma	C0555198	ARHGEF7	8874	Q14155	Rho guanine nucleotide exchange factor 7		215	0.486	0.846	1	0.01		1	1	0	1998	1998
Malignant Glioma	C0555198	DNER	92737	Q8NFT8	delta/notch like EGF repeat containing		203	0.493	0.769	0.1859	0.01		1	1	0	2017	2017
Malignant Glioma	C0555198	SLC38A5	92745	Q8WUX1	solute carrier family 38 member 5	Transporter	15	0.751	0.346	0.9768	0.01		1	1	0	2004	2004
Malignant Glioma	C0555198	TRAF4	9618	Q9BUZ4	TNF receptor associated factor 4	Signaling	61	0.626	0.615	0.936	0.01		1	1	0	2018	2018
Malignant Glioma	C0555198	IKBKE	9641	Q14164	inhibitor of nuclear factor kappa B kinase subunit epsilon	Kinase	79	0.597	0.731	0.009	0.01		1	1	0	2017	2017
Malignant Glioma	C0555198	CD63	967	P08962	CD63 molecule		128	0.542	0.731	0.0174	0.01		1	1	0	2010	2010
Malignant Glioma	C0555198	KDM4A	9682	O75164	lysine demethylase 4A		73	0.606	0.769	1	0.01		1	1	0	2016	2016
Malignant Glioma	C0555198	CD70	970	P32970	CD70 molecule		127	0.542	0.692	0.0104	0.01		1	1	0	2002	2002
Malignant Glioma	C0555198	SLK	9748	Q9H2G2	STE20 like kinase	Kinase	32	0.729	0.385	0.3744	0.01		1	1	0	2019	2019
Malignant Glioma	C0555198	ADGRE5	976	P48960	adhesion G protein-coupled receptor E5	G-protein coupled receptor	74	0.601	0.538	3E-06	0.01		1	1	0	2012	2012
Malignant Glioma	C0555198	KEAP1	9817	Q14145	kelch like ECH associated protein 1		251	0.475	0.808	0.0004	0.01		1	1	0	2011	2011
Malignant Glioma	C0555198	CDK1	983	P06493	cyclin dependent kinase 1	Kinase	214	0.482	0.808	0.9921	0.01		1	1	0	2015	2015
Malignant Glioma	C0555198	MELK	9833	Q14680	maternal embryonic leucine zipper kinase	Kinase	94	0.566	0.577	5E-22	0.01		1	1	0	2013	2013
Malignant Glioma	C0555198	FIG4	9896	Q92562	FIG4 phosphoinositide 5-phosphatase		223	0.551	0.731	2E-34	0.01		1	1	0	2009	2009
Malignant Glioma	C0555198	MRC2	9902	Q9UBG0	mannose receptor C type 2		35	0.705	0.385	0.6747	0.01		1	1	0	2011	2011
Malignant Glioma	C0555198	NR1I3	9970	Q14994	nuclear receptor subfamily 1 group I member 3	Nuclear receptor	318	0.448	0.846	4E-08	0.01		1	1	0	2007	2007
Malignant Glioma	C0555198	MIR543	1E+08		microRNA 543		68	0.593	0.692		0.01		1	1	0	2017	2017

Malignant Glioma	C0555198	WTAP	9589	Q15007	WT1 associated protein		37	0.659	0.577	0.9997	0.01		1	1	0	2016	2016
Malignant Glioma	C0555198	SLIT2	9353	O94813	slit guidance ligand 2		177	0.511	0.769	1	0.01		1	1	0	2009	2009
Malignant Glioma	C0555198	PPIG	9360	Q13427	peptidylprolyl isomerase G		203	0.497	0.846	0.9629	0.01		1	1	0	2001	2001
Malignant Glioma	C0555198	SLC9A3R1	9368	O14745	SLC9A3 regulator 1		116	0.547	0.577	9E-05	0.01		1	1	0	2009	2009
Malignant Glioma	C0555198	COX5A	9377	P20674	cytochrome c oxidase subunit 5A	Enzyme	171	0.515	0.846	0.4941	0.01		1	1	0	2017	2017
Malignant Glioma	C0555198	CD27	939	P26842	CD27 molecule		154	0.517	0.692	0.01	0.01		1	1	0	2002	2002
Malignant Glioma	C0555198	GRAP2	9402	O75791	GRB2 related adaptor protein 2		538	0.394	0.923	0.0002	0.01		1	1	0	2013	2013
Malignant Glioma	C0555198	MIR1287	1E+08		microRNA 1287		15	0.769	0.192		0.01		1	1	0	2016	2016
Malignant Glioma	C0555198	MIR1224	1E+08		microRNA 1224		36	0.666	0.385		0.01		1	1	0	2015	2015
Malignant Glioma	C0555198	CD34	947	P28906	CD34 molecule		674	0.368	0.808	5E-06	0.01		1	1	0	2019	2019
Malignant Glioma	C0555198	ATG5	9474	Q9H1Y0	autophagy related 5	Transporter	282	0.462	0.885	0.9776	0.01		1	1	0	2009	2009
Malignant Glioma	C0555198	BCL2L11	10018	O43521	BCL2 like 11		173	0.51	0.731	0.8886	0.01		1	1	0	2012	2012
Malignant Glioma	C0555198	GDF15	9518	Q99988	growth differentiation factor 15	Signaling	389	0.429	0.808	3E-07	0.01		1	1	0	2010	2010
Malignant Glioma	C0555198	CXCL14	9547	O95715	C-X-C motif chemokine ligand 14		125	0.554	0.731	0.0373	0.01		1	1	0	2018	2018
Malignant Glioma	C0555198	CDC42	998	P60953	cell division cycle 42	Enzyme modulator	327	0.458	0.846	0.7873	0.01		1	1	0	2017	2017
Malignant Glioma	C0555198	CCR2	729230	P41597	C-C motif chemokine receptor 2		436	0.418	0.846	0.0234	0.01		1	1	0	2012	2012
Malignant Glioma	C0555198	AHSA1	10598	O95433	activator of HSP90 ATPase activity 1		526	0.396	0.923	0.999	0.01		1	1	0	2013	2013
Malignant Glioma	C0555198	RABAC1	10567	Q9UI14	Rab acceptor 1	Transporter	6	0.931	0.077	0.0053	0.01		1	1	0	2010	2010
Malignant Glioma	C0555198	KTWS	791122		Klippel-Trenaunay-Weber syndrome		26	0.705	0.269		0.01		1	1	0	2007	2007
Malignant Glioma	C0555198	MUL1	79594	Q969V5	mitochondrial E3 ubiquitin protein ligase 1		241	0.473	0.808	2E-06	0.01		1	1	0	2016	2016
Malignant Glioma	C0555198	CALCA	796	P01258;P06881	calcitonin related polypeptide alpha	Signaling	467	0.416	0.923	0.0156	0.01		1	1	0	2019	2019
Malignant Glioma	C0555198	MCPH1	79648	Q8NEM0	microcephalin 1	Nucleic acid binding	141	0.54	0.846	8E-30	0.01		1	1	0	2008	2008

Malignant Glioma	C0555198	AIMP2	7965	Q13155	aminoacyl tRNA synthetase complex interacting multifunctional protein 2		555	0.393	0.923	0.008	0.01		1	1	0	2013	2013
Malignant Glioma	C0555198	LIN28A	79727	Q9H9Z2	lin-28 homolog A	Nucleic acid binding	161	0.523	0.731	0.0194	0.01		1	1	0	2014	2014
Malignant Glioma	C0555198	GPNMB	10457	Q14956	glycoprotein nmb	Signaling	103	0.566	0.692	3E-27	0.01		1	1	0	2006	2006
Malignant Glioma	C0555198	LRRN2	10446	O75325	leucine rich repeat neuronal 2	Receptor	15	0.769	0.192	0.7805	0.01		1	1	0	1998	1998
Malignant Glioma	C0555198	CPEB4	80315	Q17RY0	cytoplasmic polyadenylation element binding protein 4	Nucleic acid binding	70	0.621	0.462	0.9856	0.01		1	1	0	2016	2016
Malignant Glioma	C0555198	CD276	80381	Q5ZPR3	CD276 molecule	Enzyme modulator	169	0.505	0.731	2E-09	0.01		1	1	0	2013	2013
Malignant Glioma	C0555198	FOSL1	8061	P15407	FOS like 1, AP-1 transcription factor subunit	Transcription factor	150	0.519	0.846	0.1585	0.01		1	1	0	2005	2005
Malignant Glioma	C0555198	ZFX	7543	P17010	zinc finger protein X-linked	Transcription factor	66	0.599	0.538	0.9983	0.01		1	1	0	2011	2011
Malignant Glioma	C0555198	TXNIP	10628	Q9H3M7	thioredoxin interacting protein		208	0.49	0.731	0.1592	0.01		1	1	0	2017	2017
Malignant Glioma	C0555198	TYR	7299	P14679	tyrosinase	Enzyme	281	0.473	0.808	5E-32	0.01		1	1	0	2004	2004
Malignant Glioma	C0555198	UBE2N	7334	P61088	ubiquitin conjugating enzyme E2 N		80	0.593	0.654	0.8816	0.01		1	1	0	2008	2008
Malignant Glioma	C0555198	UCHL1	7345	P09936	ubiquitin C-terminal hydrolase L1	Enzyme	260	0.482	0.885	0.991	0.01		1	1	0	2017	2017
Malignant Glioma	C0555198	UCN	7349	P55089	urocortin	Signaling	134	0.539	0.769	0.4496	0.01		1	1	0	2012	2012
Malignant Glioma	C0555198	SLC38A1	81539	Q9H2H9	solute carrier family 38 member 1	Transporter	64	0.615	0.769	0.9307	0.01		1	1	0	2004	2004
Malignant Glioma	C0555198	VPS51	738	Q9UID3	VPS51 subunit of GARP complex		292	0.452	0.846	1E-08	0.01		1	1	0	2001	2001
Malignant Glioma	C0555198	MTCO2P12	1.1E+08		MT-CO2 pseudogene 12		703	0.368	0.962		0.01		1	1	0	2011	2011
Malignant Glioma	C0555198	VIM	7431	P08670	vimentin		644	0.372	0.846	0.7126	0.01		1	1	0	2006	2006
Malignant Glioma	C0555198	VTN	7448	P04004	vitronectin		178	0.509	0.769	2E-10	0.01		1	1	0	2016	2016
Malignant Glioma	C0555198	WEE1	7465	P30291	WEE1 G2 checkpoint kinase	Kinase	116	0.545	0.769	0.9997	0.01		1	1	0	2018	2018
Malignant Glioma	C0555198	RASL10A	10633	Q92737	RAS like family 10 member A		10	0.792	0.077	0.1842	0.01		1	1	0	2012	2012

Malignant Glioma	C0555198	XPO1	7514	O14980	exportin 1	Receptor	178	0.504	0.692	1	0.01		1	1	0	2020	2020
Malignant Glioma	C0555198	PDPN	10630	Q86YL7	podoplanin		256	0.47	0.769	0.0003	0.01		1	1	0	2015	2015
Malignant Glioma	C0555198	COL18A1	80781	P39060	collagen type XVIII alpha 1 chain		323	0.449	0.808	2E-10	0.01		1	1	0	2018	2018
Malignant Glioma	C0555198	YAP1	10413	P46937	Yes associated protein 1	Enzyme modulator	374	0.432	0.808	0.9992	0.01		1	1	0	2017	2017
Malignant Glioma	C0555198	ECRG4	84417	Q9H1Z8	ECRG4 augurin precursor		30	0.7	0.346	0.0004	0.01		1	1	0	2009	2009
Malignant Glioma	C0555198	CASR	846	P41180	calcium sensing receptor	G-protein coupled receptor	517	0.41	0.846	0.0467	0.01		1	1	0	2007	2007
Malignant Glioma	C0555198	TEAD2	8463	Q15562	TEA domain transcription factor 2	Transcription factor	11	0.792	0.308	2E-06	0.01		1	1	0	2017	2017
Malignant Glioma	C0555198	EFS	10278	O43281	embryonal Fyn-associated substrate		75	0.597	0.615	2E-08	0.01		1	1	0	2010	2010
Malignant Glioma	C0555198	BEX2	84707	Q9BXY8	brain expressed X-linked 2		26	0.705	0.231	0.1008	0.01		1	1	0	2006	2006
Malignant Glioma	C0555198	PCGF1	84759	Q9BSM1	polycomb group ring finger 1		9	0.821	0.038	0.9971	0.01		1	1	0	2017	2017
Malignant Glioma	C0555198	CDKN1B	1027	P46527	cyclin dependent kinase inhibitor 1B		454	0.409	0.769	0.6238	0.01		1	1	0	2002	2002
Malignant Glioma	C0555198	CDKN1A	1026	P38936	cyclin dependent kinase inhibitor 1A		490	0.403	0.769	0.0019	0.01		1	1	0	2000	2000
Malignant Glioma	C0555198	RHPN2	85415	Q8IUC4	rhophilin Rho GTPase binding protein 2	Enzyme modulator	21	0.711	0.192	1E-07	0.01		1	1	0	2013	2013
Malignant Glioma	C0555198	LGR5	8549	O75473	leucine rich repeat containing G protein-coupled receptor 5	G-protein coupled receptor	131	0.542	0.654	3E-08	0.01		1	1	0	2014	2014
Malignant Glioma	C0555198	MSLN	10232	Q13421	mesothelin	Extracellular structure	146	0.525	0.692	6E-25	0.01		1	1	0	2017	2017
Malignant Glioma	C0555198	KHSRP	8570	Q92945	KH-type splicing regulatory protein	Enzyme	148	0.526	0.846	1	0.01		1	1	0	2013	2013
Malignant Glioma	C0555198	CDK7	1022	P50613	cyclin dependent kinase 7	Kinase	89	0.566	0.731	4E-07	0.01		1	1	0	2017	2017
Malignant Glioma	C0555198	RECK	8434	O95980	reversion inducing cysteine rich protein with kazal motifs	Enzyme modulator	123	0.547	0.731	2E-10	0.01		1	1	0	2017	2017
Malignant Glioma	C0555198	BRMS1L	84312	Q5PSV4	BRMS1 like transcriptional repressor		38	0.67	0.346	0.0578	0.01		1	1	0	2018	2018
Malignant Glioma	C0555198	HMGA2	8091	P52926	high mobility group AT-hook 2	Nucleic acid binding	382	0.429	0.808	0.8702	0.01		1	1	0	2014	2014

Malignant Glioma	C0555198	CALR	811	P27797	calreticulin	Calcium-binding protein	487	0.413	0.923	0.8913	0.01		1	1	0	2006	2006
Malignant Glioma	C0555198	SLC7A5	8140	Q01650	solute carrier family 7 member 5	Transporter	173	0.513	0.808	0.2351	0.01		1	1	0	2013	2013
Malignant Glioma	C0555198	NDRG1	10397	Q92597	N-myc downstream regulated 1	Enzyme	225	0.488	0.731	0.0113	0.01		1	1	0	2016	2016
Malignant Glioma	C0555198	KAZALD1	81621	Q96I82	Kazal type serine peptidase inhibitor domain 1		13	0.769	0.115	1E-06	0.01		1	1	0	2013	2013
Malignant Glioma	C0555198	USP9X	8239	Q93008	ubiquitin specific peptidase 9 X-linked		255	0.502	0.846	1	0.01		1	1	0	2016	2016
Malignant Glioma	C0555198	CAST	831	P20810	calpastatin		141	0.541	0.846	4E-12	0.01		1	1	0	1999	1999
Malignant Glioma	C0555198	FZD7	8324	O75084	frizzled class receptor 7	G-protein coupled receptor	70	0.601	0.538	0.0884	0.01		1	1	0	2019	2019
Malignant Glioma	C0555198	CASP1	834	P29466	caspase 1	Enzyme	444	0.413	0.885	4E-05	0.01		1	1	0	1995	1995
Malignant Glioma	C0555198	TFG	10342	Q92734	trafficking from ER to golgi regulator		104	0.595	0.615	0.1406	0.01		1	1	0	2000	2000
Malignant Glioma	C0555198	SOX7	83595	Q9BT81	SRY-box transcription factor 7	Transcription factor	75	0.599	0.577	3E-07	0.01		1	1	0	2018	2018
Malignant Glioma	C0555198	TAGLN2	8407	P37802	transgelin 2	Cellular structure	64	0.604	0.577	0.0094	0.01		1	1	0	2017	2017
Malignant Glioma	C0555198	CDKN1C	1028	P49918	cyclin dependent kinase inhibitor 1C		298	0.456	0.846	0.8196	0.01		1	1	0	2004	2004
Malignant Glioma	C0555198	RDH16	8608	O75452	retinol dehydrogenase 16	Enzyme	4	0.931	0.077	4E-07	0.01		1	1	0	2017	2017
Malignant Glioma	C0555198	ARAF	369	P10398	A-Raf proto-oncogene, serine/threonine kinase	Kinase	56	0.619	0.654	0.9632	0.01		1	1	0	2009	2009
Malignant Glioma	C0555198	PART1	25859		prostate androgen-regulated transcript 1		250	0.483	0.885		0.01		1	1	0	2018	2018
Malignant Glioma	C0555198	PCSK9	255738	Q8NBP7	proprotein convertase subtilisin/kexin type 9	Enzyme	257	0.482	0.808	3E-17	0.01		1	1	0	2015	2015
Malignant Glioma	C0555198	LGALS9	3965	O00182	galectin 9	Signaling	197	0.496	0.769	7E-06	0.01		1	1	0	2020	2020
Malignant Glioma	C0555198	GABPA	2551	Q06546	GA binding protein transcription factor subunit alpha	Transcription factor	632	0.379	0.885	0.9981	0.01		1	1	0	2015	2015

Malignant Glioma	C0555198	LLGL1	3996	Q15334	LLGL scribble cell polarity complex component 1	Transporter	48	0.636	0.423	2E-05	0.01		1	1	0	2015	2015
Malignant Glioma	C0555198	LPL	4023	P06858	lipoprotein lipase	Enzyme	290	0.474	0.808	1E-09	0.01		1	1	0	2017	2017
Malignant Glioma	C0555198	LINC01194	404663		long intergenic non-protein coding RNA 1194		249	0.47	0.769		0.01		1	1	0	2011	2011
Malignant Glioma	C0555198	LTBP1	4052	Q14766	latent transforming growth factor beta binding protein 1	Calcium-binding protein	43	0.67	0.5	4E-09	0.01		1	1	0	2018	2018
Malignant Glioma	C0555198	MIR106A	406899		microRNA 106a		140	0.524	0.846		0.01		1	1	0	2011	2011
Malignant Glioma	C0555198	MIR107	406901		microRNA 107		123	0.543	0.769		0.01		1	1	0	2016	2016
Malignant Glioma	C0555198	MIR10B	406903		microRNA 10b		167	0.509	0.808		0.01		1	1	0	2009	2009
Malignant Glioma	C0555198	ACKR1	2532	Q16570	atypical chemokine receptor 1 (Duffy blood group)	G-protein coupled receptor	91	0.608	0.731	0.0002	0.01		1	1	0	2006	2006
Malignant Glioma	C0555198	LDHA	3939	P00338	lactate dehydrogenase A	Enzyme	185	0.507	0.808	0.0692	0.01		1	1	0	2016	2016
Malignant Glioma	C0555198	LASP1	3927	Q14847	LIM and SH3 protein 1		85	0.572	0.615	0.9827	0.01		1	1	0	2019	2019
Malignant Glioma	C0555198	RNF19A	25897	Q9NV58	ring finger protein 19A, RBR E3 ubiquitin protein ligase	Enzyme	523	0.397	0.923	0.0253	0.01		1	1	0	2013	2013
Malignant Glioma	C0555198	ITGB3	3690	P05106	integrin subunit beta 3	Receptor	260	0.485	0.846	0.0017	0.01		1	1	0	2016	2016
Malignant Glioma	C0555198	GSTK1	373156	Q9Y2Q3	glutathione S-transferase kappa 1		445	0.412	0.885	2E-06	0.01		1	1	0	2005	2005
Malignant Glioma	C0555198	ASPM	259266	Q8IZT6	abnormal spindle microtubule assembly		159	0.526	0.769	3E-49	0.01		1	1	0	2008	2008
Malignant Glioma	C0555198	KCNJ3	3760	P48549	potassium inwardly rectifying channel subfamily J member 3	Ion channel	46	0.653	0.5	0.9911	0.01		1	1	0	2008	2008
Malignant Glioma	C0555198	GALNT2	2590	Q10471	polypeptide N-acetylgalactosaminyl transferase 2	Enzyme	45	0.678	0.538	0.0084	0.01		1	1	0	2012	2012
Malignant Glioma	C0555198	KHK	3795	P50053	ketohexokinase	Kinase	28	0.7	0.346	2E-09	0.01		1	1	0	2018	2018

Malignant Glioma	C0555198	KIT	3815	P10721	KIT proto-oncogene, receptor tyrosine kinase	Kinase	715	0.366	0.808	0.9809	0.01		1	1	0	2007	2007
Malignant Glioma	C0555198	LINC00320	387486		long intergenic non-protein coding RNA 320		2	0.931	0.038		0.01		1	1	0	2019	2019
Malignant Glioma	C0555198	TRABD2B	388630	A6NFA1	TraB domain containing 2B		2	0.931	0.038	0.8427	0.01		1	1	0	2014	2014
Malignant Glioma	C0555198	L1CAM	3897	P32004	L1 cell adhesion molecule		289	0.475	0.769	1	0.01		1	1	0	2008	2008
Malignant Glioma	C0555198	RND3	390	P61587	Rho family GTPase 3	Enzyme modulator	46	0.656	0.385	0.9668	0.01		1	1	0	2015	2015
Malignant Glioma	C0555198	LAMP1	3916	P11279	lysosomal associated membrane protein 1	Transporter	120	0.542	0.769	0.8747	0.01		1	1	0	2018	2018
Malignant Glioma	C0555198	FUT4	2526	P22083	fucosyltransferase 4	Enzyme	168	0.505	0.731	8E-06	0.01		1	1	0	2019	2019
Malignant Glioma	C0555198	MIR17	406952		microRNA 17		270	0.459	0.846		0.01		1	1	0	2018	2018
Malignant Glioma	C0555198	MIR181A2	406954		microRNA 181a-2		85	0.57	0.654		0.01		1	1	0	2010	2010
Malignant Glioma	C0555198	MIR29C	407026		microRNA 29c		183	0.496	0.808		0.01		1	1	0	2013	2013
Malignant Glioma	C0555198	MIR320A	407037		microRNA 320a		145	0.526	0.808		0.01		1	1	0	2017	2017
Malignant Glioma	C0555198	MIR34A	407040		microRNA 34a		428	0.421	0.846		0.01		1	1	0	2012	2012
Malignant Glioma	C0555198	SMAD3	4088	P84022	SMAD family member 3	Transcription factor	470	0.415	0.923	0.798	0.01		1	1	0	2019	2019
Malignant Glioma	C0555198	STS	412	P08842	steroid sulfatase	Enzyme	382	0.431	0.846	0.8089	0.01		1	1	0	2004	2004
Malignant Glioma	C0555198	CBX7	23492	O95931	chromobox 7	Epigenetic regulator	73	0.595	0.615	0.6853	0.01		1	1	0	2017	2017
Malignant Glioma	C0555198	QPRT	23475	Q15274	quinolinate phosphoribosyltransferase		15	0.736	0.346	0.0047	0.01		1	1	0	2016	2016
Malignant Glioma	C0555198	MCM2	4171	P49736	minichromosome maintenance complex component 2	Enzyme	139	0.527	0.731	3E-05	0.01		1	1	0	2014	2014
Malignant Glioma	C0555198	CD46	4179	P15529	CD46 molecule		258	0.477	0.808	5E-07	0.01		1	1	0	2006	2006
Malignant Glioma	C0555198	FOLH1	2346	Q04609	folate hydrolase 1	Enzyme	311	0.453	0.885	6E-09	0.01		1	1	0	2018	2018
Malignant Glioma	C0555198	SIRT1	23411	Q96EB6	sirtuin 1	Epigenetic regulator	675	0.378	0.885	0.0873	0.01		1	1	0	2018	2018

Malignant Glioma	C0555198	PPP1R13B	23368	Q96KQ4	protein phosphatase 1 regulatory subunit 13B	Enzyme modulator	70	0.599	0.692	0.9981	0.01		1	1	0	1998	1998
Malignant Glioma	C0555198	FOS	2353	P01100	Fos proto-oncogene, AP-1 transcription factor subunit	Transcription factor	528	0.411	0.885	0.2618	0.01		1	1	0	2013	2013
Malignant Glioma	C0555198	MIR27A	407018		microRNA 27a		248	0.466	0.846		0.01		1	1	0	2015	2015
Malignant Glioma	C0555198	MIR222	407007		microRNA 222		210	0.486	0.808		0.01		1	1	0	2017	2017
Malignant Glioma	C0555198	CX3CR1	1524	P49238	C-X3-C motif chemokine receptor 1	G-protein coupled receptor	310	0.457	0.885	0.0614	0.01		1	1	0	2010	2010
Malignant Glioma	C0555198	MIR183	406959		microRNA 183		190	0.494	0.885		0.01		1	1	0	2013	2013
Malignant Glioma	C0555198	MIR184	406960		microRNA 184		119	0.542	0.769		0.01		1	1	0	2015	2015
Malignant Glioma	C0555198	MTOR	2475	P42345	mechanistic target of rapamycin kinase	Kinase	960	0.343	0.885	1	0.01		1	1	0	2007	2007
Malignant Glioma	C0555198	MIR203A	406986		microRNA 203a		237	0.471	0.808		0.01		1	1	0	2016	2016
Malignant Glioma	C0555198	MIR205	406988		microRNA 205		210	0.482	0.846		0.01		1	1	0	2013	2013
Malignant Glioma	C0555198	MIR206	406989		microRNA 206		226	0.477	0.846		0.01		1	1	0	2019	2019
Malignant Glioma	C0555198	IL17RA	23765	Q96F46	interleukin 17 receptor A		159	0.529	0.808	0.0004	0.01		1	1	0	2016	2016
Malignant Glioma	C0555198	SMUG1	23583	Q53HV7	single-strand-selective monofunctional uracil-DNA glycosylase 1		1034	0.322	0.923	0.0005	0.01		1	1	0	2018	2018
Malignant Glioma	C0555198	ARR3	407	P36575	arrestin 3	Enzyme modulator	279	0.46	0.846	0.104	0.01		1	1	0	2007	2007
Malignant Glioma	C0555198	TACSTD2	4070	P09758	tumor associated calcium signal transducer 2		130	0.537	0.731	0.0047	0.01		1	1	0	2013	2013
Malignant Glioma	C0555198	ATP6V0A2	23545	Q9Y487	ATPase H+ transporting V0 subunit a2	Transporter	158	0.57	0.692	3E-11	0.01		1	1	0	2006	2006
Malignant Glioma	C0555198	MEF2D	4209	Q14814	myocyte enhancer factor 2D	Transcription factor	72	0.608	0.5	0.9971	0.01		1	1	0	2016	2016
Malignant Glioma	C0555198	H2AX	3014	P16104	H2A.X variant histone	Nucleic acid binding	212	0.484	0.808	0.1765	0.01		1	1	0	2016	2016
Malignant Glioma	C0555198	HOXA13	3209	P31271	homeobox A13		126	0.552	0.654	0.9551	0.01		1	1	0	2015	2015

Malignant Glioma	C0555198	GRM1	2911	Q13255	glutamate metabotropic receptor 1	G-protein coupled receptor	172	0.528	0.808	0.1428	0.01		1	1	0	2018	2018
Malignant Glioma	C0555198	HRG	3273	P04196	histidine rich glycoprotein	Enzyme modulator	72	0.603	0.577	6E-14	0.01		1	1	0	2009	2009
Malignant Glioma	C0555198	HSPA4	3308	P34932	heat shock protein family A (Hsp70) member 4		550	0.394	0.923	0.9995	0.01		1	1	0	2019	2019
Malignant Glioma	C0555198	HSPA5	3309	P11021	heat shock protein family A (Hsp70) member 5		350	0.434	0.885	0.7734	0.01		1	1	0	2007	2007
Malignant Glioma	C0555198	PYCARD	29108	Q9ULZ3	PYD and CARD domain containing	Enzyme	294	0.457	0.808	0.0002	0.01		1	1	0	2004	2004
Malignant Glioma	C0555198	SETD2	29072	Q9BYW2	SET domain containing 2, histone lysine methyltransferase	Epigenetic regulator	447	0.424	0.846	1	0.01		1	1	0	2007	2007
Malignant Glioma	C0555198	HSP90AA1	3320	P07900	heat shock protein 90 alpha family class A member 1	Chaperone	455	0.411	0.923	0.8603	0.01		1	1	0	2017	2017
Malignant Glioma	C0555198	HTC2	3342		hypertrichosis 2 (generalized, congenital)		511	0.392	0.808		0.01		1	1	0	2007	2007
Malignant Glioma	C0555198	NOB1	28987	Q9ULX3	NIN1 (RPN12) binding protein 1 homolog		50	0.633	0.423	1E-09	0.01		1	1	0	2013	2013
Malignant Glioma	C0555198	GRIA2	2891	P42262	glutamate ionotropic receptor AMPA type subunit 2	Ion channel	110	0.573	0.692	0.9992	0.01		1	1	0	2001	2001
Malignant Glioma	C0555198	HOXA-AS2	285943		HOXA cluster antisense RNA 2		41	0.663	0.385		0.01		1	1	0	2018	2018
Malignant Glioma	C0555198	HOXA10	3206	P31260	homeobox A10		122	0.538	0.692	0.8845	0.01		1	1	0	2010	2010
Malignant Glioma	C0555198	HOXA9	3205	P31269	homeobox A9		147	0.521	0.692	4E-05	0.01		1	1	0	2010	2010
Malignant Glioma	C0555198	NR4A1	3164	P22736	nuclear receptor subfamily 4 group A member 1	Nuclear receptor	216	0.49	0.731	0.3707	0.01		1	1	0	2011	2011
Malignant Glioma	C0555198	ANXA1	301	P04083	annexin A1		336	0.442	0.885	1E-07	0.01		1	1	0	2017	2017
Malignant Glioma	C0555198	EEF2K	29904	O00418	eukaryotic elongation factor 2 kinase	Kinase	52	0.641	0.385	8E-20	0.01		1	1	0	2013	2013
Malignant Glioma	C0555198	H3-3B	3021	P84243	H3.3 histone B		77	0.599	0.385	0.8068	0.01		1	1	0	2019	2019

Malignant Glioma	C0555198	HCLS1	3059	P14317	hematopoietic cell-specific Lyn substrate 1	Cellular structure	132	0.539	0.846	1E-06	0.01		1	1	0	2013	2013
Malignant Glioma	C0555198	ADGRE2	30817	Q9UHX3	adhesion G protein-coupled receptor E2	G-protein coupled receptor	86	0.581	0.615	3E-28	0.01		1	1	0	2012	2012
Malignant Glioma	C0555198	GSTM1	2944	P09488	glutathione S-transferase mu 1		627	0.38	0.923	0.0021	0.01		1	1	0	2013	2013
Malignant Glioma	C0555198	HK1	3098	P19367	hexokinase 1	Kinase	161	0.542	0.731	0.9146	0.01		1	1	0	2011	2011
Malignant Glioma	C0555198	HK2	3099	P52789	hexokinase 2	Kinase	152	0.515	0.769	0.0046	0.01		1	1	0	2011	2011
Malignant Glioma	C0555198	GRM5	2915	P41594	glutamate metabotropic receptor 5	G-protein coupled receptor	189	0.525	0.846	0.9987	0.01		1	1	0	2019	2019
Malignant Glioma	C0555198	GRM4	2914	Q14833	glutamate metabotropic receptor 4	G-protein coupled receptor	54	0.636	0.5	0.9931	0.01		1	1	0	2018	2018
Malignant Glioma	C0555198	GRM3	2913	Q14832	glutamate metabotropic receptor 3	G-protein coupled receptor	79	0.606	0.538	0.7846	0.01		1	1	0	2018	2018
Malignant Glioma	C0555198	HMGA1	3159	P17096	high mobility group AT-hook 1	Nucleic acid binding	206	0.494	0.769	0.8321	0.01		1	1	0	2012	2012
Malignant Glioma	C0555198	GPR26	2849	Q8NDV2	G protein-coupled receptor 26	G-protein coupled receptor	12	0.792	0.077	0.521	0.01		1	1	0	2009	2009
Malignant Glioma	C0555198	EMC10	284361	Q5UCC4	ER membrane protein complex subunit 10		14	0.76	0.231	9E-11	0.01		1	1	0	2011	2011
Malignant Glioma	C0555198	ANGPT1	284	Q15389	angiopoietin 1	Signaling	340	0.446	0.808	0.9521	0.01		1	1	0	2006	2006
Malignant Glioma	C0555198	BBC3	27113	Q96PG8; Q9BXH1	BCL2 binding component 3		157	0.517	0.769	0.004	0.01		1	1	0	2005	2005
Malignant Glioma	C0555198	CXCL8	3576	P10145	C-X-C motif chemokine ligand 8	Signaling	1254	0.31	0.962	0.0003	0.01		1	1	0	1994	1994
Malignant Glioma	C0555198	IL10	3586	P22301	interleukin 10		1679	0.281	0.923	0.0059	0.01		1	1	0	1998	1998
Malignant Glioma	C0555198	SIGLEC7	27036	Q9Y286	sialic acid binding Ig like lectin 7	Receptor	137	0.535	0.846	2E-07	0.01		1	1	0	2013	2013
Malignant Glioma	C0555198	LATS2	26524	Q9NRM7	large tumor suppressor kinase 2	Kinase	106	0.554	0.654	0.9881	0.01		1	1	0	2019	2019
Malignant Glioma	C0555198	GNL3	26354	Q9BVP2	G protein nucleolar 3		87	0.579	0.577	2E-19	0.01		1	1	0	2010	2010
Malignant Glioma	C0555198	IL13RA2	3598	Q14627	interleukin 13 receptor subunit alpha 2	Signaling	103	0.556	0.654	4E-07	0.01		1	1	0	2007	2007

Malignant Glioma	C0555198	POLDIP2	26073	Q9Y2S7	DNA polymerase delta interacting protein 2		530	0.396	0.923	0.4141	0.01		1	1	0	2013	2013
Malignant Glioma	C0555198	ING1	3621	Q9UK53	inhibitor of growth family member 1	Nucleic acid binding	99	0.573	0.692	0.0097	0.01		1	1	0	2008	2008
Malignant Glioma	C0555198	ITGA6	3655	P23229	integrin subunit alpha 6		137	0.539	0.769	2E-07	0.01		1	1	0	2020	2020
Malignant Glioma	C0555198	AR	367	P10275	androgen receptor	Nuclear receptor	854	0.351	0.846	0.9884	0.01		1	1	0	1997	1997
Malignant Glioma	C0555198	ITGA3	3675	P26006	integrin subunit alpha 3		111	0.573	0.731	4E-07	0.01		1	1	0	2015	2015
Malignant Glioma	C0555198	DKK3	27122	Q9UBP4	dickkopf WNT signaling pathway inhibitor 3		186	0.5	0.731	4E-06	0.01		1	1	0	2015	2015
Malignant Glioma	C0555198	GLS2	27165	Q9UI32	glutaminase 2	Enzyme	85	0.597	0.538	1E-10	0.01		1	1	0	2008	2008
Malignant Glioma	C0555198	IL2RB	3560	P14784	interleukin 2 receptor subunit beta	Receptor	226	0.491	0.846	0.291	0.01		1	1	0	2013	2013
Malignant Glioma	C0555198	TSEN54	283989	Q7Z6J9	tRNA splicing endonuclease subunit 54		72	0.659	0.5	2E-07	0.01		1	1	0	2011	2011
Malignant Glioma	C0555198	NEAT1	283131		nuclear paraspeckle assembly transcript 1		179	0.502	0.769		0.01		1	1	0	2016	2016
Malignant Glioma	C0555198	GNAS	2778	O95467;P63092;P84996;Q5JWF2	GNAS complex locus	Enzyme modulator	536	0.42	0.885	0.6808	0.01		1	1	0	2008	2008
Malignant Glioma	C0555198	MACC1	346389	Q6ZN28	MET transcriptional regulator MACC1		102	0.561	0.538	6E-19	0.01		1	1	0	2013	2013
Malignant Glioma	C0555198	GLS	2744	O94925	glutaminase	Enzyme	217	0.489	0.808	0.9615	0.01		1	1	0	2008	2008
Malignant Glioma	C0555198	IGF1	3479	P05019	insulin like growth factor 1		1206	0.318	0.885	0.2716	0.01		1	1	0	2002	2002
Malignant Glioma	C0555198	RABGEF1	27342	Q9UJ41	RAB guanine nucleotide exchange factor 1	Enzyme modulator	122	0.55	0.731	0.0006	0.01		1	1	0	2013	2013
Malignant Glioma	C0555198	RBPJ	3516	Q06330	recombination signal binding protein for immunoglobulin kappa J region	Nucleic acid binding	161	0.518	0.808	0.9985	0.01		1	1	0	2017	2017
Malignant Glioma	C0555198	UBE2S	27338	Q16763	ubiquitin conjugating enzyme E2 S		42	0.653	0.5	0.6317	0.01		1	1	0	2017	2017
Malignant Glioma	C0555198	BHLHE22	27319	Q8NFJ8	basic helix-loop-helix family member e22	Enzyme	67	0.595	0.692	0.7999	0.01		1	1	0	2013	2013

Malignant Glioma	C0555198	IL1A	3552	P01583	interleukin 1 alpha		1002	0.333	0.962	0.0002	0.01		1	1	0	2014	2014
Malignant Glioma	C0555198	GLB1	2720	P16278	galactosidase beta 1	Enzyme	385	0.448	0.808	8E-10	0.01		1	1	0	1998	1998
Malignant Glioma	C0555198	ITGA7	3679	Q13683	integrin subunit alpha 7		111	0.588	0.654	4E-17	0.01		1	1	0	2017	2017
Malignant Glioma	C0555198	ERCC1	2067	P07992	ERCC excision repair 1, endonuclease non-catalytic subunit	Enzyme	345	0.445	0.846	2E-07	0.01		1	1	1	2014	2014
Malignant Glioma	C0555198	TET3	200424	O43151	tet methylcytosine dioxygenase 3		59	0.633	0.615	1	0.01		1	1	0	2020	2020
Malignant Glioma	C0555198	EIF4G1	1981	Q04637	eukaryotic translation initiation factor 4 gamma 1	Nucleic acid binding	139	0.553	0.808	1	0.01		1	1	0	2018	2018
Malignant Glioma	C0555198	PHEX	5251	P78562	phosphate regulating endopeptidase homolog X-linked	Enzyme	142	0.548	0.808	1	0.01		1	1	0	2005	2005
Malignant Glioma	C0555198	SERPINA1	5265	P01009	serpin family A member 1	Enzyme modulator	482	0.41	0.923	7E-08	0.01		1	1	0	2018	2018
Malignant Glioma	C0555198	EIF4EBP2	1979	Q13542	eukaryotic translation initiation factor 4E binding protein 2	Nucleic acid binding	25	0.716	0.423	0.3055	0.01		1	1	0	2018	2018
Malignant Glioma	C0555198	EIF4E	1977	P06730	eukaryotic translation initiation factor 4E	Nucleic acid binding	317	0.448	0.846	0.9413	0.01		1	1	0	2018	2018
Malignant Glioma	C0555198	EIF4A2	1974	Q14240	eukaryotic translation initiation factor 4A2		74	0.604	0.731	0.9984	0.01		1	1	0	2018	2018
Malignant Glioma	C0555198	EEF1B2P2	1934		eukaryotic translation elongation factor 1 beta 2 pseudogene 2		58	0.612	0.577		0.01		1	1	0	2013	2013
Malignant Glioma	C0555198	PIK3R1	5295	P27986	phosphoinositide-3-kinase regulatory subunit 1	Kinase	285	0.477	0.885	0.9998	0.01		1	1	0	1998	1998
Malignant Glioma	C0555198	PIK3R2	5296	O00459	phosphoinositide-3-kinase regulatory subunit 2	Enzyme modulator	120	0.551	0.808	0.0163	0.01		1	1	0	1998	1998
Malignant Glioma	C0555198	PLAU	5328	P00749	plasminogen activator, urokinase	Enzyme	439	0.425	0.923	2E-06	0.01		1	1	0	2000	2000
Malignant Glioma	C0555198	ECT2	1894	Q9H8V3	epithelial cell transforming 2		65	0.603	0.615	8E-10	0.01		1	1	0	2014	2014
Malignant Glioma	C0555198	EMP1	2012	P54849	epithelial membrane protein 1	Cellular structure	114	0.56	0.769	0.4971	0.01		1	1	0	2019	2019

Malignant Glioma	C0555198	EMP3	2014	P54852	epithelial membrane protein 3	Cellular structure	55	0.619	0.423	3E-06	0.01		1	1	0	2018	2018
Malignant Glioma	C0555198	PECAM1	5175	P16284	platelet and endothelial cell adhesion molecule 1	Cell adhesion	396	0.426	0.846		0.01		1	1	0	2012	2012
Malignant Glioma	C0555198	EPS8	2059	Q12929	epidermal growth factor receptor pathway substrate 8	Receptor	73	0.608	0.615	0.9731	0.01		1	1	0	2013	2013
Malignant Glioma	C0555198	EPO	2056	P01588	erythropoietin		646	0.381	0.885	0.0113	0.01		1	1	0	2019	2019
Malignant Glioma	C0555198	ANGPTL4	51129	Q9BY76	angiopoietin like 4	Signaling	198	0.498	0.731	2E-11	0.01		1	1	0	2013	2013
Malignant Glioma	C0555198	EPHB4	2050	P54760	EPH receptor B4	Kinase	157	0.524	0.692	0.0135	0.01		1	1	0	2001	2001
Malignant Glioma	C0555198	EPHB1	2047	P54762	EPH receptor B1	Kinase	169	0.518	0.846	0.9985	0.01		1	1	0	2013	2013
Malignant Glioma	C0555198	TNFRSF12A	51330	Q9NP84	TNF receptor superfamily member 12A		173	0.509	0.692	0.018	0.01		1	1	0	2006	2006
Malignant Glioma	C0555198	ZMYND10	51364	O75800	zinc finger MYND-type containing 10		126	0.553	0.731	2E-07	0.01		1	1	0	2008	2008
Malignant Glioma	C0555198	PPME1	51400	Q9Y570	protein phosphatase methylesterase 1	Enzyme	18	0.736	0.308	0.999	0.01		1	1	0	2009	2009
Malignant Glioma	C0555198	PDE4C	5143	Q08493	phosphodiesterase 4C		10	0.805	0.154	1E-07	0.01		1	1	0	2014	2014
Malignant Glioma	C0555198	PDGFB	5155	P01127	platelet derived growth factor subunit B	Signaling	301	0.488	0.846	0.7701	0.01		1	1	0	2010	2010
Malignant Glioma	C0555198	PDGFRB	5159	P09619	platelet derived growth factor receptor beta	Kinase	492	0.416	0.846	0.9041	0.01		1	1	0	2013	2013
Malignant Glioma	C0555198	WWOX	51741	Q9NZC7	WW domain containing oxidoreductase	Enzyme	356	0.453	0.808	1E-15	0.01		1	1	0	2015	2015
Malignant Glioma	C0555198	APLNR	187	P35414	apelin receptor	G-protein coupled receptor	94	0.588	0.692	0.7891	0.01		1	1	0	2007	2007
Malignant Glioma	C0555198	PLTP	5360	P55058	phospholipid transfer protein		62	0.633	0.654	9E-09	0.01		1	1	0	2017	2017
Malignant Glioma	C0555198	MBD3	53615	O95983	methyl-CpG binding domain protein 3		31	0.682	0.462	0.9765	0.01		1	1	0	2016	2016
Malignant Glioma	C0555198	PRKAR1A	5573	P10644	protein kinase cAMP-dependent type I regulatory subunit alpha		597	0.401	0.846	0.9999	0.01		1	1	0	2007	2007

Malignant Glioma	C0555198	BEX1	55859	Q9HBH7	brain expressed X-linked 1		21	0.729	0.115	0.5427	0.01		1	1	0	2006	2006
Malignant Glioma	C0555198	MAPK3	5595	P27361	mitogen-activated protein kinase 3	Kinase	647	0.379	0.885	0.0369	0.01		1	1	0	2016	2016
Malignant Glioma	C0555198	DES	1674	P17661	desmin		330	0.46	0.846	0.0088	0.01		1	1	0	2009	2009
Malignant Glioma	C0555198	MAPK8	5599	P45983	mitogen-activated protein kinase 8	Kinase	520	0.397	0.885	0.9982	0.01		1	1	0	2006	2006
Malignant Glioma	C0555198	METTL3	56339	Q86U44	methyltransferase like 3		79	0.585	0.538	2E-07	0.01		1	1	0	2019	2019
Malignant Glioma	C0555198	KLK6	5653	Q92876	kallikrein related peptidase 6	Enzyme	99	0.575	0.654	0.006	0.01		1	1	0	2009	2009
Malignant Glioma	C0555198	DIABLO	56616	Q9NR28	diablo IAP-binding mitochondrial protein		108	0.555	0.692	3E-06	0.01		1	1	0	2002	2002
Malignant Glioma	C0555198	LHX9	56956	Q9NQ69	LIM homeobox 9	Nucleic acid binding	9	0.805	0.231	0.9806	0.01		1	1	0	2009	2009
Malignant Glioma	C0555198	SLC12A9	56996	Q9BXP2	solute carrier family 12 member 9	Transporter	280	0.454	0.769	1E-15	0.01		1	1	0	2000	2000
Malignant Glioma	C0555198	GOPC	57120	Q9HD26	golgi associated PDZ and coiled-coil motif containing		103	0.561	0.654	0.0008	0.01		1	1	0	2017	2017
Malignant Glioma	C0555198	DCT	1638	P40126	dopachrome tautomerase	Enzyme	59	0.619	0.654	7E-14	0.01		1	1	0	2004	2004
Malignant Glioma	C0555198	DEPDC1	55635	Q5TB30	DEP domain containing 1	Nucleic acid binding	38	0.67	0.577	8E-10	0.01		1	1	0	2017	2017
Malignant Glioma	C0555198	MIR429	554210		microRNA 429		117	0.544	0.731		0.01		1	1	0	2018	2018
Malignant Glioma	C0555198	DOCK1	1793	Q14185	dedicator of cytokinesis 1		58	0.641	0.577	0.0068	0.01		1	1	0	2007	2007
Malignant Glioma	C0555198	TLR9	54106	Q9NR96	toll like receptor 9		457	0.411	0.885	1E-05	0.01		1	1	0	2016	2016
Malignant Glioma	C0555198	POLD1	5424	P28340	DNA polymerase delta 1, catalytic subunit		189	0.507	0.769	2E-06	0.01		1	1	0	2015	2015
Malignant Glioma	C0555198	TERF2IP	54386	Q9NYB0	TERF2 interacting protein		126	0.548	0.731	3E-05	0.01		1	1	0	2013	2013
Malignant Glioma	C0555198	NLGN3	54413	Q9NZ94	neuroligin 3		63	0.653	0.462	0.9764	0.01		1	1	0	2017	2017
Malignant Glioma	C0555198	DUSP5	1847	Q16690	dual specificity phosphatase 5		78	0.597	0.654	0.0033	0.01		1	1	0	2019	2019
Malignant Glioma	C0555198	PPP1R12C	54776	Q9BZL4	protein phosphatase 1 regulatory subunit 12C		53	0.626	0.654	0.0867	0.01		1	1	0	1998	1998
Malignant Glioma	C0555198	PPIB	5479	P23284	peptidylprolyl isomerase B		59	0.641	0.577	5E-05	0.01		1	1	0	2014	2014

Malignant Glioma	C0555198	HBEGF	1839	Q99075	heparin binding EGF like growth factor	Signaling	155	0.52	0.769	0.0756	0.01		1	1	0	1998	1998
Malignant Glioma	C0555198	ANKRD49	54851	Q8WVL7	ankyrin repeat domain 49	Enzyme modulator	7	0.861	0.077	0.0005	0.01		1	1	0	2017	2017
Malignant Glioma	C0555198	CMTM6	54918	Q9NX76	CKLF like MARVEL transmembrane domain containing 6		18	0.78	0.192	0.433	0.01		1	1	0	2018	2018
Malignant Glioma	C0555198	PPP1R1A	5502	Q13522	protein phosphatase 1 regulatory inhibitor subunit 1A	Enzyme modulator	106	0.559	0.692	0.0219	0.01		1	1	0	1999	1999
Malignant Glioma	C0555198	PID1	55022	Q7Z2X4	phosphotyrosine interaction domain containing 1		29	0.7	0.231	0.0031	0.01		1	1	0	2014	2014
Malignant Glioma	C0555198	DCTD	1635	P32321	dCMP deaminase	Enzyme	10	0.78	0.192	0.0007	0.01		1	1	0	2017	2017
Malignant Glioma	C0555198	FN1	2335	P02751	fibronectin 1	Signaling	724	0.365	0.962	0.0014	0.01		1	1	0	2010	2010
Malignant Glioma	C0555198	MIR335	442904		microRNA 335		141	0.521	0.808		0.01		1	1	0	2012	2012
Malignant Glioma	C0555198	MIR338	442906		microRNA 338		128	0.535	0.808		0.01		1	1	0	2018	2018
Malignant Glioma	C0555198	MIR346	442911		microRNA 346		52	0.631	0.615		0.01		1	1	0	2016	2016
Malignant Glioma	C0555198	MIR367	442912		microRNA 367		71	0.59	0.654		0.01		1	1	0	2015	2015
Malignant Glioma	C0555198	KLRK1	22914	P26718	killer cell lectin like receptor K1		255	0.467	0.769	7E-14	0.01		1	1	0	2013	2013
Malignant Glioma	C0555198	MSH3	4437	P20585	mutS homolog 3	Nucleic acid binding	245	0.474	0.769	7E-31	0.01		1	1	0	2001	2001
Malignant Glioma	C0555198	FGFR4	2264	P22455	fibroblast growth factor receptor 4	Kinase	220	0.49	0.769	7E-13	0.01		0	1	0	2006	2006
Malignant Glioma	C0555198	COX2	4513	P00403	cytochrome c oxidase subunit II	Enzyme	875	0.352	0.962		0.01		1	1	0	2011	2011
Malignant Glioma	C0555198	MTCP1	4515	P56278	mature T cell proliferation 1		9	0.821	0.308	0.4521	0.01		1	1	0	2018	2018
Malignant Glioma	C0555198	MTHFR	4524	P42898	methylenetetrahydrofolate reductase		985	0.337	0.885	3E-10	0.01		0	1	0	2006	2006
Malignant Glioma	C0555198	MVD	4597	P53602	mevalonate diphosphate decarboxylase		101	0.568	0.769	3E-17	0.01		1	1	0	2012	2012
Malignant Glioma	C0555198	MXI1	4601	P50539	MAX interactor 1, dimerization protein	Transcription factor	128	0.547	0.615	0.0011	0.01		1	1	0	1995	1995
Malignant Glioma	C0555198	MIR133B	442890		microRNA 133b		109	0.555	0.808		0.01		1	1	0	2015	2015

Malignant Glioma	C0555198	CD93	22918	Q9NPY3	CD93 molecule	Calcium-binding protein	36	0.663	0.5	2E-10	0.01		1	1	0	2018	2018
Malignant Glioma	C0555198	ZFAS1	441951		ZNFX1 antisense RNA 1		82	0.582	0.615		0.01		1	1	0	2020	2020
Malignant Glioma	C0555198	FLT1	2321	P17948	fms related receptor tyrosine kinase 1	Kinase	424	0.419	0.846	0.9998	0.01		1	1	0	2017	2017
Malignant Glioma	C0555198	KDM6B	23135	O15054	lysine demethylase 6B	Epigenetic regulator	173	0.514	0.808	1	0.01		1	1	0	2015	2015
Malignant Glioma	C0555198	FOXO3	2309	O43524	forkhead box O3		381	0.431	0.808	0.9881	0.01		1	1	0	2016	2016
Malignant Glioma	C0555198	MMP3	4314	P08254	matrix metallopeptidase 3	Enzyme	473	0.408	0.885	6E-15	0.01		1	1	0	2010	2010
Malignant Glioma	C0555198	PEG10	23089	Q86TG7	paternally expressed 10		75	0.597	0.615	0.8727	0.01		1	1	0	2019	2019
Malignant Glioma	C0555198	MMP11	4320	P24347	matrix metallopeptidase 11	Enzyme	130	0.542	0.692	2E-05	0.01		1	1	0	2008	2008
Malignant Glioma	C0555198	MMP19	4327	Q99542	matrix metallopeptidase 19	Enzyme	64	0.623	0.615	2E-20	0.01		1	1	0	2008	2008
Malignant Glioma	C0555198	CD200	4345	P41217	CD200 molecule		149	0.529	0.769	0.4136	0.01		1	1	0	2019	2019
Malignant Glioma	C0555198	MPI	4351	P34949	mannose phosphate isomerase	Enzyme	101	0.585	0.654	1E-07	0.01		1	1	0	2014	2014
Malignant Glioma	C0555198	MRC1	4360	P22897	mannose receptor C-type 1		431	0.413	0.846	0.9434	0.01		1	1	0	2013	2013
Malignant Glioma	C0555198	ABCC1	4363	P33527	ATP binding cassette subfamily C member 1	Transporter	333	0.445	0.808	0.0012	0.01		1	1	0	2001	2001
Malignant Glioma	C0555198	EIF2AK4	440275	Q9P2K8	eukaryotic translation initiation factor 2 alpha kinase 4		89	0.603	0.577	1E-15	0.01		1	1	0	2020	2020
Malignant Glioma	C0555198	HOXA11-AS	221883		HOXA11 antisense RNA		60	0.61	0.423		0.01		1	1	0	2019	2019
Malignant Glioma	C0555198	FAT1	2195	Q14517	FAT atypical cadherin 1		178	0.508	0.808	1E-12	0.01		1	1	0	2016	2016
Malignant Glioma	C0555198	MYCN	4613	P04198	MYCN proto-oncogene, bHLH transcription factor	Transcription factor	314	0.454	0.808	0.889	0.01		1	1	0	1994	1994
Malignant Glioma	C0555198	EREG	2069	O14944	epiregulin	Signaling	121	0.554	0.692	1E-05	0.01		1	1	0	2013	2013
Malignant Glioma	C0555198	NTS	4922	P30990	neurotensin		184	0.517	0.769	4E-05	0.01		1	1	0	2015	2015
Malignant Glioma	C0555198	NTSR1	4923	P30989	neurotensin receptor 1	G-protein coupled receptor	124	0.563	0.692	2E-08	0.01		1	1	0	2015	2015

Malignant Glioma	C0555198	OAP	4937		osteoarthrosis, precocious		9	0.792	0.192		0.01		1	1	0	2019	2019
Malignant Glioma	C0555198	MIR361	494323		microRNA 361		79	0.578	0.654		0.01		1	1	0	2018	2018
Malignant Glioma	C0555198	SLC22A18	5002	Q96BI1	solute carrier family 22 member 18	Transporter	76	0.595	0.692	6E-06	0.01		0	1	0	2018	2018
Malignant Glioma	C0555198	P4HB	5034	P07237	prolyl 4-hydroxylase subunit beta		222	0.488	0.808	0.9966	0.01		1	1	0	2013	2013
Malignant Glioma	C0555198	PEBP1	5037	P30086	phosphatidylethanolamine binding protein 1	Enzyme modulator	181	0.5	0.808	0.0917	0.01		1	1	0	2011	2011
Malignant Glioma	C0555198	PAEP	5047	P09466	progestagen associated endometrial protein	Enzyme	397	0.43	0.846	3E-08	0.01		1	1	0	2019	2019
Malignant Glioma	C0555198	PRKN	5071	O60260	parkin RBR E3 ubiquitin protein ligase	Enzyme	409	0.431	0.846	7E-07	0.01		1	1	0	2016	2016
Malignant Glioma	C0555198	PAX6	5080	P26367	paired box 6		340	0.45	0.769	0.9998	0.01		1	1	0	2012	2012
Malignant Glioma	C0555198	PCBP2	5094	Q15366	poly(rC) binding protein 2	Enzyme	44	0.647	0.577	0.9962	0.01		1	1	0	2016	2016
Malignant Glioma	C0555198	ESR2	2100	Q92731	estrogen receptor 2	Nuclear receptor	528	0.4	0.923	4E-08	0.01		1	1	0	2017	2017
Malignant Glioma	C0555198	ALB	213	P02768	albumin	Transporter	1198	0.317	0.962	0.6433	0.01		1	1	0	2017	2017
Malignant Glioma	C0555198	NPTX2	4885	P47972	neuronal pentraxin 2		67	0.617	0.615	0.0305	0.01		1	1	0	2007	2007
Malignant Glioma	C0555198	PTK2B	2185	Q14289	protein tyrosine kinase 2 beta	Kinase	319	0.448	0.808	0.9354	0.01		1	1	0	2005	2005
Malignant Glioma	C0555198	FABP3	2170	P05413	fatty acid binding protein 3		95	0.585	0.692	0.4433	0.01		1	1	0	2014	2014
Malignant Glioma	C0555198	NEK2	4751	P51955	NIMA related kinase 2	Kinase	103	0.572	0.692	1E-06	0.01		1	1	0	2017	2017
Malignant Glioma	C0555198	ATP1A1	476	P05023	ATPase Na+/K+ transporting subunit alpha 1	Transporter	89	0.601	0.808	1	0.01		1	1	0	2019	2019
Malignant Glioma	C0555198	F3	2152	P13726	coagulation factor III, tissue factor	Receptor	456	0.415	0.846	0.0189	0.01		1	1	0	2010	2010
Malignant Glioma	C0555198	NF2	4771	P35240	neurofibromin 2		312	0.468	0.885	1	0.01		1	1	0	1998	1998
Malignant Glioma	C0555198	NFATC2	4773	Q13469	nuclear factor of activated T cells 2	Transcription factor	154	0.525	0.731	1	0.01		1	1	0	2017	2017
Malignant Glioma	C0555198	F2RL1	2150	P55085	F2R like trypsin receptor 1	G-protein coupled receptor	256	0.48	0.769	0.0001	0.01		1	1	0	2010	2010

Malignant Glioma	C0555198	NFKBIA	4792	P25963	NFKB inhibitor alpha		226	0.487	0.885	0.9945	0.01		1	1	0	2016	2016
Malignant Glioma	C0555198	NGFR	4804	P08138	nerve growth factor receptor	Receptor	256	0.477	0.846	0.0979	0.01		1	1	0	2020	2020
Malignant Glioma	C0555198	NOTCH2	4853	Q04721	notch receptor 2		384	0.441	0.808	1	0.01		1	1	0	2009	2009
Malignant Glioma	C0555198	NOTCH3	4854	Q9UM47	notch receptor 3		418	0.435	0.808	0.4076	0.01		1	1	0	2013	2013
Malignant Glioma	C0555198	PCDH9	5101	Q9HC56	protocadherin 9		35	0.682	0.5	0.8087	0.01		1	1	0	2017	2017

4. Supplementary Table S4:Venn intersecting Common Targets

Target	Common name	Uniprot ID
Matrix metalloproteinase 1	MMP1	P03956
Calcium sensing receptor	CASR	P41180
Matrix metalloproteinase 9	MMP9	P14780
TNF-alpha	TNF	P01375
Caspase-1	CASP1	P29466
Caspase-6	CASP6	P55212
Matrix metalloproteinase 2	MMP2	P08253
Estrogen receptor beta	ESR2	Q92731
PI3-kinase p110-beta subunit	PIK3CB	P42338
Caspase-8	CASP8	Q14790
MAP kinase ERK1 (by homology)	MAPK3	P27361

5. Supplementary Table S5.Top 10 GENES in network ranked by Degree method

Rank	Name	Score
1	MAPK3	9
2	TNF	8
3	CASP8	7
3	MMP9	7
5	ESR2	6
5	CASP1	6
5	MMP2	6
8	MMP1	4
9	CASP6	3
10	PIK3CB	1

6. Supplementary Table S6: Enriched Pathways

Enrichment FDR	nGenes	Pathway Genes	Fold Enrichment	Pathway	Genes
1.02E-08	5	214	106.9205607	Path:hsa05417 Lipid and atherosclerosis	PIK3CB MAPK3 CASP1 CASP6 CASP8
2.95E-07	4	136	134.5941176	Path:hsa04210 Apoptosis	PIK3CB MAPK3 CASP6 CASP8
1.64E-05	3	103	133.2873786	Path:hsa04620 Toll-like receptor signaling pathway	PIK3CB MAPK3 CASP8
1.88E-05	3	112	122.5767857	Path:hsa04668 TNF signaling pathway	PIK3CB MAPK3 CASP8
0.000272621	3	384	35.7515625	Path:hsa05010 Alzheimer disease	PIK3CB MAPK3 CASP8
0.000346108	2	59	155.1254237	Path:hsa04370 VEGF signaling pathway	PIK3CB MAPK3
0.000402957	2	75	122.032	Path:hsa05214 Glioma	PIK3CB MAPK3
0.000423454	2	79	115.8531646	Path:hsa01521 EGFR tyrosine kinase inhibitor resistance	PIK3CB MAPK3
0.000514008	2	93	98.41290323	Path:hsa04657 IL-17 signaling pathway	MAPK3 CASP8
0.000603498	2	109	83.96697248	Path:hsa04066 HIF-1 signaling pathway	PIK3CB MAPK3
0.000881788	2	155	59.04774194	Path:hsa04150 mTOR signaling pathway	PIK3CB MAPK3
0.003629896	2	354	25.85423729	Path:hsa04151 PI3K-Akt signaling pathway	PIK3CB MAPK3
0.006429769	2	476	19.22773109	Path:hsa05022 Pathways of neurodegeneration-multiple diseases	MAPK3 CASP8
0.018687848	1	60	76.27	Path:hsa04730 Long-term depression	MAPK3
0.021278829	1	73	62.68767123	Path:hsa04115 p53 signaling pathway	CASP8
0.025933729	1	98	46.69591837	Path:hsa04750 Inflammatory mediator regulation of TRP channels	PIK3CB
0.036626525	1	162	28.24814815	Path:hsa04630 JAK-STAT signaling pathway	PIK3CB
0.063907594	1	294	15.56530612	Path:hsa04010 MAPK signaling pathway	MAPK3
0.000423454	3	530	25.90301887	Path:hsa05200 Pathways in cancer	PIK3CB MAPK3 CASP8

Supplementary figures

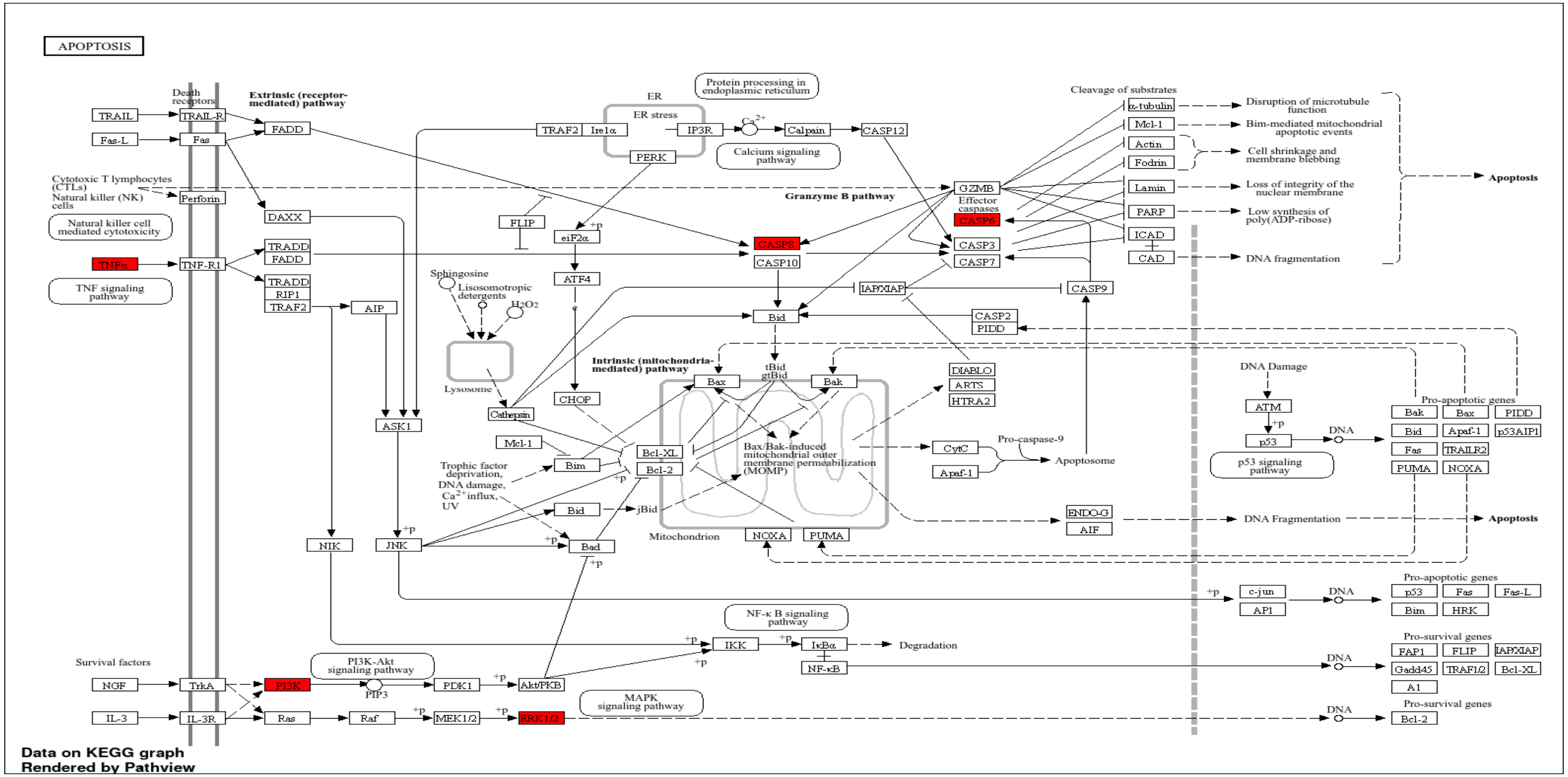

Supplementary Figure S1: Apoptosis

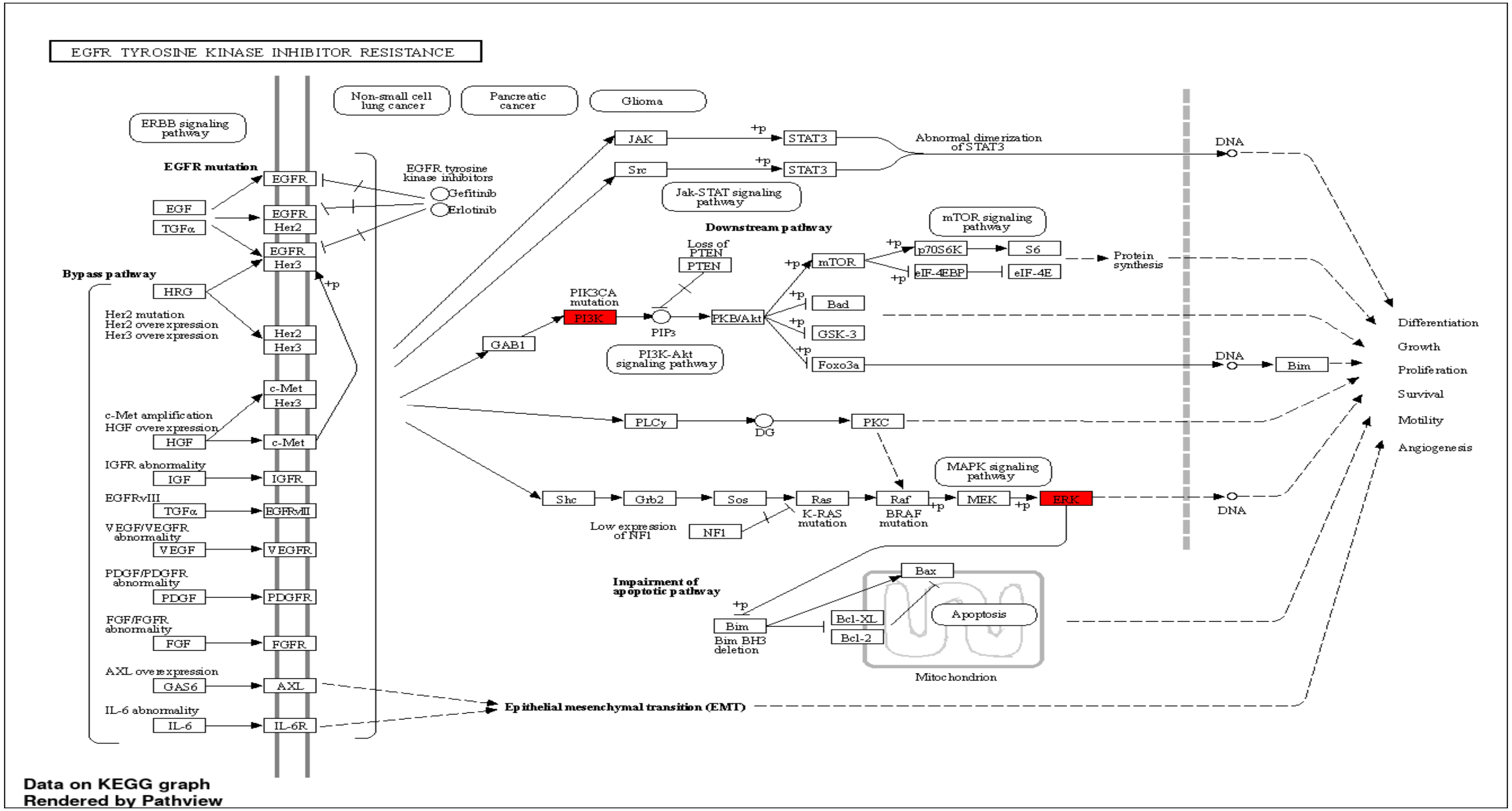

Supplementary Figure S2: EGFR Tyrosine Kinase inhibitor resistance pathway

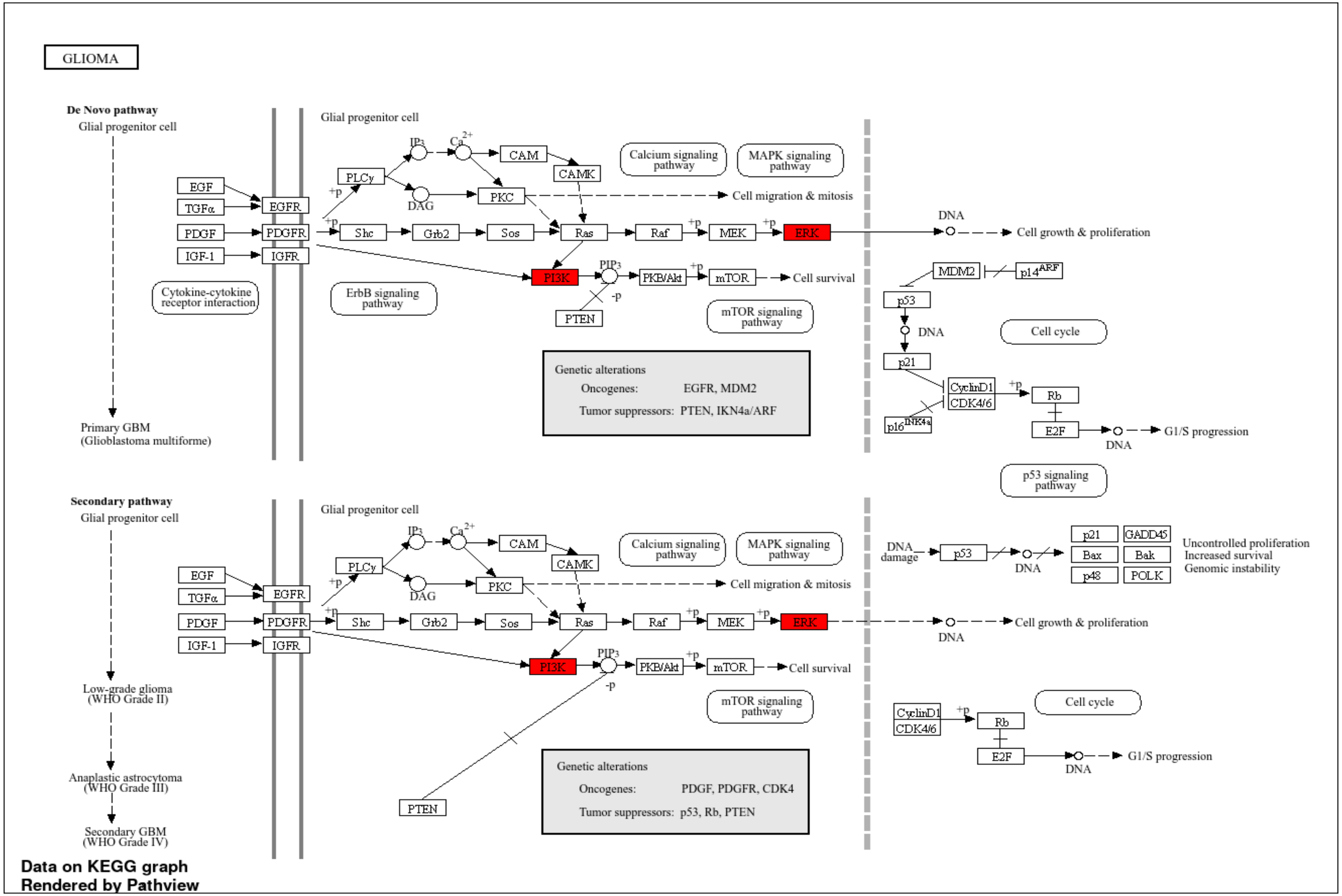

Supplementary Figure S3: Glioma

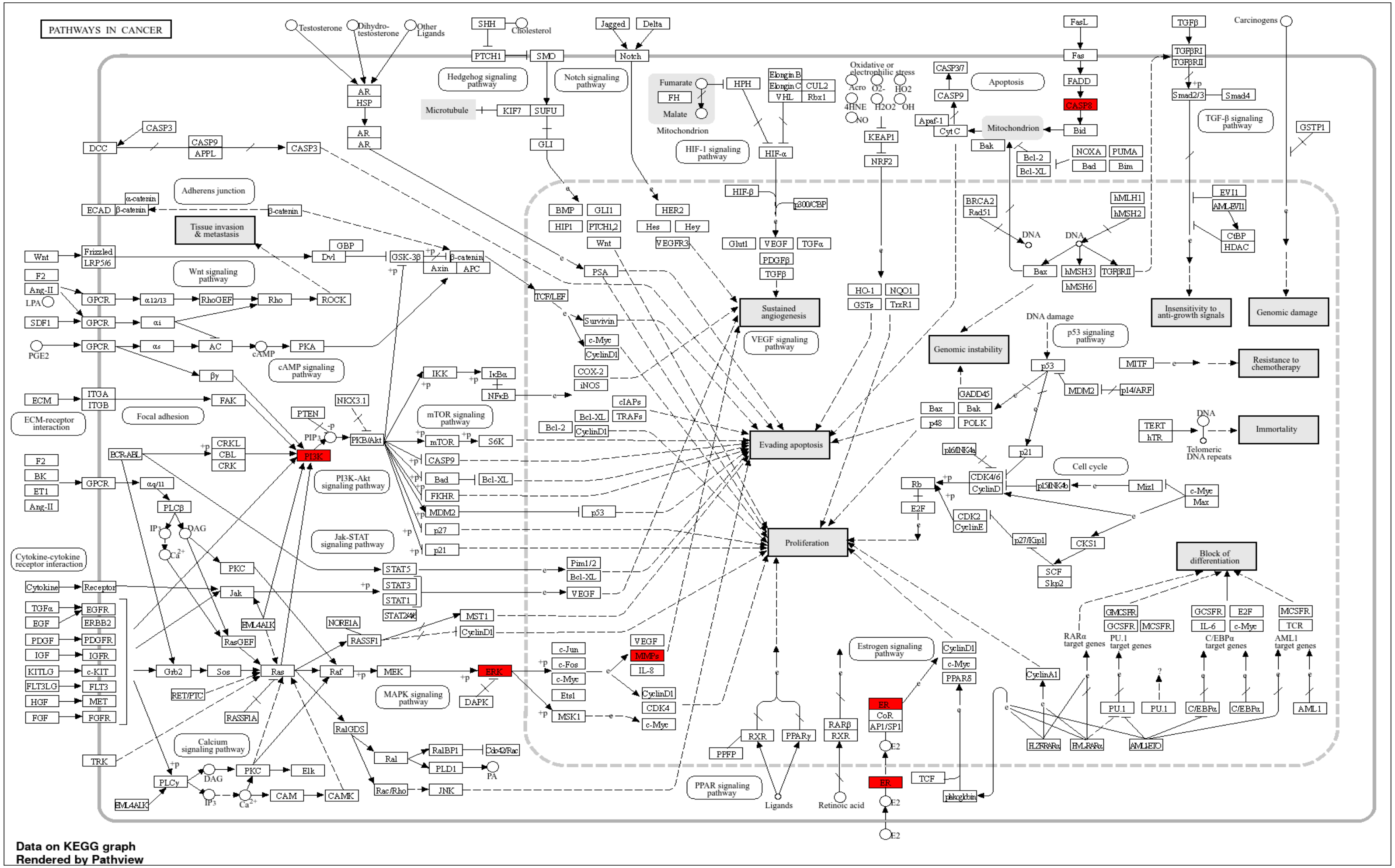

Supplementary Figure S4: pathways in cancer

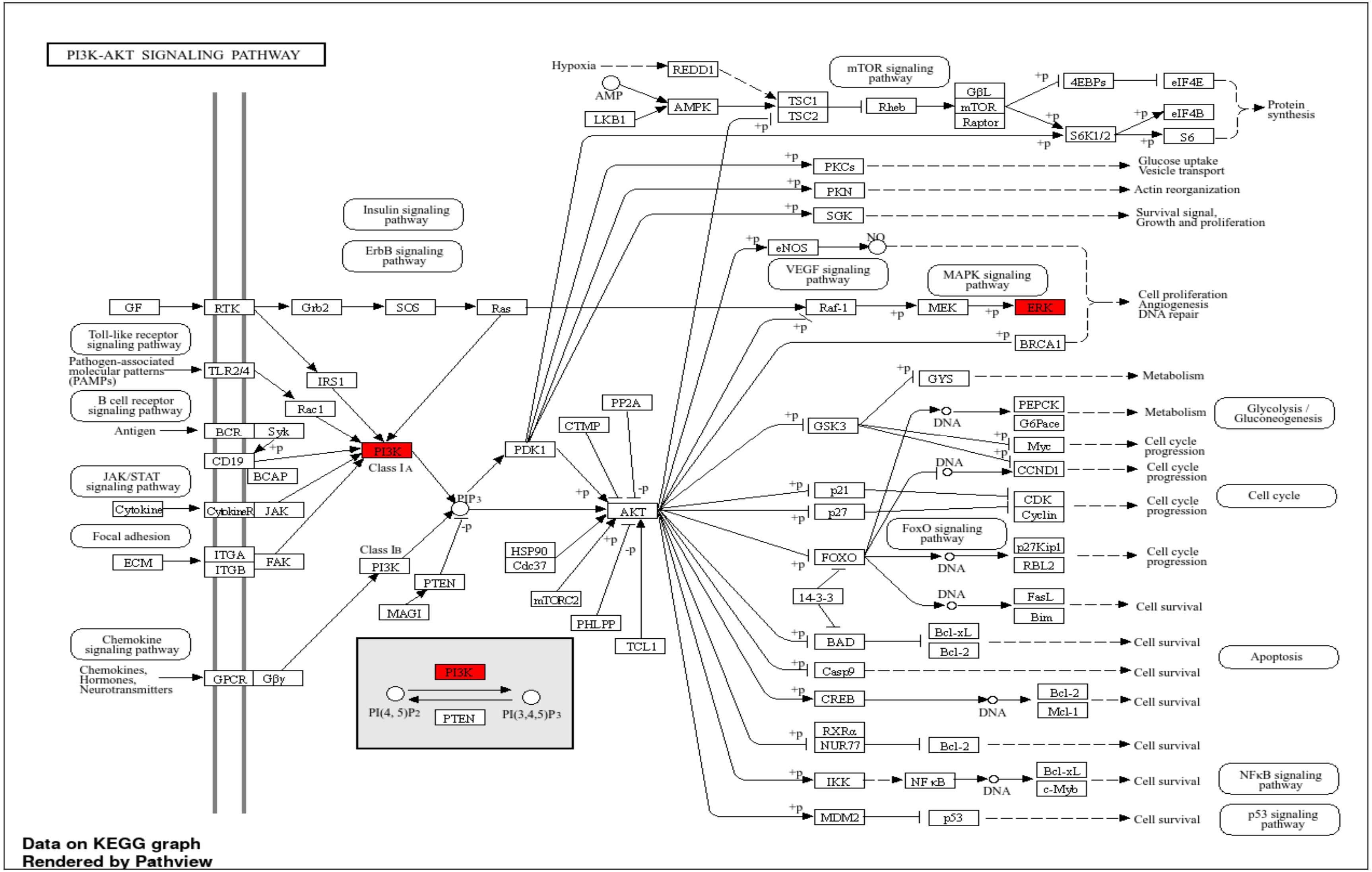

Supplementary Figure S5: PI3K-Akt signal pathway

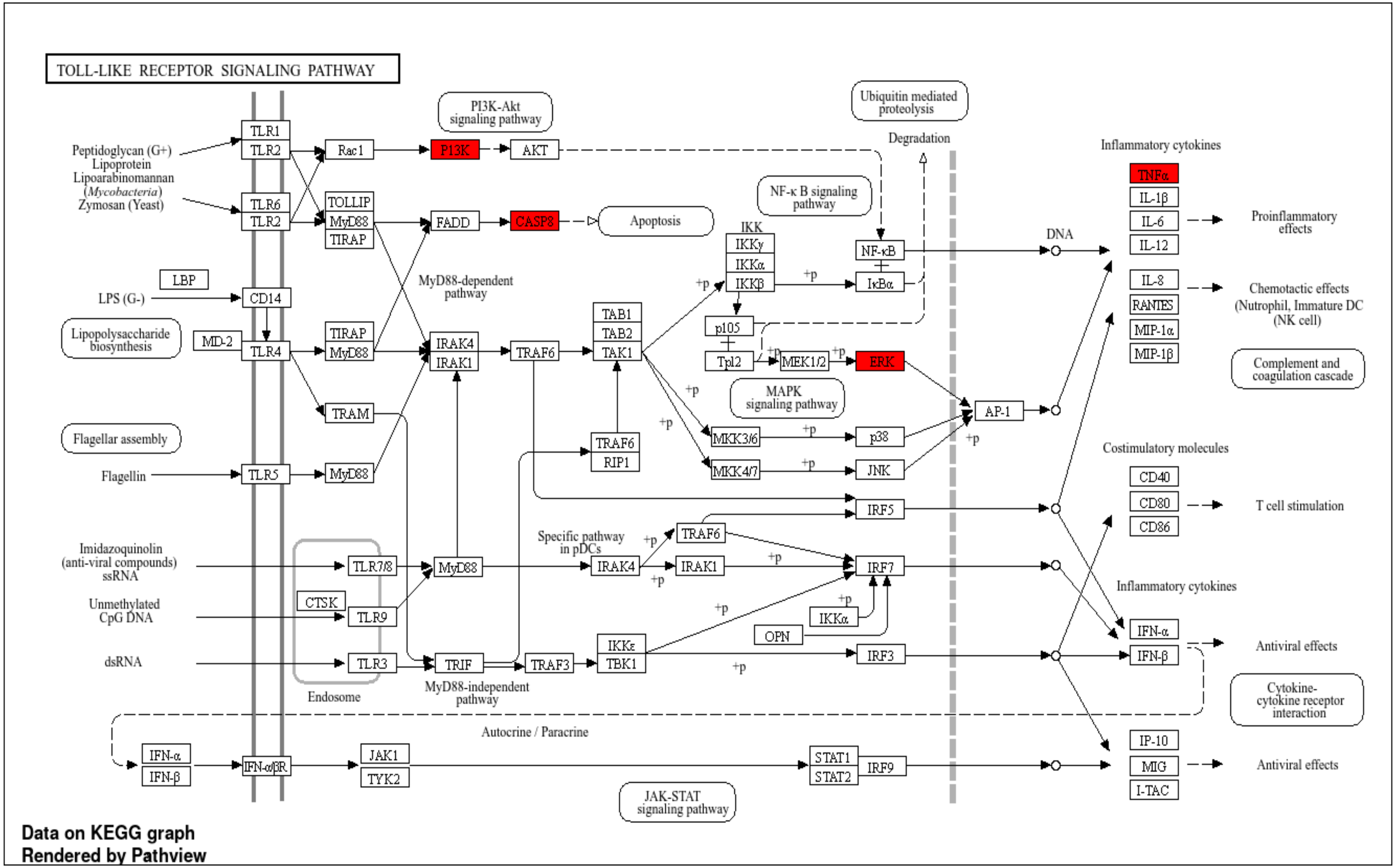

Supplementary Figure S6: Toll-like receptor signaling pathway
